# Exploring endophytic actinomycetes from *Shiraia* fruiting bodies to enhance fungal hypocrellin A biosynthesis

**DOI:** 10.1186/s40643-026-01154-w

**Published:** 2026-10-29

**Authors:** Wen Hao Shen, Li Ping Zheng, Jian Qin Zhou, Jian Wen Wang

**Affiliations:** 1 School of Pharmacy, Jiangsu Medical College, Yancheng, 224005 China; 2 Department of Horticultural Sciences, Soochow University, Suzhou, 215123 China; 3 College of Pharmaceutical Sciences, Soochow University, Suzhou, 215123 China

**Keywords:** *Shiraia*, Actinomycetes, Diversity, Fruiting bodies, Hypocrellin A, Biosynthesis

## Abstract

The medicinal fruiting bodies of *Shiraia* harbor diverse microbial communities that are increasingly recognized as important regulators of fungal secondary metabolism. While endophytic bacteria and fungi associated with *Shiraia* have recently attracted considerable attention, the diversity and functional roles of endophytic actinomycetes remain largely unexplored. Here, we combined culture-dependent isolation with high-throughput 16 S rRNA amplicon sequencing to investigate the endophytic actinomycete community associated with *Shiraia* fruiting bodies. A total of 28 actinomycete isolates representing four genera were recovered, with *Streptomyces* being the predominant culturable genus. Functional screening identified the native endophyte *S. pseudogriseolus* A6 as the most effective elicitor of fungal hypocrellin A (HA) biosynthesis. Genome analysis revealed abundant carbohydrate-active enzymes and 27 secondary metabolite biosynthetic gene clusters, indicating a strong genetic potential for fungal colonization and metabolite-mediated communication. Co-cultivation with live strain A6 markedly enhanced both intracellular and extracellular HA accumulation without significantly affecting fungal biomass. Under the optimized co-cultivation conditions, total HA production reached 296.0 mg/L, representing a threefold increase over the monoculture control. Furthermore, A6 coordinately upregulated the expression of HA biosynthetic genes and metabolite transport genes. This study provides the first characterization of endophytic actinomycetes associated with *Shiraia* fruiting bodies and identifies actinomycete elicitors capable of stimulating fungal HA biosynthesis. These findings expand current knowledge of the *Shiraia* microbiome and highlight endophytic actinomycetes as promising microbial resources for enhancing fungal perylenequinone production.

**Graphical abstract:**

**Figure:**
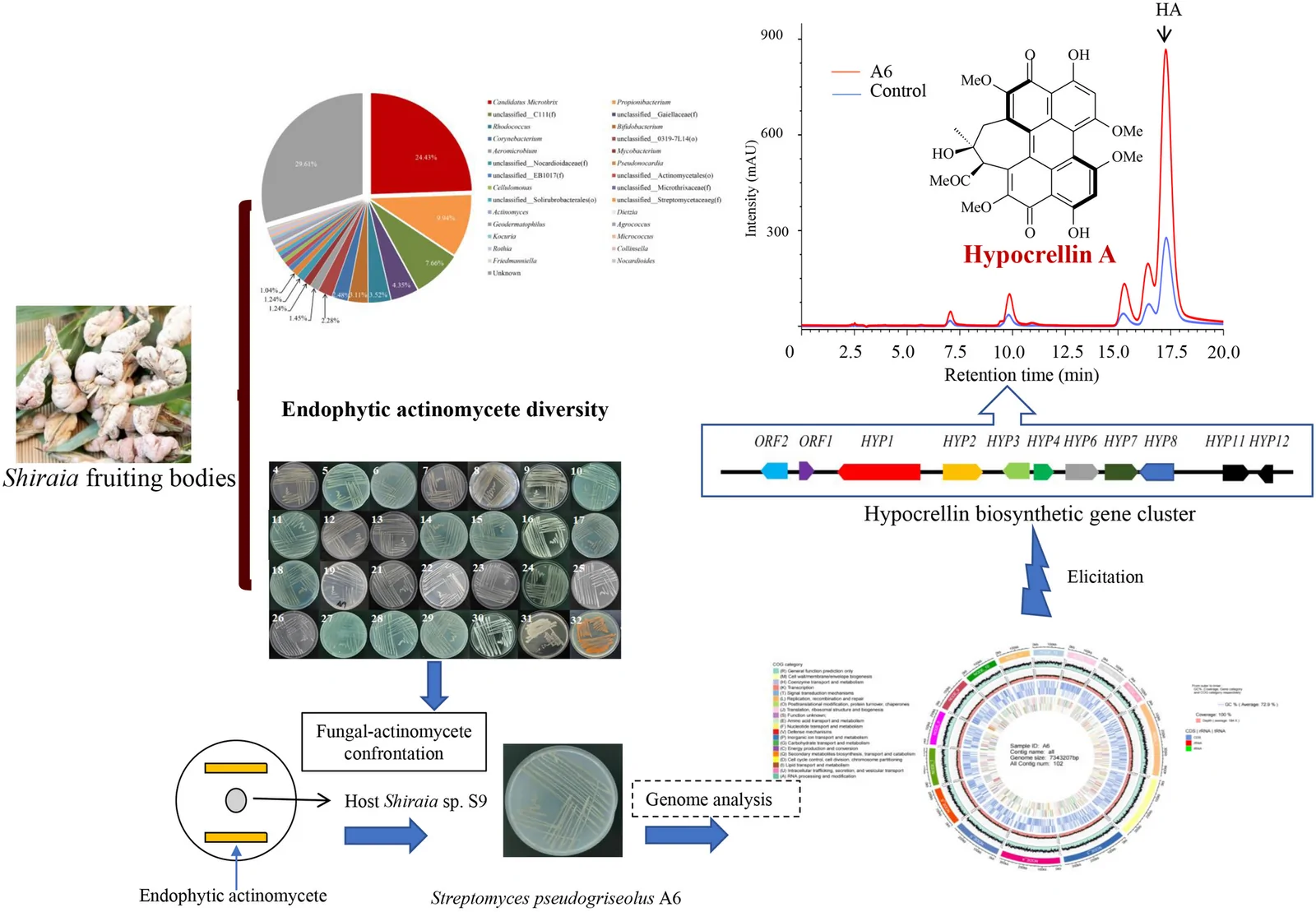

**Supplementary Information:**

The online version contains supplementary material available at https://doi.org/10.1186/s40643-026-01154-w.

## Introduction

*Shiraia bambusicola* is a medicinal bambusicolous fungus that parasitizes living bamboo branches. Its fruiting bodies, known as Zhu Huang in traditional Chinese medicine, have long been used for the treatment of vitiligo, psoriasis, stomachache, and rheumatic pain (Dai et al. 2017). Among the bioactive metabolites produced by *Shiraia*, hypocrellin A (HA), a naturally occurring perylenequinone photosensitizer, is recognized as the principal pharmacologically active constituent of the fruiting bodies (Mulrooney et al. 2012). Owing to its high singlet oxygen quantum yield, favorable photochemical stability, and low dark toxicity, HA has attracted increasing attention as a promising non-porphyrin photosensitizer for photodynamic therapy (PDT) against cancers, infectious diseases, and dermatological disorders (Li et al. 2025). Recent studies have demonstrated that HA-mediated PDT effectively induces reactive oxygen species (ROS)-dependent apoptosis in multiple human cancer cell lines, including ovarian cancer, lung adenocarcinoma, and epithelioid sarcoma cells (Zhang et al. 2024). Beyond cancer therapy, HA also exhibits broad-spectrum antimicrobial activity. It shows potent inhibitory activities against *Pseudomonas aeruginosa*, *Mycobacterium intracellulare*, and methicillin-resistant *Staphylococcus aureus* (MRSA), while lipase-responsive HA-loaded polymeric micelles further enhanced its antibacterial efficacy under visible-light irradiation (Guo et al. 2020; Ma et al. 2004). More recently, hypocrellins have been proposed as promising antiviral photosensitizers against severe acute respiratory syndrome coronavirus 2 (SARS-CoV-2), and subsequent studies confirmed their remarkable antiviral activity with submicromolar IC_50_ and EC_50_ values (Law et al. 2021; Li et al. 2021). In addition, HA has demonstrated considerable potential as an environmentally friendly photoactivated fungicide for controlling several economically important plant diseases (Xing et al. 2003). These expanding biomedical and agricultural applications have substantially increased the demand for HA, highlighting the urgent need for sustainable and efficient production strategies.

The expanding demand for HA has stimulated considerable efforts to establish sustainable biotechnological production systems. Submerged fermentation of *Shiraia* mycelia has emerged as a promising alternative to harvesting wild fruiting bodies. However, hypocrellin production in liquid culture remains relatively low (typically 10–40 mg/L) (Liu et al. 2009), and an economically viable industrial fermentation process has not yet been established. Moreover, the structural complexity of hypocrellins makes total chemical synthesis technically challenging and economically impractical (O’Brien et al. 2010). Consequently, wild *Shiraia* fruiting bodies continue to serve as the major commercial source of hypocrellins. In natural ecosystems, fungal fruiting bodies harbor diverse microbial communities that establish intimate ecological interactions with their hosts. Increasing evidence indicates that these endophytic microorganisms are not merely passive colonizers but actively influence fungal growth, development, stress adaptation, and secondary metabolism through nutrient exchange, signal transduction, and metabolite-mediated communication (Antony-Babu et al. 2014). Therefore, exploiting beneficial microbial interactions has become an emerging strategy for improving fungal metabolite production in biotechnology. Several studies have demonstrated the functional importance of fruiting body-associated microbiota. In *Tricholoma matsutake*, Proteobacteria and Actinobacteria were identified as dominant bacterial groups, and numerous isolates exhibited broad-spectrum antifungal activities (Oh et al. 2018). Similarly, bacterial isolates from *Agaricus bisporus* produced indole-3-acetic acid, cellulases, and phosphate-solubilizing factors that may facilitate host growth (Xiang et al. 2017). Endophytic bacteria isolated from *Rhizopogon roseolus* significantly promoted host mycelial growth (Ayudhya et al. 2019), whereas bacteria associated with *Tuber borchii* produced volatile compounds that stimulated the formation of characteristic truffle aroma metabolites (Splivallo et al. 2015). Collectively, these studies demonstrate that fruiting body-associated microorganisms function as important ecological partners capable of regulating fungal physiology and specialized metabolism. Our previous investigations further demonstrated that the microbiome associated with *Shiraia* fruiting bodies represents an important reservoir of microbial resources with distinct regulatory functions. We isolated 34 endophytic fungal strains belonging to 9 genera, among which *Fusarium* sp. SF12 increased total hypocrellin production to 209.4 mg/L, representing approximately a 1.9-fold increase over the control (Zheng et al. 2025). In addition, 31 endophytic bacterial strains representing 17 species were isolated from *Shiraia* fruiting bodies, with *Bacillus* and *Pseudomonas* constituting the dominant bacterial groups and exhibiting contrasting regulatory effects on hypocrellin biosynthesis (Ma et al. 2019). These findings strongly suggest that host-associated microorganisms play important regulatory roles in hypocrellin production. However, despite the ecological importance of actinomycetes in fungal fruiting bodies, their diversity, functional characteristics, and potential application in regulating hypocrellin biosynthesis remain completely unexplored.

Actinomycetes are widely recognized as prolific producers of bioactive metabolites and extracellular enzymes, and they frequently establish mutualistic associations with plants and fungi. Through the production of signaling molecules, hydrolytic enzymes, siderophores, volatile organic compounds, and other specialized metabolites, actinomycetes can profoundly influence host development and secondary metabolism (Selim et al. 2021). However, despite their ecological importance, the diversity and functional roles of endophytic actinomycetes inhabiting *Shiraia* fruiting bodies have not yet been investigated. More importantly, whether these microorganisms can be exploited as microbial elicitors to enhance hypocrellin biosynthesis remains entirely unknown. We hypothesized that *Shiraia* fruiting bodies harbor previously unexplored endophytic actinomycetes capable of stimulating fungal hypocrellin production through interspecies interactions. To test this hypothesis, we combined culture-dependent isolation with high-throughput amplicon sequencing to characterize the diversity of endophytic actinomycetes associated with *Shiraia* fruiting bodies. Representative isolates were subsequently evaluated for their ability to promote fungal biomass accumulation and hypocrellin production during submerged fermentation. Furthermore, the most effective strain was subjected to whole-genome sequencing to identify functional traits potentially involved in fungal growth promotion and metabolite elicitation. This study provides the first systematic characterization of endophytic actinomycetes associated with *Shiraia* fruiting bodies and demonstrates their potential as microbial resources for enhancing hypocrellin biosynthesis. These findings broaden our understanding of fungus-associated actinomycete communities and provide a new microbial strategy for improving the sustainable biotechnological production of hypocrellins.

## Materials and methods

### Sample collection and surface sterilization

Fruiting bodies of *Shiraia* were collected from naturally infected twigs of *Brachystachyum densiflorum* during June and July 2016 in Tianmu Mountain, Zhejiang Province, China, as described previously (Ma et al. 2019). Thirty healthy fruiting bodies were randomly selected for the isolation of endophytic actinomycetes, with five fruiting bodies constituting one biological replicate. Endophytic actinomycetes were recovered using a combination of surface sterilization and dilution plating according to the method of Ali et al. (2022) with slight modifications. Briefly, the fruiting bodies were first immersed in 0.1% (v/v) Tween 20 for 1 min to remove surface debris, followed by surface disinfection with 5% (w/v) sodium hypochlorite (NaOCl) for 5 min. Residual chlorine was neutralized by incubation in 2.5% (w/v) sodium thiosulfate (Na₂S₂O₃) for 10 min. The samples were subsequently treated with 70% (v/v) ethanol for 5 min and finally immersed in sterile 10% (w/v) sodium bicarbonate (NaHCO₃) for 10 min to suppress the growth of residual fungal propagules. After each sterilization step, the samples were rinsed three times with sterile distilled water. The effectiveness of surface sterilization was evaluated by spreading 0.2 mL of the final rinse water onto TWYE (tap water-yeast extract agar) plates, followed by incubation at 28 °C for 7 days. The absence of microbial growth was taken as confirmation of successful surface sterilization.

### Isolation and identification of endophytic actinomycetes

Following surface sterilization, endophytic actinomycetes were isolated using a dilution plating approach (Atlas 2004). Six selective media, including humic acid–vitamin (HV) agar, TWYE agar, sodium succinate–asparagine (SSA) agar, dulcitol–proline (DP) agar, sodium propionate–asparagine (SPA) agar, and Gause’s synthetic medium No. 1 (GM1), were prepared as summarized in Table S1. All media were adjusted to pH 7.2 before sterilization. Fresh fruiting bodies were aseptically homogenized in sterile water, and appropriate serial dilutions of the tissue homogenates were spread onto the isolation media supplemented with nystatin (50 mg/L) and nalidixic acid (25 mg/L) to suppress fungal and fast-growing bacterial contaminants, respectively (Shan et al. 2018). The plates were incubated at 28 °C for 7–14 days until typical actinomycete colonies appeared. Colonies displaying distinct actinomycete morphology were repeatedly streaked on TWYE agar to obtain pure cultures. Purified isolates were routinely maintained on TWYE agar and preserved in tryptic soy broth (TSB: 15 g/L tryptone, 5 g/L soya peptone, and 5 g/L NaCl) supplemented with 30% (v/v) glycerol at − 20 °C. For seed culture preparation, isolates were inoculated into TSB medium and cultivated at 28 °C with shaking at 150 rpm for 7 days.

Genomic DNA was extracted from harvested mycelia using a bacterial genomic DNA extraction kit (Tiangen Biotech, Beijing, China) according to the manufacturer’s instructions. DNA quality and integrity were evaluated using an Agilent 2100 Bioanalyzer (Agilent Technologies, Santa Clara, CA, USA). The nearly full-length 16 S rRNA gene was amplified using the universal bacterial primer pair 27 F (5′-AGAGTTTGATCATGGCTCAG-3′) and 1492R (5′-TACGGCTACCTTGTTACGACTT-3′) (Li et al. 2016). PCR amplification was performed in a final reaction volume of 30 µL containing 15 µL of 2× Super PCR Mix (BGI Genomics, Beijing, China), 1 µL of genomic DNA, 1 µL of each primer (20 µM), and nuclease-free water to volume. Amplification was carried out using an ABI 9700 thermal cycler (Applied Biosystems, Foster City, CA, USA) under the following conditions: initial denaturation at 96 °C for 5 min; 35 cycles of 96 °C for 20 s, 56 °C for 20 s, and 72 °C for 30 s; followed by a final extension at 72 °C for 10 min. PCR products were sequenced by BGI Genomics (Beijing, China). The resulting 16 S rRNA gene sequences were compared with publicly available sequences for taxonomic assignment, and a phylogenetic tree was constructed using the neighbor-joining algorithm implemented in MEGA version 7.0 (Kumar et al. 2016).

### Amplicon sequencing and bioinformatic analysis

Total community genomic DNA was extracted from surface-sterilized *Shiraia* fruiting bodies as described above. The bacterial 16 S rRNA gene V4–V5 hypervariable region was amplified using the universal primer pair 515 F (5′-GTGCCAGCMGCCGCGG-3′) and 907R (5′-CCGTCAATTCMTTTRAGTTT-3′) (Wu et al. 2015). PCR amplification was performed under the following conditions: an initial denaturation at 96 °C for 3 min, followed by 32 cycles of 95 °C for 30 s, 56 °C for 30 s, and 72 °C for 15 s, with a final extension at 72 °C for 5 min. The amplified products were verified by electrophoresis on a 1% (w/v) agarose gel, purified using a PCR Clean-up Kit (Omega Bio-Tek, Doraville, GA, USA), and quantified using the PicoGreen dsDNA assay (Molecular Probes, Eugene, OR, USA). Sequencing libraries were subsequently prepared according to the manufacturer’s protocol and subjected to paired-end sequencing on the Illumina MiSeq platform (Illumina, San Diego, CA, USA).

Raw sequencing data have been deposited in the NCBI Sequence Read Archive (SRA) under BioProject accession number PRJNA541247. Raw paired-end reads were initially processed using Trimmomatic to remove adapter sequences and low-quality bases (Bolger et al. 2014), followed by read assembly with FLASH (Magoč and Salzberg 2011). After quality filtering, high-quality sequences were clustered into operational taxonomic units (OTUs) at a 97% sequence similarity threshold using CD-HIT (Li and Godzik 2006). Representative sequences from each OTU were taxonomically assigned against the Greengenes 16 S rRNA gene reference database using the RDP Classifier with a confidence threshold of 50%. Rarefaction analysis and alpha-diversity indices were calculated using Mothur (Schloss et al. 2009) to evaluate sequencing depth and microbial community diversity.

### Fungal-actinomycete confrontation assay and submerged co-cultivation

The host fungus *Shiraia* sp. S9 (CGMCC 16369), previously isolated from *Shiraia* fruiting bodies and identified in our earlier study (Ma et al. 2019), was routinely maintained on potato dextrose agar (PDA, Table S1) plates at 28 °C for 10 days. Endophytic actinomycete isolates were cultivated on TWYE agar under the same conditions. For inoculum preparation, each actinomycete isolate was transferred into 30 mL of TSB in a 150-mL Erlenmeyer flask and incubated at 28 °C with shaking at 150 rpm for 7 days. The resulting cultures were adjusted to 2.5 × 10⁸ CFU/mL before use. The interaction between *Shiraia* sp. S9 and individual endophytic actinomycetes was initially evaluated using a dual-culture confrontation assay. Briefly, 10 µL of the standardized actinomycete suspension was streaked onto PDA plates as two parallel lines approximately 7 cm apart, and the fungal inoculum was placed between the two bacterial streaks as previously described (Ma et al. 2019). Fungal cultures receiving an equal volume (10 µL) of sterile TSB instead of the actinomycete suspension served as the control. To evaluate the effects of live endophytic actinomycetes on fungal growth and HA biosynthesis, submerged co-cultivation experiments were performed. Seed cultures of *Shiraia* sp. S9 were prepared following the procedure described by Sun et al. (2017). Briefly, 1 mL of fungal seed culture (approximately 1 × 10⁷ spores/mL) was inoculated into a 150-mL Erlenmeyer flask containing 50 mL of fermentation medium and cultivated at 28 °C on a rotary shaker (ZD-8802, Hualida, Suzhou, China) at 150 rpm. Actinomycete suspensions were added aseptically to the fungal cultures on day 4 to achieve a final concentration of 5 × 10⁶ CFU/mL, and co-cultivation was continued for an additional 4 days. Control cultures received an equal volume of sterile TSB. At the end of the 8-day fermentation, fungal mycelia were harvested by filtration, dried at 60 °C to constant weight, and used for the determination of mycelial biomass and HA production.

### Phylogenetic identification and genome analysis of strain A6

The phylogenetic identification of strain A6 was carried out based on the nearly full-length 16 S rRNA gene sequence as described above. The essential biochemical characteristics including Gram character, glucose utilization, starch hydrolysis, citrate utilization, nitrate reduction, oxidase, catalase, and gelatin liquefaction of the actinomycete have been tested (Krieg and Holt 1984). For whole-genome sequencing, strain A6 was cultivated under the conditions described above, and high-quality genomic DNA was extracted using the same protocol. Qualified genomic DNA was submitted to Sangon Biotech Co., Ltd. (Shanghai, China) for whole-genome sequencing using the Illumina MiSeq platform. Raw sequencing reads were quality-filtered using Trimmomatic version 0.36 (Bolger et al. 2014), and the filtered reads were assembled de novo using SPAdes version 3.5.0 (Bankevich et al. 2012). Genome completeness and assembly statistics were evaluated prior to downstream analyses. Protein-coding genes were predicted with Prodigal (Hyatt et al. 2010), while clustered regularly interspaced short palindromic repeat (CRISPR) loci were identified using the corresponding prediction pipeline. Functional annotation of the predicted genes was performed by sequence homology searches against the Kyoto Encyclopedia of Genes and Genomes (KEGG) and Gene Ontology (GO) databases. Carbohydrate-active enzymes (CAZymes) were identified by comparison with the CAZy database. Biosynthetic gene clusters (BGCs) involved in secondary metabolite production were predicted using antiSMASH version 5.0 operating in the relaxed detection mode (Blin et al. 2019).

### Observation of fungal conidiation and mycelial pellet morphology

Fungal conidia were harvested from 10-day-old PDA cultures by gently washing the colony surface with sterile distilled water. The resulting spore suspension was adjusted as required, and conidial production was determined using a hemocytometer under a light microscope (CX21, Olympus, Tokyo, Japan). To monitor mycelial growth during submerged fermentation, fungal pellets were collected daily from day 1 to day 8 and observed using a stereomicroscope (SMZ1000, Nikon, Tokyo, Japan). Pellet diameter was measured from 50 randomly selected pellets for each biological replicate, with three independent replicates analyzed for each treatment.

### Extraction and quantification of HA

HA was extracted from PDA cultures, fungal mycelia, and fermentation broth according to the procedure previously described by Liu et al. (2018). Briefly, whole cultures grown on PDA medium or fungal mycelia cultivated in liquid medium were dried at 60 °C and ground to pass through a 0.2-mm sieve. A two-gram sample was refluxed with 50 mL of methanol in a water bath at 70 °C for 2 h. The extract was then filtered through a 0.45-µm membrane filter and diluted to 100 mL. HA present in the culture medium was recovered via methylene chloride extraction. The organic solvents were subsequently removed under reduced pressure by rotary evaporation, and the resulting dry extracts were reconstituted in methanol. HA was analyzed using an Agilent 1260 high-performance liquid chromatography (HPLC) system (Agilent Technologies, Wilmington, DE, USA) equipped with an HC-C18 reverse-phase column (250 × 4.6 mm). Separation was performed using a mobile phase consisting of acetonitrile and water (65: 35, v/v) at a flow rate of 1.0 mL/min. The elution was monitored at 465 nm, and the total run time was 20 min (Lei et al. 2017). HA standard was obtained from the Chinese National Compound Library (purity > 98%, Shanghai, China). HA content was calculated based on the corresponding external calibration curve and total HA production was calculated as the sum of intracellular and extracellular HA.

### Quantitative real-time PCR

Total RNA was isolated from fungal mycelia using the RNAprep Pure Plant Kit (Tiangen Biotech, Beijing, China) according to the manufacturer’s instructions. RNA quality and concentration were assessed prior to downstream analysis. First-strand cDNA was synthesized from purified RNA and used as the template for quantitative real-time PCR (qRT-PCR). The fungal 18 S rRNA gene served as the internal reference, and the primer sequences used for qRT-PCR are listed in Table S2. Quantitative real-time PCR was performed following the protocol described by Ma et al. (2018). Relative transcript abundance was calculated using the 2^−ΔΔCt^ method based on the threshold cycle (Ct) values.

### Statistical analysis

Unless otherwise stated, all experiments were performed using three independent biological replicates, with ten plates or flasks included in each replicate. Data are presented as the mean ± standard deviation (SD). Statistical analyses were performed using Student’s *t*-test for comparisons between two groups or one-way analysis of variance (ANOVA) followed by Dunnett’s multiple comparisons test for comparisons among multiple groups. Differences were considered statistically significant at *P* < 0.05.

## Results

### Endophytic actinomycetes associated with *Shiraia* fruiting bodies

**Fig. 1:**
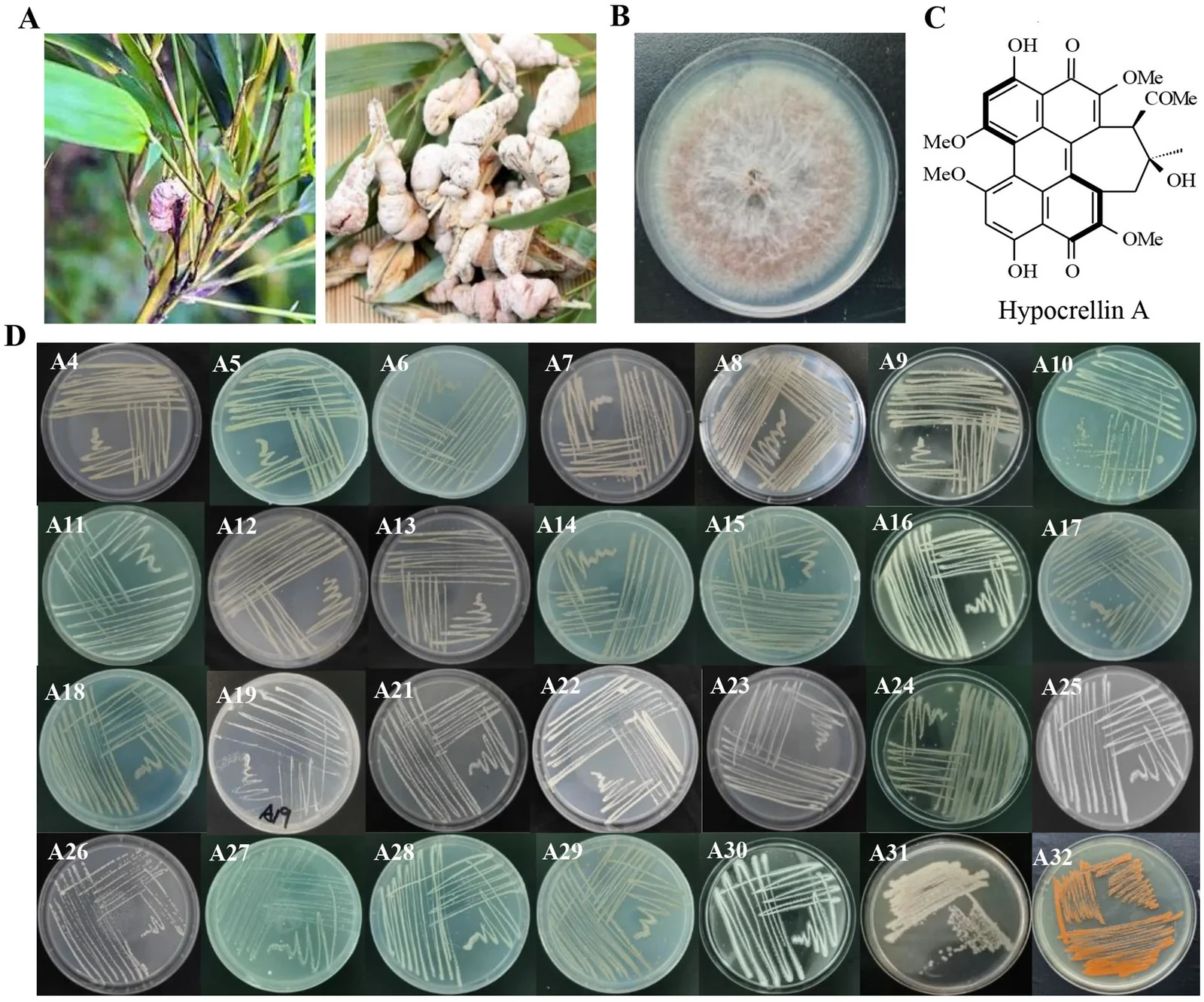
The endophytic actinomycetes isolated from *Shiraia* fruiting bodies. **A** The *Shiraia* fruiting bodies. **B** The colony of *Shiraia* sp. S9. **C** The molecular structure of hypocrellin A (HA). **D** The colony of actinomycetes isolated from *Shiraia* fruiting body

**Fig. 2:**
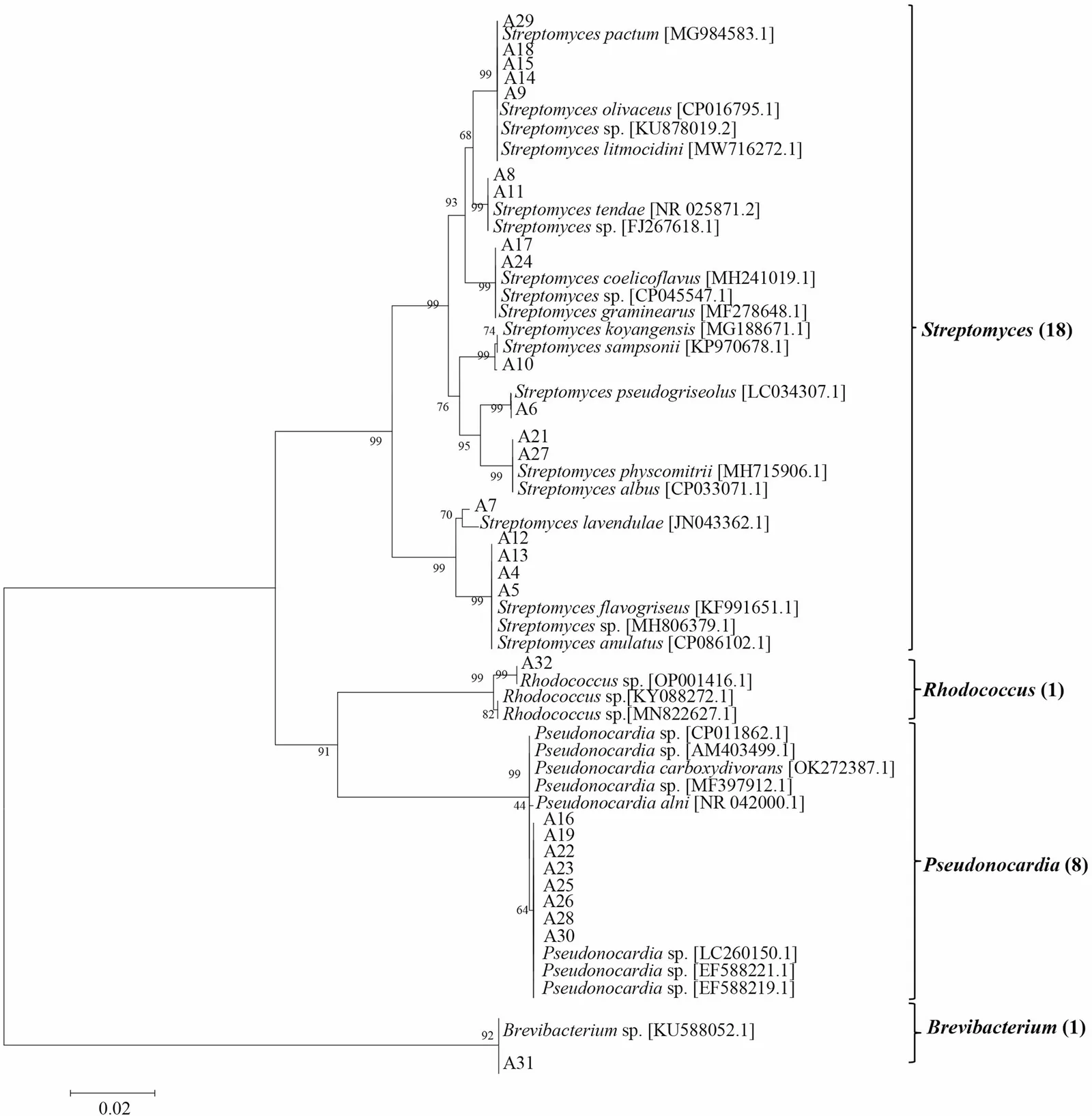
Phylogenetic tree of 16 S rDNA gene sequences showing the relationships between the actinobacteria isolated from *Shiraia* fruiting body and the reference strains. The branch length is proportional to the number of substitutions per site. Scale bar: 0.02 substitutions per nucleotide position. The numbers in the parentheses represent the numbers of isolated actinobacteria

A total of 28 endophytic actinomycete isolates were obtained from freshly collected *Shiraia* fruiting bodies (Fig. 1). The associated host fungus, *Shiraia* sp. S9, was previously identified as a HA-producing strain (Ma et al. 2019). Based on nearly full-length 16 S rRNA gene sequence analysis, the 28 isolates were classified into 2 orders, 4 families, 4 genera, and 11 species (Table S3). Phylogenetic analysis further resolved these isolates into distinct genus-level clades (Fig. 2). Among them, *Streptomyces* was the dominant genus, comprising 18 isolates (64.29%), followed by *Pseudonocardia* with 8 isolates (28.57%), whereas *Brevibacterium* and *Rhodococcus* were each represented by a single isolate (3.57%). BLAST analysis of the 16 S rRNA gene sequences showed that the *Streptomyces* isolates shared 99–100% sequence identity with several validly described species, including *S. pseudogriseolus*, *S. tendae*, *S. venezuelae*, *S. albus*, *S. pactum*, *S. physcomitrii*, and *S. olivaceus*. The remaining isolates were assigned to *Pseudonocardia* spp., *Brevibacterium* sp., and *Rhodococcus* sp., respectively (Table S4). These results demonstrate that *Streptomyces* and *Pseudonocardia* constitute the predominant culturable endophytic actinomycetes associated with *Shiraia* fruiting bodies.

### Endophytic actinomycete communities associated with *Shiraia* fruiting bodies

**Fig. 3:**
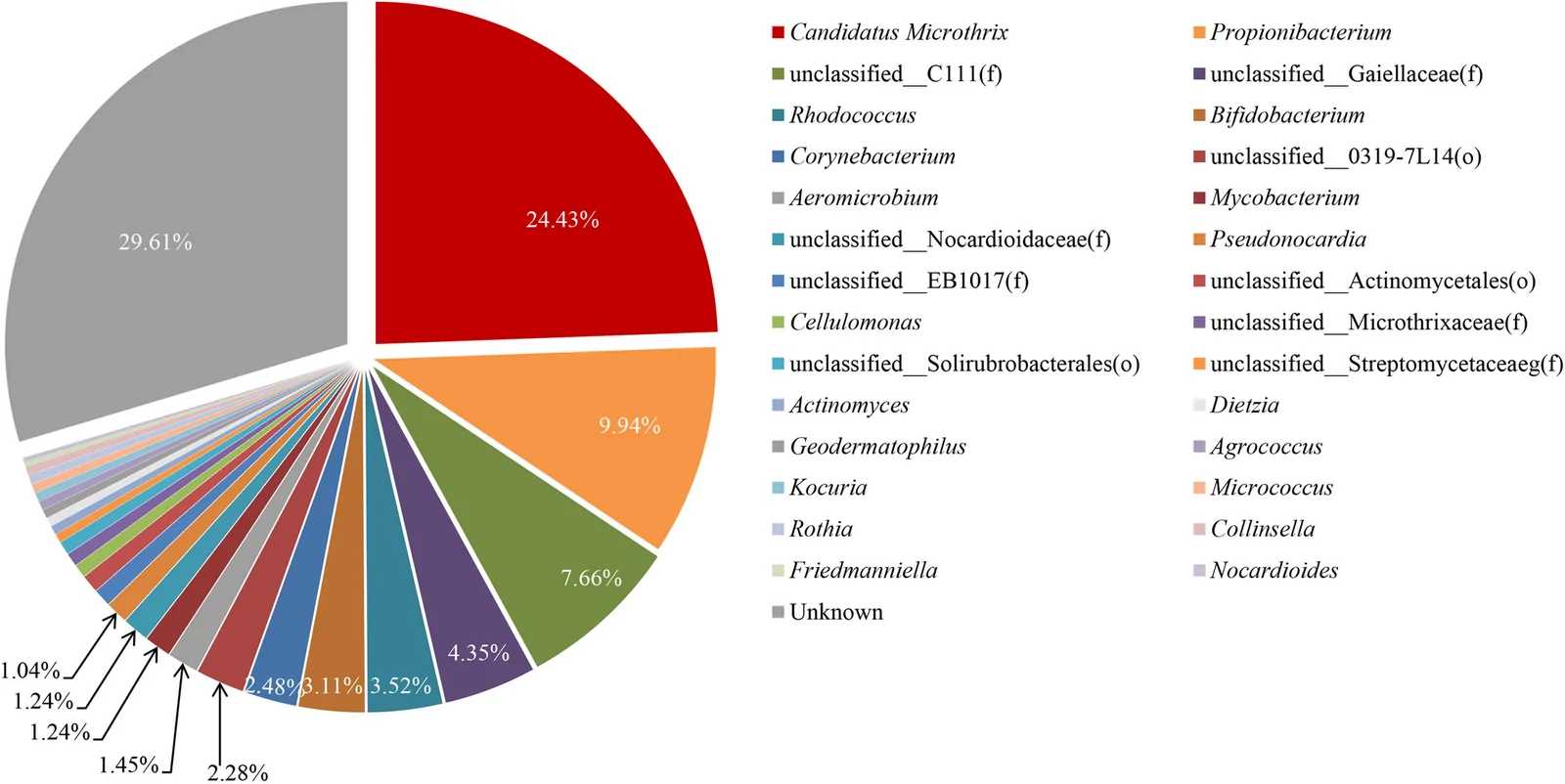
Relative distribution of OTUs of the actinomycete genera in *Shiraia* fruiting body. Sequences are binned at the genus level and the abundance values are depicted as the percentage of the total actinomycete sequences in the sample

High-throughput 16 S rRNA gene amplicon sequencing was performed to characterize the endophytic actinomycete community associated with *Shiraia* fruiting bodies. After quality filtering, 483 high-quality actinomycete reads were obtained from the sample (Table S5). Based on a 97% sequence similarity threshold, these sequences were clustered into 54 operational taxonomic units (OTUs) (Table S6). The Shannon–Wiener and Chao1 indices were 4.17 and 58.01, respectively, indicating a relatively diverse endophytic actinomycete community (Table S7). Taxonomic annotation assigned the 54 OTUs to 5 classes, 8 orders, 24 families, and 28 genera (including unclassified taxa) (Table S8). At the genus level, the community was dominated by *Candidatus Microthrix* (24.43%), followed by *Propionibacterium* (9.94%), *Rhodococcus* (3.52%), *Bifidobacterium* (3.11%), *Corynebacterium* (2.48%), *Aeromicrobium* (1.45%), *Mycobacterium* (1.24%), and *Pseudonocardia* (1.04%) (Fig. 3; Table S9).

Comparison of the culture-dependent and culture-independent approaches revealed both shared and distinct taxonomic profiles. *Pseudonocardia* and *Rhodococcus* were consistently detected by both methods, supporting their stable association with *Shiraia* fruiting bodies. In contrast, marked differences were observed in the relative abundance of several taxa. Culture-dependent isolation was dominated by *Streptomyces* (64.29%) and *Pseudonocardia* (28.57%), whereas members of the family Streptomycetaceae represented only 0.41% of the sequences recovered by high-throughput sequencing (Table S9). In addition, *Brevibacterium* was recovered exclusively by the culture-dependent approach. These results demonstrate that culture-dependent isolation and amplicon sequencing provide complementary views of the endophytic actinomycete community associated with *Shiraia* fruiting bodies, highlighting the importance of integrating both approaches for microbial resource exploration.

### Effects of endophytic actinomycetes on fungal HA production

**Fig. 4:**
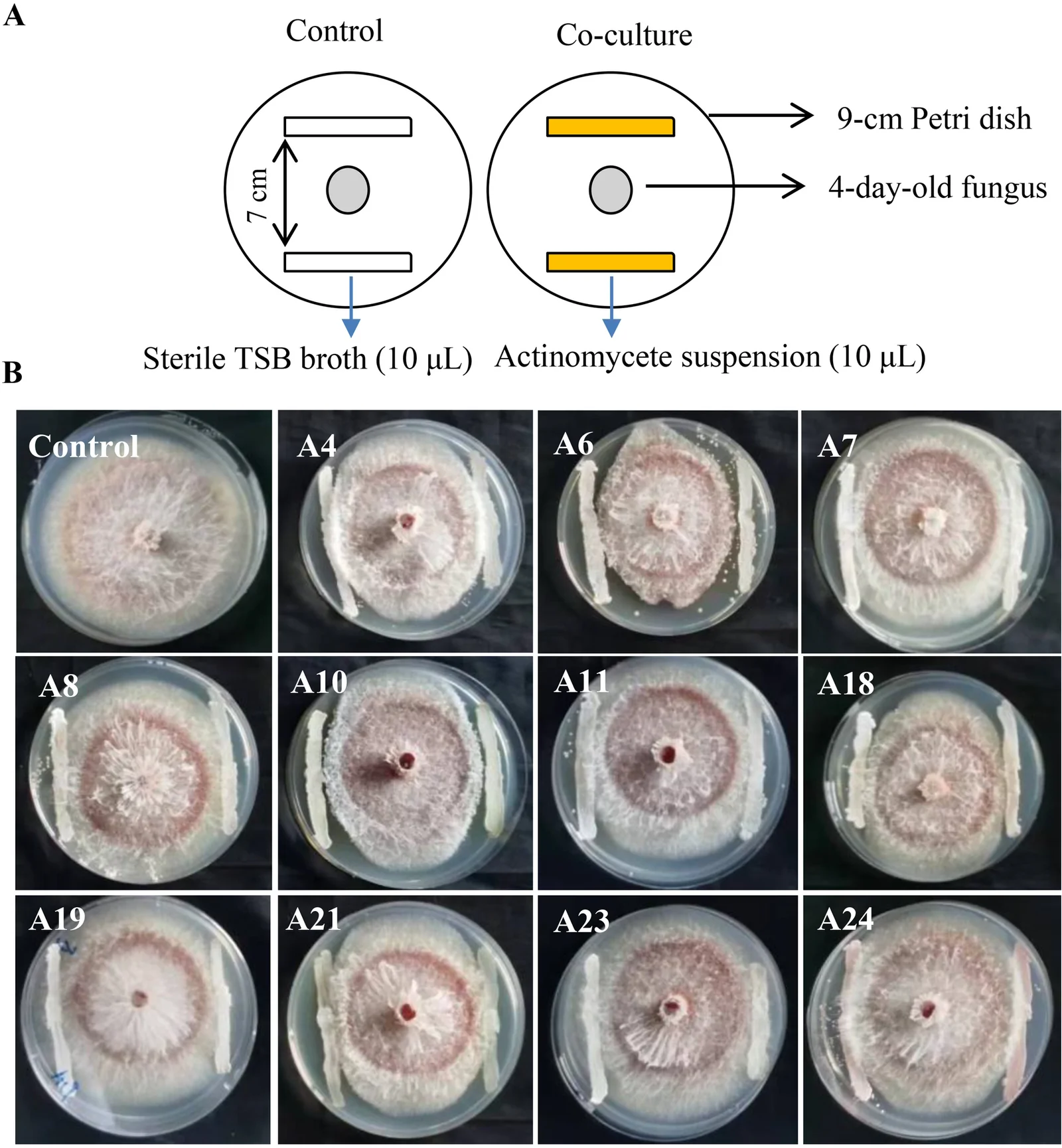
The mode diagrams (**A**) and plate cultures for the confrontation tests of endophytic actinomycete and fungus *Shiraia* sp. S9 (**B**). The S9 strain treated with the equal volume of sterile TSB broth instead of live actinomycete suspension was used as control group and co-culture indicates the cultivation of actinomycetes and *Shiraia* sp S9. The actinomycete suspension (10 µL of 2.5 × 10^8^ CFU/mL) was added to the PDA plate of *Shiraia* sp S9 on day 4 during the cultures of 8 days

To evaluate the regulatory effects of endophytic actinomycetes on fungal HA biosynthesis, all isolates were initially screened using a fungal–actinomycete confrontation assay (Fig. 4A). Distinct responses were observed among the tested isolates. Several strains, including A6, A8, A11, and A23, markedly promoted the secretion of red pigments by *Shiraia* sp. S9 (Fig. 4B). Since HA has been confirmed as the main component of the red pigments produced by *Shiraia* fungi (Yang et al. 2009), these results provide preliminary evidence for the ability of the isolates to enhance HA production. Accordingly, HA in the solid-state culture and mycelium culture of *Shiraia* sp. S9 was subsequently quantified using an HPLC method (Table 1). Among the tested isolates, strain A6 exhibited the strongest stimulatory effect on HA production. In co-cultures of strain A6 with *Shiraia* sp. S9, HA accumulation increased significantly under both solid and submerged culture conditions. The HA content reached 0.5 mg/plate on PDA and 14.7 mg/g dry weight (DW) in submerged mycelial cultures, representing 2.0-fold and 2.3-fold increases over the respective controls (Table 1). In contrast, several isolates, including A4, A13, A19, and A31, significantly suppressed HA production by *Shiraia* sp. S9. These results demonstrate that endophytic actinomycetes exert strain-dependent effects on fungal HA biosynthesis, with strain A6 showing the greatest potential as a positive elicitor for subsequent mechanistic investigation.

**Table 1:** Effect of actinomycetes from *Shiraia* fruiting bodies on fungal hypocrellin A (HA) accumulation of *Shiraia* sp. S9 in cultures

Strain	HA content	
	PDA plate (mg/plate)	liquid culture (mg/g DW)
Control^a^	0.23 ± 0.03	6.27 ± 0.27
A4	0.21 ± 0.08	3.85 ± 1.06*
A5	0.24 ± 0.06	5.38 ± 0.35
A6	0.46 ± 0.05**	14.65 ± 1.94**
A7	0.35 ± 0.12	10.17 ± 1.13*
A8	0.37 ± 0.01**	11.26 ± 2.02*
A9	0.33 ± 0.04*	6.06 ± 1.88
A10	0.25 ± 0.06	7.52 ± 3.77
A11	0.31 ± 0.09	9.87 ± 2.81
A12	0.21 ± 0.03	4.40 ± 0.94**
A13	0.17 ± 0.04	3.05 ± 0.48**
A14	0.34 ± 0.06*	5.25 ± 0.75
A15	0.40 ± 0.09*	5.79 ± 1.47
A16	0.30 ± 0.03*	4.41 ± 0.66*
A17	0.32 ± 0.11	4.71 ± 3.77
A18	0.40 ± 0.07*	5.82 ± 1.55
A19	0.18 ± 0.02**	2.86 ± 0.95**
A21	0.41 ± 0.12	5.37 ± 0.76
A22	0.35 ± 0.09	10.84 ± 0.76**
A23	0.44 ± 0.05**	10.25 ± 1.89*
A24	0.23 ± 0.06	5.15 ± 1.47
A25	0.39 ± 0.07*	8.05 ± 0.30
A26	0.28 ± 0.02	4.58 ± 0.63*
A27	0.35 ± 0.01**	5.27 ± 0.97
A28	0.30 ± 0.06	4.53 ± 1.13
A29	0.26 ± 0.03	5.80 ± 1.32
A30	0.23 ± 0.01	4.59 ± 0.36*
A31	0.09 ± 0.01**	3.92 ± 0.39**
A32	0.17 ± 0.04	6.16 ± 0.24

^a^The actinomycete was introduced on day 4 of cultivation, and untreated cultures served as controls. Values are mean ± SD from three independent experiments. *: *P* < 0.05; **: *P* < 0.01

### The identification and genomic analysis of active A6 strain

**Fig. 5:**
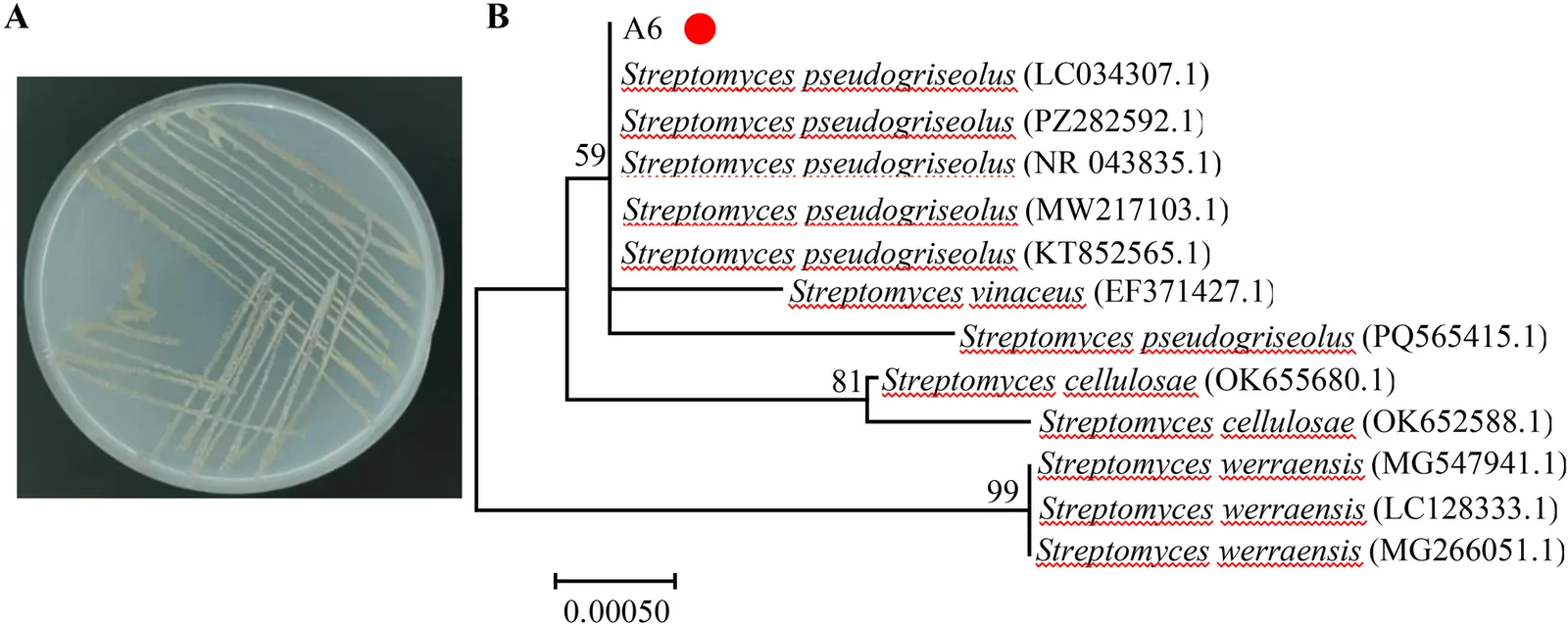
The identification of active endophytic actinomycete A6. **A** The colony morphology. **B** Phylogenetic tree of sequencing result

Strain A6 formed dry, compact colonies with a gray aerial mycelium on TWYE agar (Fig. 5A), and its physiological and biochemical characteristics are summarized in Table S10. A nearly full-length 16 S rRNA gene sequence (1,075 bp) was obtained and used for phylogenetic analysis. The phylogenetic tree showed that strain A6 clustered closely with *S. pseudogriseolus*, sharing 99.86% sequence identity with *S. pseudogriseolus* strain LC034307.1 (Fig. 5B). Based on its morphological characteristics and 16 S rRNA gene sequence analysis, strain A6 was identified as *S. pseudogriseolus* A6.

**Fig. 6:**
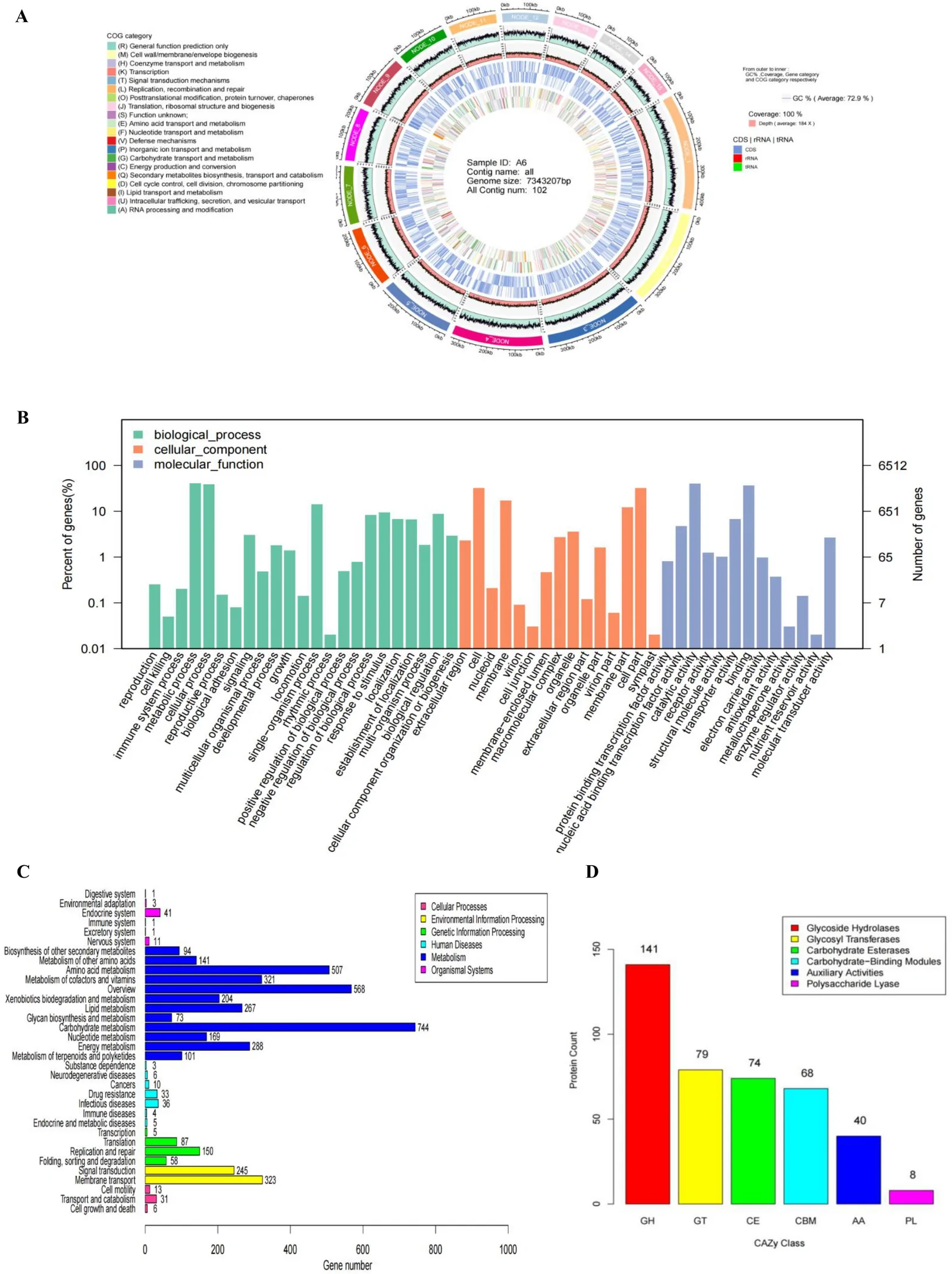
Genomic map (**A**), GO functional classification map (**B**), KEGG metabolic pathway secondary classification map (**C**) and carbohydrate-active enzymes (**D**) of *S. pseudogriseolus* A6. GH, glycoside Hydrolases; GT, glycosyl transferases; CE, carbohydrate esterases; AA, auxiliary activities; CBM, carbohydrate-binding modules; PL, polysaccharide lyase

To gain insight into the genetic basis of its interaction with *Shiraia*, the genome of *S. pseudogriseolus* A6 was sequenced and annotated. The genome comprised 7,343,207 bp with a GC content of 72.9%, containing 6,644 predicted protein-coding genes (Fig. 6A). Functional annotation assigned 3,998 genes (61.39%) to Gene Ontology (GO) categories (Fig. 6B), including cellular component (2,262 genes), molecular function (3,627 genes), and biological process (3,112 genes). Within these categories, membrane-associated components predominated in the cellular component category, metabolic processes represented the largest biological process group (2,627 genes), whereas catalytic activity (2,615 genes) and binding (2,378 genes) were the most abundant molecular function terms. A total of 2,457 genes (37.73%) were assigned to Kyoto Encyclopedia of Genes and Genomes (KEGG) pathways (Fig. 6C). The most highly represented pathways included ABC transporters (297 genes), two-component regulatory systems (229 genes), carbon metabolism (208 genes), and biosynthesis of amino acids (159 genes), suggesting extensive metabolic and environmental response capabilities.

Genome-wide CAZyme annotation identified 410 carbohydrate-active enzyme (CAZyme) genes (Fig. 6D), comprising 141 glycoside hydrolases (GHs), 79 glycosyltransferases (GTs), 74 carbohydrate esterases (CEs), 68 carbohydrate-binding modules (CBMs), 40 auxiliary activities (AAs), and 8 polysaccharide lyases (PLs). Glycoside hydrolases represented the largest CAZyme class (34.39%), including eight putative chitinase genes (four GH18 and GH19 family members), indicating a substantial repertoire of fungal cell wall-degrading enzymes. Genome mining further predicted 27 secondary metabolite biosynthetic gene clusters (BGCs) (Fig. S1), including those encoding polyketides (PKs), non-ribosomal peptides (NRPSs), and terpenes (Table S11). Twelve BGCs shared 50–100% similarity with previously characterized clusters, including those responsible for the biosynthesis of antimycin, naphthyridinomycin, and carotenoids, whereas the remaining 15 clusters exhibited less than 50% similarity, suggesting considerable biosynthetic novelty (Table S11). Collectively, the genome of *S. pseudogriseolus* A6 revealed abundant genes involved in environmental adaptation, carbohydrate metabolism, and secondary metabolite biosynthesis, providing a genomic framework for subsequent investigation of its interaction with *Shiraia*.

### Effect of live *S. pseudogriseolus* A6 on fungal growth and morphology

**Fig. 7:**
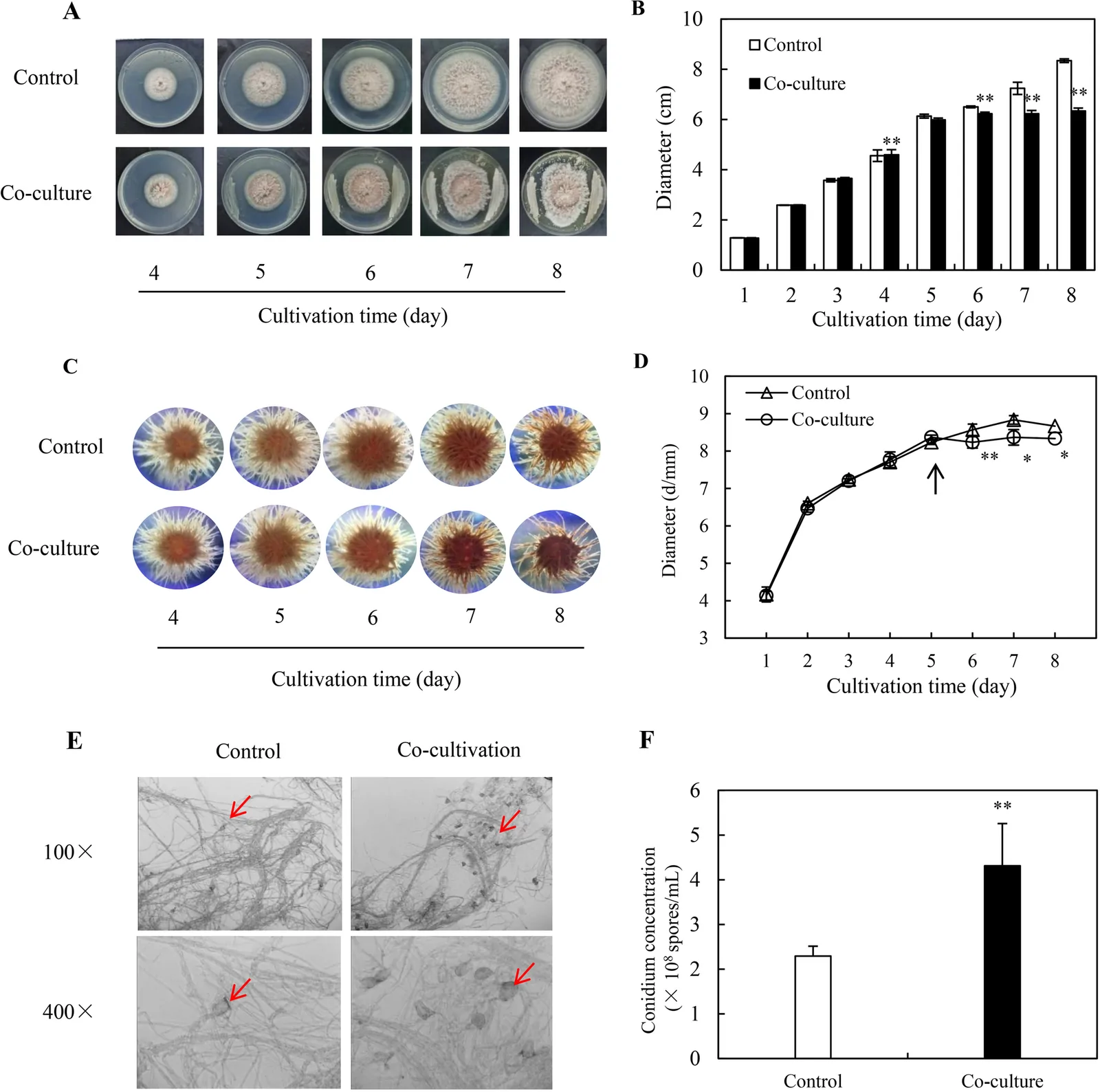
The effects of *S. pseudogriseolus* A6 on the fungal growth of *Shiraia* sp. S9. Time profiles of mycelial growth (**A**) and average colony diameters (**B**) in the solid-plate culture. The effects of *S. pseudogriseolus* A6 on the morphology (**C**) and diamenter (**D**) of mycelium pellets (15 ×) in submerged culture. Live *S. pseudogriseolus* A6 (5.0 × 10^6^ CFU/mL) was added on day 5 of the culture at 150 rpm and 28℃. The effects of *S. pseudogriseolus* A6 on fungal pycnidium (**E**) and conidium concentration (**F**). The procedure of fungal-actinomycete confrontation assay was the same as specified in Fig. 4. Red arrow indicates pycnidium. An equal volume of sterile TSB broth instead of A6 suspension added to flask was used as control group. Values are mean ± SD from three independent experiments (^*^P < 0.05, ^**^P < 0.01 versus control group)

To evaluate the influence of live *S. pseudogriseolus* A6 on fungal development, morphological changes in *Shiraia* sp. S9 were examined following co-cultivation. Colony expansion was significantly inhibited in the confrontation assay, with fungal colony diameters markedly reduced from day 4 to day 8 compared with the control (Fig. 7A, B). In submerged cultures, the presence of strain A6 markedly altered mycelial morphology. The fungal pellets became smaller and more compact than those of the control cultures (Fig. 7C), and pellet diameters were significantly reduced during days 6–8 (Fig. 7D). Despite the reduced colony growth and pellet size, fungal asexual reproduction was significantly promoted. Conidial production increased markedly, and the conidial concentration rose from 2.29 × 10^8^ to 4.31 × 10^8^ spores/mL following inoculation with strain A6 (Fig. 7E, F). These results indicate that live *S. pseudogriseolus* A6 markedly remodels fungal growth and developmental morphology.

### Effect of *S. pseudogriseolus* A6 on fungal HA production

**Fig. 8:**
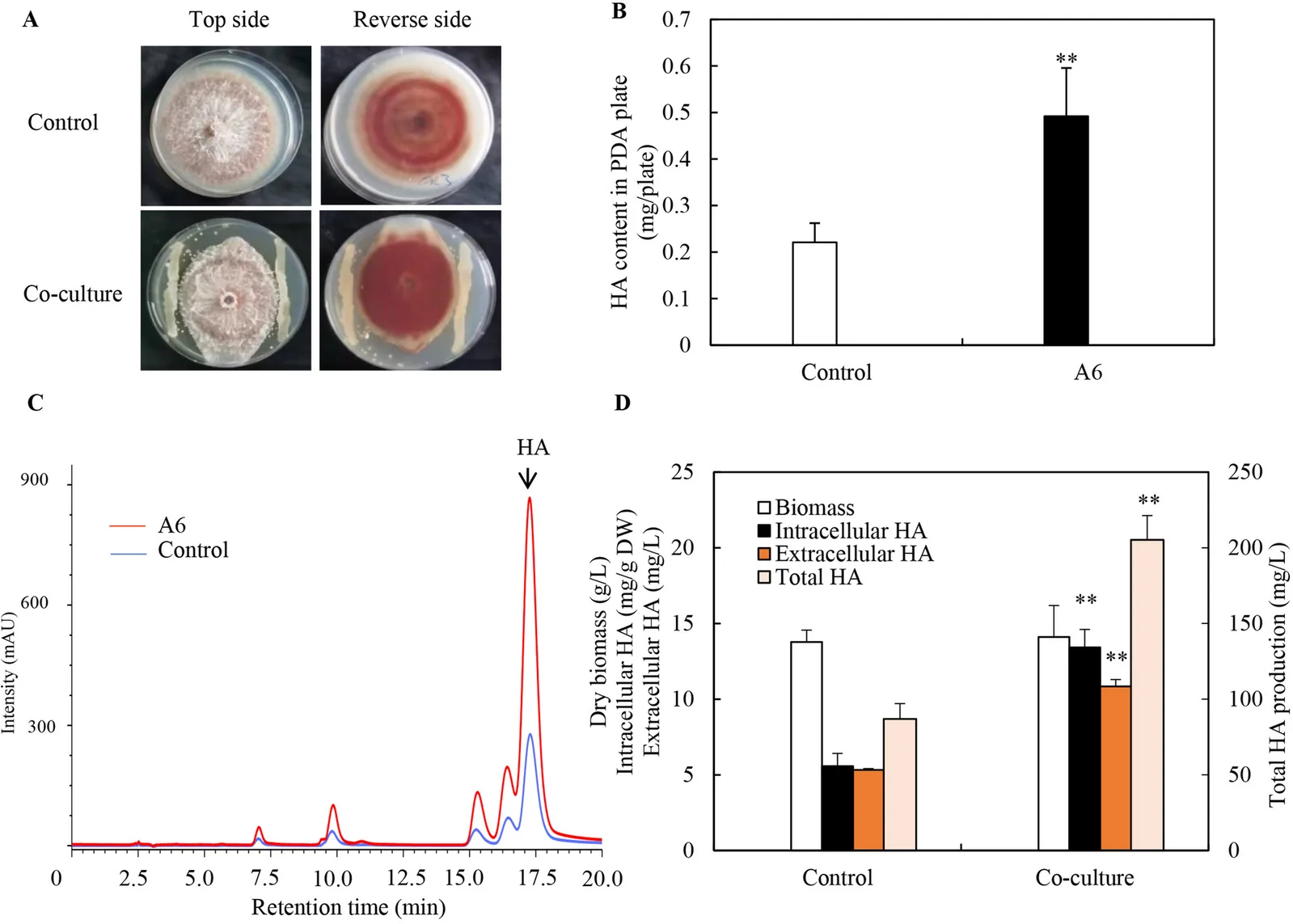
Effects of *S. pseudogriseolus* A6 on hypocrellin A (HA) accumulation of *Shiraia* sp. S9. (**A**) Macroscopic colony appearance of fungal-actinomycete confrontation on PDA for 4 days. (**B**) HA content in the PDA plate. (**C**) The chromatogram of the enhanced HA treated with the actinomycete in *Shiraia* mycelium cultures. (**D**) Effect of the actinomycete on fungal biomass, intracellular-, extracellular- and total HA production of the fungus. Live actinomycete (5.0 × 10^6^ CFU/mL) was added on day 4 of the culture at 150 rpm and 28℃ and the mycelia were harvested on day 8. The control group was treated with the equal volume of sterile TSB broth instead of live actinomycete suspension (^**^P < 0.01 versus control group)

The stimulatory effect of live *S. pseudogriseolus* A6 on fungal HA production was first evaluated on PDA plates. Compared with the control, co-cultivation with strain A6 markedly enhanced the accumulation of the characteristic red HA pigment surrounding the fungal colony (Yang et al. 2009), resulting in an HA content of 0.50 mg/plate, representing a 2.2-fold increase over the control (Fig. 8A, B). The elicitation effect was further confirmed in submerged mycelial cultures by HPLC analysis (Fig. 8C). Although fungal biomass remained comparable to that of the control, both intracellular and extracellular HA production were significantly enhanced following inoculation with strain A6 (Fig. 8D). On day 8, intracellular HA content reached 13.4 mg/g DW, representing a 2.4-fold increase compared with the control. Extracellular HA accumulation in the culture broth was also significantly elevated (2.0-fold). Consequently, total HA production increased to 205.1 mg/L, approximately 2.4-fold higher than that of the control cultures. Together, these findings demonstrate that live *S. pseudogriseolus* A6 efficiently stimulates fungal HA biosynthesis without significantly affecting fungal biomass.

### Effects of *S. pseudogriseolus* A6 on the expression of HA biosynthetic genes

**Fig. 9:**
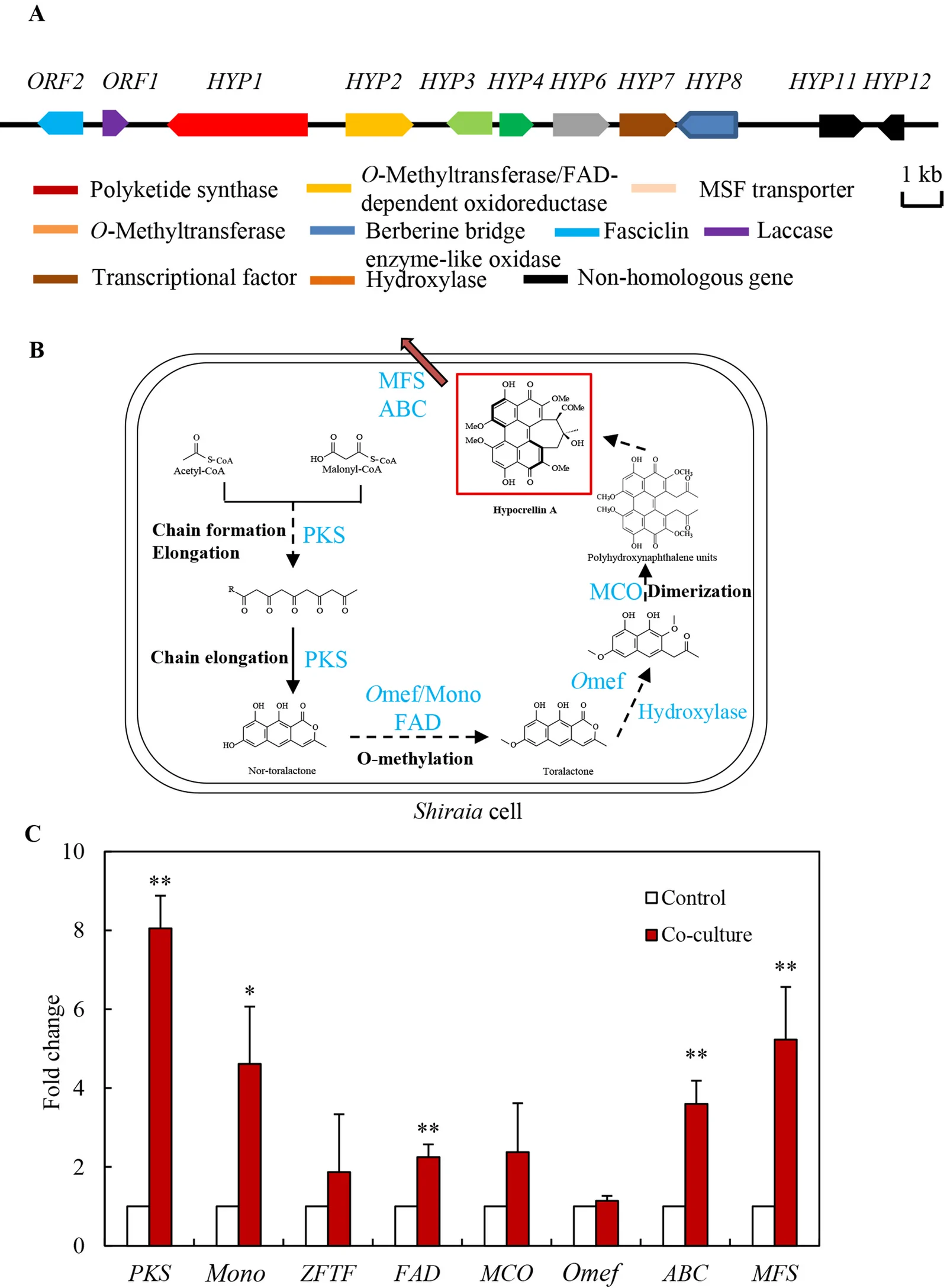
Effects of *S. pseudogriseolus* A6 on gene expressions for HA biosynthesis in *Shiraia* sp. S9. **A** The biosynthetic gene cluster *HYP* for hypocrellins. **B** The schematic diagram of *S. pseudogriseolus* A6-induced fungal HA biosynthesis. **C** *S. pseudogriseolus* A6-induced expressions of key genes for HA biosynthesis. Live *S. pseudogriseolus* A6 (5.0 × 10^6^ CFU/mL) was added on day 5 of the mycelium culture at 150 rpm and 28℃ and the mycelia were harvested on day 8. The culture of *Shiraia* sp. S9 treated with the equal volume of sterile TSB broth instead of live actinomycete suspension was used as control group. *PKS* polyketide synthase, *Omef* O-methyltransferase, *FAD* FAD/FMN-dependent oxidoreductase, *Mono* monooxygenase, *MCO* multicopper oxidase, *MFS* major facilitator superfamily, *ABC* ATP-binding cassette transporter and *ZFTF* zinc finger transcription factor. Values are mean ± SD from three independent experiments (^∗^*P* < 0.05, ^∗∗^*P* < 0.01 versus control group)

To elucidate the molecular basis underlying the enhanced HA production induced by *S. pseudogriseolus* A6, the transcriptional responses of genes involved in the HA biosynthetic pathway were analyzed by quantitative real-time PCR. Candidate genes were selected according to the reported HA biosynthetic gene cluster in *Shiraia* spp. (Deng et al. 2022), including genes encoding polyketide synthase (*PKS*), monooxygenase (*Mono*), FAD-dependent oxidoreductase (*FAD*), multicopper oxidase (*MCO*), *O*-methyltransferase (*Omef*), zinc finger transcription factor (*ZFTF*), ATP-binding cassette (*ABC*) transporter, and major facilitator superfamily (*MFS*) transporter (Fig. 9A). Co-cultivation with live *S. pseudogriseolus* A6 significantly up-regulated the expression of all examined HA biosynthetic genes (Fig. 9B). Among them, *PKS*, encoding the key enzyme responsible for polyketide backbone formation, exhibited the strongest transcriptional response, with an 8.1-fold increase compared with the control. Significant induction was also observed for *Mono* (4.6-fold), *MFS* (5.2-fold), *ABC* (3.6-fold), *MCO* (2.4-fold), *FAD* (2.3-fold), *ZFTF* (1.9-fold), and *Omef* (1.1-fold) (Fig. 9C). Collectively, these results demonstrate that live *S. pseudogriseolus* A6 coordinately activates the transcription of biosynthetic, regulatory, and transporter genes, consistent with the enhanced HA accumulation observed during co-cultivation.

### Optimization of co-cultivation for enhanced HA production

**Fig. 10:**
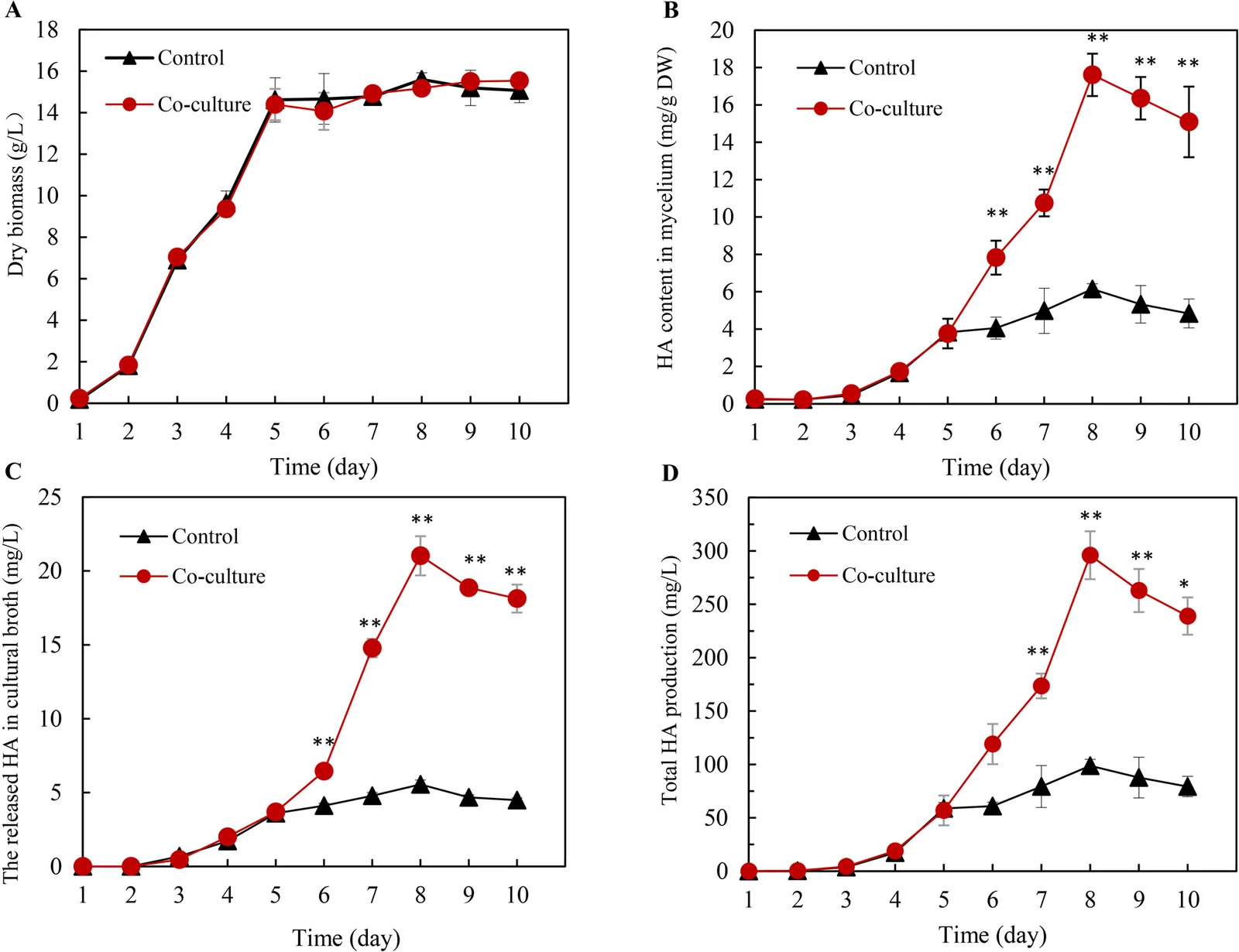
The time-course of fungal growth and HA production in the co-cultivation of *Shiraia* sp. S9 with *S. pseudogriseolus* A6. **A** Fungal biomass. **B** HA content in mycelium. **C** The released HA in cultural broth. **D** The total HA production. Total HA production refers to the sum of the intracellular and extracellular HA. *Shiraia* sp. S9 was treated with live A6 (4.0 × 10^6^ CFU/mL) on day 5 and incubated at 150 rpm and 28℃. The S9 strain treated with the equal volume of sterile TSB broth instead of live actinobacterial suspension was used as control group. Values are mean ± SD from three independent experiments (^*^P < 0.05, ^**^P < 0.01 versus control group)

To maximize HA production during fungal–actinomycete co-cultivation, the effects of inoculation time and inoculum concentration of *S. pseudogriseolus* A6 were systematically evaluated. The timing of A6 inoculation markedly influenced fungal growth and HA production (Fig. S2). When 5.0 × 10^6^ CFU/mL of live A6 was introduced during the early growth stage (days 0–3), both fungal biomass and HA production were significantly inhibited. In contrast, inoculation at the late exponential phase (day 5) significantly enhanced both intracellular and extracellular HA accumulation, resulting in a total HA production of 181.0 mg/L. The effect of inoculum concentration was subsequently investigated by adding different concentrations of live A6 (1.0–6.0 × 10^6^ CFU/mL) on day 5 (Fig. S3). Fungal biomass remained essentially unchanged over the tested concentration range, whereas HA production was significantly stimulated at inoculum concentrations between 2.0 × 10^6^ and 5.0 × 10^6^ CFU/mL. The highest HA production (258.8 mg/L) was achieved at an inoculum concentration of 4.0 × 10^6^ CFU/mL, representing a 2.8-fold increase over the control. Based on these results, the optimal co-cultivation strategy was determined as inoculation of *S. pseudogriseolus* A6 at 4.0 × 10^6^ CFU/mL on day 5. Under these optimized conditions, fungal biomass remained comparable to that of the control throughout cultivation, whereas both intracellular and extracellular HA accumulation increased continuously (Fig. 10). Total HA production reached 296.0 mg/L on day 8, corresponding to an approximately 3.0-fold increase relative to the control. These findings establish an optimized fungal–actinomycete co-cultivation strategy for efficient HA production and provide a practical basis for future bioprocess development.

## Discussion

### Endophytic actinomycetes represent a new microbial resource in *Shiraia* fruiting bodies

Fungal fruiting bodies constitute unique ecological niches that harbor diverse microbial communities involved in nutrient cycling, host development, and secondary metabolism (Antony-Babu et al. 2014; Xiang et al. 2017). Among these microorganisms, actinomycetes are increasingly recognized as important fungal associates because of their exceptional metabolic versatility and their ability to establish intimate ecological interactions with macrofungi. Previous investigations have demonstrated that *Streptomyces* species are common inhabitants of fruiting bodies of several edible and medicinal mushrooms, including *T. borchii*, *A. bisporus*, and *Suillus grevillei* (Varese et al. 1996; Sbrana et al. 2002; Xiang et al. 2017). Similarly, culture-independent analyses of black truffle microbiota revealed that actinomycetes constitute one of the predominant bacterial groups colonizing fruiting bodies (Antony‐Babu et al. 2014). Our study provides the first comprehensive characterization of endophytic actinomycetes associated with *Shiraia* fruiting bodies. Culture-dependent isolation yielded 28 actinomycete strains representing four genera, with *Streptomyces* accounting for more than 64% of all isolates, whereas high-throughput sequencing uncovered a substantially broader actinomycete diversity, including numerous uncultured taxa. The marked discrepancy between culture-dependent and culture-independent analyses is consistent with previous reports describing selective cultivation bias and the existence of viable but non-culturable microorganisms in fungal tissues (Kataoka et al. 2012; Pent et al. 2017). Nevertheless, both approaches consistently detected *Pseudonocardia* and *Rhodococcus*, suggesting that these genera are stable members of the *Shiraia* fruiting-body microbiome.

Our previous studies have characterized the bacterial and fungal communities associated with *Shiraia* fruiting bodies (Ma et al. 2019; Zheng et al. 2025). The present work further extends these investigations by specifically focusing on endophytic actinomycetes and demonstrating, for the first time, that these actinomycetes actively regulate fungal secondary metabolism. Because *Shiraia* fruiting bodies are the principal natural source of hypocrellins, which have attracted increasing attention as photoactive agents for photodynamic therapy, understanding their associated microbiota provides new opportunities for both ecological studies and microbial resource exploitation.

### The interaction between *S. pseudogriseolus* A6 and its fungal host

Unlike randomly associated environmental bacteria, endophytic actinomycetes inhabiting fungal fruiting bodies have coexisted with their hosts during tissue development and are therefore more likely to evolve species-specific metabolic communication (Varese et al. 1996; Sbrana et al. 2002). Such long-term ecological associations may enable these bacteria to influence fungal growth, development, and secondary metabolism without causing extensive host damage. Consistent with this ecological perspective, *S. pseudogriseolus* A6 markedly altered the developmental phenotype of *Shiraia* sp. S9. Co-cultivation with the actinomycete significantly suppressed vegetative mycelial growth, reduced pellet size, and promoted conidiation, while simultaneously stimulating HA biosynthesis. Similar growth-inhibitory effects have been reported for *Streptomyces* isolates associated with the ectomycorrhizal fungus *S. grevillei*, suggesting that modulation of fungal development may represent an ecological strategy employed by endophytic actinomycetes (Varese et al. 1996). Interestingly, although fungal growth was partially restrained in the present study, HA accumulation increased substantially, indicating that the interaction between strain A6 and *Shiraia* primarily reprogrammed fungal secondary metabolism rather than simply affecting biomass production. HA possesses a 3,10-dihydroxy-4,9-perylenequinone chromophore that absorbs light energy and generates ROS, thereby contributing to plant cell damage (Khiralla et al. 2022). *Shiraia* species producing HA have been reported as pathogens of more than 10 bamboo species in China and Japan (Morakotkarn et al. 2008). Unlike some antagonistic bacteria that suppress fungal growth or toxin biosynthesis (Hiranmayee et al. 2025), *S. pseudogriseolus* A6 suppressed the vegetative mycelial growth of *Shiraia* sp. S9 while markedly promoting HA biosynthesis. These findings suggest that *S. pseudogriseolus* A6 can act as an elicitor of fungal HA biosynthesis, although its growth-inhibitory effect may also reflect an antagonistic or stress-related component of the interaction. Although our pairwise co-culture system provided valuable initial insights into the metabolic and physiological responses of *Shiraia* sp. S9 to *S. pseudogriseolus* A6, it represents a simplified model of the complex microbial interactions occurring within natural *Shiraia* fruiting bodies. Therefore, the interactions observed in vitro may not fully reflect the ecological and biochemical relationships in situ. Further studies integrating Fluorescence In Situ Hybridization (FISH) and Confocal Laser Scanning Microscopy (CLSM) could help characterize the spatial distribution and co-localization of *S. pseudogriseolus* A6 and *Shiraia* within fruiting bodies (Partida-Martinez and Hertweck 2005), while Matrix-Assisted Laser Desorption/Ionization Mass Spectrometry Imaging (MALDI-MSI) could provide spatial information on metabolites associated with their interactions (Moree et al. 2012).

The genome sequence of *S. pseudogriseolus* A6 provides further insight into the molecular basis of this interaction. Functional annotation revealed an extensive repertoire of carbohydrate-active enzymes (CAZymes), with glycoside hydrolases representing the largest enzyme family. In particular, the presence of multiple putative chitinase genes belonging to the GH18 and GH19 families suggests that strain A6 possesses the genetic capacity to modify fungal cell wall components. Chitinases produced by *Streptomyces* species are well known to participate in fungal cell wall remodeling and microbial competition, while moderate cell wall perturbation can also function as an elicitation signal that activates fungal defense responses and specialized metabolism (Taechowisan et al. 2003). Although the contribution of individual chitinases remains to be experimentally verified, the abundant CAZyme repertoire supports the possibility that extracellular enzymes participate in the communication between strain A6 and its fungal host. Besides hydrolytic enzymes, *Streptomyces* species are renowned producers of structurally diverse secondary metabolites that mediate interspecies interactions. Genome mining identified 27 biosynthetic gene clusters (BGCs) in strain A6, including clusters encoding polyketides, nonribosomal peptides, terpenes, and several clusters showing low similarity to known biosynthetic pathways. Previous studies have demonstrated that bacterial metabolites can serve as elicitors to activate otherwise silent fungal biosynthetic pathways or redirect metabolic flux toward specialized metabolite production (Scherlach and Hertweck 2009; Brakhage and Schroeckh 2011). Although the specific signaling molecules produced by strain A6 remain unidentified, the genomic evidence presented here strongly supports its potential to modulate fungal physiology through multiple biochemical mechanisms.

### Coordinated activation of the hypocrellin biosynthetic pathway underlies enhanced HA production

Hypocrellin biosynthesis has recently been elucidated through genome sequencing and functional characterization of the biosynthetic gene cluster in *Shiraia* species (Deng et al. 2020, 2022). In this pathway, the non-reducing polyketide synthase PKS catalyzes the initial condensation of acetyl-CoA and malonyl-CoA to generate the aromatic polyketide backbone, which subsequently undergoes a series of oxidative modifications, methylation, dimerization, and transport to produce mature hypocrellins. Previous studies demonstrated that overexpression of *PKS* markedly increased hypocrellin production, confirming its central role in controlling pathway flux (Deng et al. 2020). Consistent with these findings, *PKS* exhibited the strongest transcriptional induction in response to *S. pseudogriseolus* A6 in the present study, suggesting that activation of the initial committed biosynthetic step contributes substantially to the increased HA accumulation. Importantly, genes encoding tailoring enzymes, including Mono, FAD, MCO, and Omef, were simultaneously up-regulated during co-cultivation. These enzymes are responsible for oxidative rearrangement, methylation, and structural maturation of the perylenequinone scaffold, indicating that the enhanced HA production resulted from activation of the complete biosynthetic pathway rather than accumulation of metabolic intermediates. In parallel, the increased expression of *ZFTF* suggests that fungal–actinomycete interaction may influence pathway-specific transcriptional regulation, thereby amplifying the expression of downstream biosynthetic genes. Although the upstream signaling events remain unknown, these results indicate that the elicitation by strain A6 occurs at the transcriptional level and affects multiple functional modules within the HA biosynthetic gene cluster.

Besides biosynthesis itself, efficient secretion of fungal secondary metabolites is essential for preventing intracellular accumulation and maintaining metabolic homeostasis. The significant induction of both *ABC* and *MFS* transporter genes observed in this study provides a plausible explanation for the simultaneous increase in intracellular and extracellular HA accumulation. *ABC* and *MFS* transporters are widely involved in exporting fungal secondary metabolites, detoxification, and self-resistance against toxic natural products (Stergiopoulos et al. 2002). Their coordinated up-regulation therefore suggests that *Shiraia* not only synthesized larger amounts of HA but also enhanced its transport capacity to accommodate increased metabolite production. Such synchronized regulation of biosynthesis and metabolite export likely contributes to the substantial improvement in total HA yield achieved during co-cultivation. This coordinated regulation provides molecular evidence that endophytic actinomycetes can reprogram fungal specialized metabolism through modulation of the entire biosynthetic network rather than individual catalytic steps.

### Biotechnological implications of fungal–actinomycete co-cultivation for hypocrellin production

Microbial co-cultivation has emerged as an effective strategy for activating silent biosynthetic pathways and enhancing the production of valuable natural products by mimicking ecological interactions that occur in natural habitats (Lyu et al. 2022; Selegato and Castro-Gamboa 2023). Previous studies from our group demonstrated that co-cultivation of *Shiraia* sp. S9 with the endophytic bacterium *Pseudomonas fulva* SB1 enhanced hypocrellin production by approximately 2.4-fold (Ma et al. 2025), while interaction with the endophytic fungus *Fusarium* sp. SF12 increased HA yield to 209.5 mg/L (Zheng et al. 2025). In the present study, co-cultivation with the endophytic actinomycete *S. pseudogriseolus* A6 resulted in the highest HA production of 296.0 mg/L, representing an approximately 3.0-fold increase over the monoculture control. More importantly, optimization of the inoculation time and bacterial density further demonstrated that the efficiency of microbial elicitation strongly depends on the developmental stage of the fungal culture, emphasizing the importance of precisely regulating microbial interactions during fermentation. These findings suggest that naturally associated endophytic actinomycetes may represent more effective elicitors than previously investigated bacterial or fungal partners for enhancing hypocrellin biosynthesis.

Compared with abiotic elicitation strategies previously developed for *Shiraia* cultures, including light regulation (Sun et al. 2018; Wang et al. 2024), ultrasound stimulation (Sun et al. 2017), surfactant treatment (Lei et al. 2017), and temperature modulation (Wen et al. 2022), the fungal–actinomycete co-culture system established in this study offers several practical advantages. The approach requires no specialized equipment or complex operating procedures and relies on a naturally occurring biological interaction that simultaneously promotes intracellular biosynthesis and extracellular secretion of HA. Such coordinated enhancement of metabolite synthesis and export is particularly advantageous for downstream product recovery and may reduce the metabolic burden associated with intracellular accumulation of photoactive perylenequinones. These characteristics make microbial co-cultivation an attractive strategy for developing sustainable fermentation processes for industrial hypocrellin production. Beyond improving metabolite yield, fungal-actinomycete interactions may provide broader opportunities for exploring microbial chemical ecology and activating cryptic fungal biosynthetic pathways. Genome mining revealed that *S. pseudogriseolus* A6 possesses numerous putative secondary metabolite biosynthetic gene clusters, suggesting its capacity to produce diverse signaling molecules capable of modulating fungal metabolism. Future studies integrating comparative transcriptomics, metabolomics, and functional genetics will help identify the bacterial elicitors and fungal signaling pathways responsible for the observed transcriptional activation of the hypocrellin biosynthetic gene cluster. Such mechanistic investigations will not only deepen our understanding of cross-kingdom microbial communication but may also facilitate the discovery of previously uncharacterized photoactive perylenequinones and other valuable fungal natural products.

## Conclusions

Overall, this study demonstrates that naturally associated endophytic actinomycetes are not merely components of the *Shiraia* fruiting-body microbiome but function as effective regulators of fungal specialized metabolism. The discovery of *S. pseudogriseolus* A6 and the establishment of an optimized fungal–actinomycete co-cultivation strategy provide both mechanistic insight into microbial regulation of hypocrellin biosynthesis and a practical platform for improving fungal biomanufacturing. These findings highlight endophytic actinomycetes as valuable microbial resources for sustainable production of medicinal perylenequinones. This work also establishes a foundation for exploiting ecological microbial interactions to enhance the sustainable production of medicinal perylenequinones and other valuable fungal natural products.

## Supplementary Information

Below is the link to the electronic supplementary material.

Supplementary Material 1

## Data Availability

Data will be made available on request.

## References

[CR1] Ali AR, Bahrami Y, Kakaei E, Mohammadzadeh S, Bouk S, Jalilian N (2022) Isolation and identification of endophytic actinobacteria from *Citrullus colocynthis* (L.) Schrad and their antibacterial properties. Microb Cell Fact 21(1):206. 10.1186/s12934-022-01936-9 PMC9548430 36217205

[CR2] Antony-Babu S, Deveau A, Van Nostrand JD, Zhou JZ, Le Tacon F, Robin C, Frey-Klett P, Uroz S (2014) Black truffle‐associated bacterial communities during the development and maturation of *Tuber melanosporum* ascocarps and putative functional roles. Environ Microbiol 16(9):2831–2847. 10.1111/1462-2920.12294 24118660

[CR3] Atlas RM (2004) Handbook of microbiological media. CRC Press, Boca Raton. 10.1201/EBK1439804063

[CR4] Ayudhya SPN, Riffiani R, Ozaki Y, Onda Y, Nakano S, Aimi T, Shimomura N (2019) Isolation of bacteria from fruiting bodies of *Rhizopogon roseolus* and their effect on mycelial growth of host mushroom. Mushroom Sci Biotechnol 27(4):134–139. 10.24465/msb.27.4_134

[CR5] Bankevich A, Nurk S, Antipov D, Gurevich AA, Dvorkin M, Kulikov AS, Lesin VM, Nikolenko SI, Pham S, Prjibelski AD (2012) SPAdes: a new genome assembly algorithm and its applications to single-cell sequencing. J Comput Biol 19(5):455. 10.1089/cmb.2012.0021 PMC3342519 22506599

[CR6] Blin K, Shaw S, Steinke K, Villebro R, Ziemert N, Lee SY, Medema MH, Weber T (2019) AntiSMASH 5.0: updates to the secondary metabolite genome mining pipeline. Nucleic Acids Res 47(W1):W81–W87. 10.1093/nar/gkz310 PMC6602434 31032519

[CR7] Bolger AM, Lohse M, Usadel B (2014) Trimmomatic: a flexible trimmer for Illumina sequence data. Bioinformatics 30(15):2114–2120. 10.1093/bioinformatics/btu170 PMC4103590 24695404

[CR8] Brakhage AA, Schroeckh V (2011) Fungal secondary metabolites—Strategies to activate silent gene clusters. Fungal Genet Biol 48:15–22. 10.1016/j.fgb.2010.04.004 20433937

[CR9] Dai DQ, Phookamsak R, Wijayawardene NN, Li WJ, Bhat DJ, Xu JC, Taylor JE, Chukeatirote E (2017) Bambusicolous fungi. Fungal Divers 82:1–105. 10.1007/s13225-016-0367-8

[CR10] Deng HX, Liang WY, Fan TP, Zheng XH, Cai YJ (2020) Modular engineering of *Shiraia bambusicol*a for hypocrellin production through an efficient CRISPR system. Int J Biol Macromol 165:796–803. 10.1016/j.ijbiomac.2020.09.208 33010268

[CR11] Deng HX, Liang WY, Liu JB, Zheng XH, Fan TP, Cai YJ (2022) Advances and perspectives on perylenequinone biosynthesis. Front Microbiol 13:1070110. 10.3389/fmicb.2022.1070110 PMC9808054 36605511

[CR12] Guo LY, Yan SZ, Tao X, Yang Q, Li Q, Wang TS, Yu SQ, Chen SL (2020) Evaluation of hypocrellin A-loaded lipase sensitive polymer micelles for intervening methicillin-resistant *Staphylococcus aureus* antibiotic-resistant bacterial infection. Mat Sci Eng C 106:110230. 10.1016/j.msec.2019.110230 31753349

[CR13] Hiranmayee G, Mallick SP, Reddy GS (2025) Antagonistic potential and analytical profiling of plant probiotic bacteria using chromatography and mass spectrometry techniques against *Botrytis cinerea* and *Fusarium oxysporum*. Bioresour Bioprocess 12:67. 10.1186/s40643-025-00853-0 PMC12181468 40542243

[CR14] Hyatt D, Chen GL, LoCascio PF, Land ML, Larimer FW, Hauser LJ (2010) Prodigal: prokaryotic gene recognition and translation initiation site identification. BMC Bioinform 11:119. 10.1186/1471-2105-11-119 PMC2848648 20211023

[CR15] Kataoka R, Siddiqui ZA, Kikuchi J, Ando M, Sriwati R, Nozaki A, Futai K (2012) Detecting nonculturable bacteria in the active mycorrhizal zone of the pine mushroom *Tricholoma matsutake*. J Microbiol 50:199–206. 10.1007/s12275-012-1371-7 22538647

[CR16] Khiralla A, Mohammed AO, Yagi S (2022) Fungal perylenequinones. Mycol Prog 21(3):38. 10.1007/s11557-022-01790-4 PMC8977438 35401071

[CR17] Krieg NR, Holt JG (1984) Bergey’s manual of systematic bacteriology, vol I. Williams and Wilkins, Baltimore

[CR18] Kumar S, Stecher G, Tamura K (2016) MEGA7: molecular evolutionary genetics analysis version 7.0 for bigger datasets. Mol Biol Evol 33(7):1870–1874. 10.1093/molbev/msw054 PMC8210823 27004904

[CR19] Law S, Lo C, Han J, Leung AW, Xu C (2021) Antimicrobial photodynamic therapy with hypocrellin B against SARS-CoV-2 infection? Photodiagn Photodyn Ther 34:102297. 10.1016/j.pdpdt.2021.102297 PMC8057054 33892181

[CR20] Lei XY, Zhang MY, Ma YJ, Wang JW (2017) Transcriptomic responses involved in enhanced production of hypocrellin A by addition of Triton X-100 in submerged cultures of *Shiraia bambusicola*. J Ind Microbiol Biotechnol 44:1415–1429. 10.1007/s10295-017-1965-5 28685359

[CR22] Li WZ, Godzik A (2006) Cd-hit: a fast program for clustering and comparing large sets of protein or nucleotide sequences. Bioinformatics 22:1658–1659. 10.1007/978-1-4899-7478-5_221 16731699

[CR21] Li Q, Chen C, Penttinen P, Xiong CH, Zheng L, Huang W (2016) Microbial diversity associated with *Tricholoma matsutake* fruiting bodies. Microbiology 85(5):531–539. 10.1134/S0026261716050106 26428733

[CR24] Li YT, Yang C, Wu Y, Lv JJ, Feng X, Tian XF, Zhou ZZ, Pan XY, Liu SW, Tian LW (2021) Axial chiral binaphthoquinone and perylenequinones from the stromata of *Hypocrella bambusae* are SARS-CoV–2 entry inhibitors. J Nat Prod 84(2):436–443. 10.1021/acs.jnatprod.0c01136 33560122

[CR23] Li XP, Shen WH, Wang JW, Zheng LP (2025) Production of fungal hypocrellin photosensitizers: exploiting bambusicolous fungi and elicitation strategies in mycelium cultures. Mycology 16(2):593–616. 10.1080/21501203.2024.2430726 PMC12096664 40415904

[CR26] Liu YX, Liu ZY, Yang YL, Wongkaew S (2009) Isolation, screening and confirmative identification of high hypocrellin a producing *Shiraia bambusicola* isolates. Khon Kaen Agric J 37:357–364. https://api.semanticscholar.org/CorpusID:199367847

[CR25] Liu B, Bao JY, Zhang ZB, Yan RM, Wang Y, Yang HL, Zhu D (2018) Enhanced production of perylenequinones in the endophytic fungus *Shiraia* sp. Slf14 by calcium/calmodulin signal transduction. Appl Microbiol Biotechnol 102:153–163. 10.1007/s00253-017-8602-0 29098415

[CR27] Lyu X, Lyu Y, Yu H, Chen W, Ye L, Yang R (2022) Biotechnological advances for improving natural pigment production: a state-of-the-art review. Bioresour Bioprocess 9:8. 10.1186/s40643-022-00497-4 PMC10992905 38647847

[CR28] Ma G, Khan SI, Jacob MR, Tekwani BL, Li Z, Pasco DS, Walker LA, Khan IA (2004) Antimicrobial and antileishmanial activities of hypocrellins A and B. Antimicrob Agents Chemother 48(11):4450–4452. 10.1128/AAC.48.11.4450-4452.2004 PMC525422 15504880

[CR30] Ma YJ, Lu CS, Wang JW (2018) Effects of 5-azacytidine on growth and hypocrellin production of *Shiraia bambusicola*. Front Microbiol 9:2508. 10.3389/fmicb.2018.02508 PMC6200910 30405568

[CR31] Ma YJ, Zheng LP, Wang JW (2019) Bacteria associated with *Shiraia* fruiting bodies influence fungal production of hypocrellin A. Front Microbiol 10:2023. 10.3389/fmicb.2019.02023 PMC6749022 31572311

[CR29] Ma YJ, Li XP, Li JH, Zheng LP, Wang JW (2025) Fruiting body-associated *Pseudomonas* contact triggers ROS-mediated perylenequinone biosynthesis in *Shiraia* mycelium culture. Bioresour Bioprocess 12(1):106. 10.1186/s40643-025-00946-w PMC12474784 41003957

[CR32] Magoč T, Salzberg SL (2011) FLASH: fast length adjustment of short reads to improve genome assemblies. Bioinformatics 27:2957–2963. https://link.cnki.net/doi/10.1093/bioinformatics/btr507 PMC3198573 21903629

[CR33] Morakotkarn D, Kawasaki H, Tanaka K, Okane I, Seki T (2008) Taxonomic characterization of *Shiraia*-like fungi isolated from bamboos in Japan. Mycoscience 49:258–265. 10.1007/s10267-008-0419-3

[CR34] Moree WJ, Phelan VV, Wu CH, Bandeira N, Cornett DS, Duggan BM, Dorrestein PC (2012) Interkingdom metabolic interactions configured by mass spectrometry imaging. Proc Natl Acad Sci USA 109(34):13811–13816. 10.1073/pnas.1206855109 PMC3427086 22869730

[CR35] Mulrooney CA, O’Brien EM, Morgan BJ, Kozlowski MC (2012) Perylenequinones: isolation, synthesis, and biological activity. Eur J Org Chem 21:3887–3904. 10.1002/ejoc.201200184 PMC3770481 24039544

[CR36] O’Brien EM, Morgan BJ, Mulrooney CA, Carroll PJ, Kozlowski MC (2010) Perylenequinone natural products: total synthesis of hypocrellin A. J Org Chem 75:57–68. 10.1021/jo901386d PMC2798931 19894741

[CR37] Oh SY, Kim M, Eimes JA, Lim YW (2018) Effect of fruiting body bacteria on the growth of *Tricholoma matsutake* and its related molds. PLoS ONE 13(2):e0190948. 10.1371/journal.pone.0190948 PMC5805168 29420560

[CR38] Partida-Martinez L, Hertweck C (2005) Pathogenic fungus harbours endosymbiotic bacteria for toxin production. Nature 437:884–888. 10.1038/nature03997 16208371

[CR39] Pent M, Põldmaa K, Bahram M (2017) Bacterial communities in boreal forest mushrooms are shaped both by soil parameters and host identity. Front Microbiol 8:836. 10.3389/fmicb.2017.00836 PMC5423949 28539921

[CR40] Sbrana C, Agnolucci M, Bedini S, Lepera A, Toffanin A, Giovannetti M, Nuti MP (2002) Diversity of culturable bacterial populations associated to *Tuber borchii* ectomycorrhizas and their activity on *T. borchii* mycelial growth. FEMS Microbiol Lett 211(2):195–201. 10.1111/j.1574-6968.2002.tb11224.x 12076812

[CR41] Scherlach K, Hertweck C (2009) Triggering cryptic natural product biosynthesis in microorganisms. Org Biomol Chem 7:1753–1760. 10.1039/b821578b 19590766

[CR42] Schloss PD, Westcott SL, Ryabin T, Hall JR, Hartmann M, Hollister EB, Lesniewski RA, Oakley BB, Parks DH, Robinson CJ, Sahl JW, Stres B, Thallinger G, Horn DJ, Weber CF (2009) Introducing mothur: open-source, platform-independent, community-supported software for describing and comparing microbial communities. Appl Environ Microbiol 75:7537–7541. 10.1128/AEM.01541-09 PMC2786419 19801464

[CR43] Selegato DM, Castro-Gamboa I (2023) Enhancing chemical and biological diversity by co-cultivation. Front Microbiol 14:1117559. 10.3389/fmicb.2023.1117559 PMC9928954 36819067

[CR44] Selim MSM, Abdelhamid SA, Mohamed SS (2021) Secondary metabolites and biodiversity of actinomycetes. J Genet Eng Biotechnol 19(1):72. 10.1186/s43141-021-00156-9 PMC8116480 33982192

[CR45] Shan W, Zhou Y, Liu H, Yu XJ (2018) Endophytic actinomycetes from tea plants (*Camellia sinensis*): isolation, abundance, antimicrobial, and plant-growth promoting activities. BioMed Res Int 2018:1470305. 10.1155/2018/1470305 PMC6241348 30519568

[CR46] Splivallo R, Deveau A, Valdez N, Kirchhoff N, Frey-Klett P, Karlovsky P (2015) Bacteria associated with truffle-fruiting bodies contribute to truffle aroma. Environ Microbiol 17(8):2647–2660. 10.1111/1462-2920.12521 24903279

[CR47] Stergiopoulos I, Zwiers LH, De Waard MA (2002) Secretion of natural and synthetic toxic compounds from filamentous fungi by membrane transporters of the ATP-binding cassette and major facilitator superfamily. Eur J Plant Pathol 108(7):719–734. 10.1007/978-94-010-0001-7_16

[CR48] Sun CX, Ma YJ, Wang JW (2017) Enhanced production of hypocrellin A by ultrasound stimulation in submerged cultures of S*hiraia bambusicola*. Ultrason Sonochem 38:214–224. 10.1016/j.ultsonch.2017.03.020 28633821

[CR49] Sun CX, Ma YJ, Wang JW (2018) Improved hypocrellin A production in *Shiraia bambusicola* by light-dark shift. Photochem Photobiol B 182:100–107. 10.1016/j.jphotobiol.2018.04.004 29656218

[CR50] Taechowisan T, Peberdy JF, Lumyong S (2003) Chitinase production by endophytic *Streptomyces aureofaciens* CMUAc130 and its antagonism against phytopathogenic fungi. Ann Microbiol 53(4):447–462

[CR51] Varese GC, Portinar S, Trott A, Scannerini S, Luppi-Mosca AM, Martinotti MG (1996) Bacteria associated with *Suillus grevillei* sporocarps and ectomycorrhizae and their effects on in vitro growth of the mycobiont. Symbiosis 21(2):129–147. 10.1016/0923-2508(96)80211-7

[CR52] Wang WJ, Li XP, Shen WH, Huang QY, Cong RP, Zheng LP, Wang JW (2024) Nitric oxide mediates red light-induced perylenequinone production in *Shiraia* mycelium culture. Bioresour Bioprocess 11:2. 10.1186/s40643-023-00725-5 PMC10991179 38647587

[CR53] Wen YD, Liao BS, Yan XX, Wu ZQ, Tian XF (2022) Temperature-responsive regulation of the fermentation of hypocrellin A by *Shiraia bambusicola* (GDMCC 60438). Microb Cell Fact 21(1):135. 10.1186/s12934-022-01862-w PMC9254528 35787717

[CR54] Wu LY, Wen CQ, Qin YJ, Yin HQ, Tu QC, Nostrand JDV, Yuan T, Yuan M, Deng Y, Zhou J (2015) Phasing amplicon sequencing on Illumina Miseq for robust environmental microbial community analysis. BMC Microbiol 15:125. 10.1186/s12866-015-0450-4 PMC4472414 26084274

[CR55] Xiang QJ, Luo LH, Liang YH, Chen Q, Zhang XP, Gu YF (2017) The Diversity, growth promoting abilities and anti-microbial activities of bacteria isolated from the fruiting body of *Agaricus bisporus*. Pol J Microbiol 66(2):201–207. 10.5604/01.3001.0010.7837 28735315

[CR56] Xing MZ, Zhang XZ, Sun ZL, Zhang HY (2003) Perylenequinones act as widespectrum fungicides by generating reactive oxygen species both in dark and in light. J Agric Food Chem 51:7722–7724. 10.1021/jf0348774 14664535

[CR57] Yang H, Xiao C, Ma W, He G (2009) The production of hypocrellin colorants by submerged cultivation of the medicinal fungus *Shiraia bambusicola*. Dyes Pigm 82:142–146. 10.1016/j.dyepig.2008.12.012

[CR58] Zhang X, Wei QL, Tian LW, Huang ZX, Tang YB, Wen YD, Yu FQ, Yan XX, Zhao YC, Wu ZQ, Tian XF (2024) Advancements and future prospects in hypocrellins production and modification for photodynamic therapy. Fermentation 10(11):559. 10.3390/fermentation10110559

[CR59] Zheng LP, Ji HY, Zhou JQ, Wang JW (2025) *Shiraia* fruiting body-associated fungi regulate the biosynthesis of hypocrellin A. Acta Microbiol Sin 65(4):1758–1773 (in Chinese). 10.13343/j.cnki.wsxb.20250009

