# Supplementary material for: Exploring endophytic actinomycetes from Shiraia fruiting bodies to enhance fungal hypocrellin A biosynthesis
Source: Bioresour Bioprocess. 2026 Oct 29;13(1):161. doi: 10.1186/s40643-026-01154-w (PMC13719979; doi:10.1186/s40643-026-01154-w)
Supplement: Supplementary file 1 [file 40643_2026_1154_MOESM1_ESM.pdf]

Supplementary materials

Exploring endophytic actinomycetes from *Shiraia* fruiting bodies to enhance fungal hypocrellin A biosynthesis

Wen Hao Shen, Li Ping Zheng, Jian Qin Zhou, and Jian Wen Wang

✉ Jian Wen Wang jwwang@suda.edu.cn

Table S1. Composition of the media for isolation of endophytic actinomycetes from *Shiraia* fruiting bodies.

Table S2. The primers and nucleotide sequences of some HA biosynthetic genes for qRT-PCR.

Table S3. 16S rDNA gene sequence analysis of actinomycetes isolated from *Shiraia* fruiting body based on BLAST analysis in the NCBI database.

Table S4. Taxonomic classification of 28 actinomycetes isolates from *Shiraia* fruiting bodies.

Table S5. Number of bacterial sequences by high-throughput sequencing in *Shiraia* fruiting body.

Table S6. Taxonomic classification of OTUs detected from the fruiting bodies with read numbers of the fruiting body-associated actinomycetes.

Table S7. Diversity index of coral symbiotic actinomyces 16S rDNA gene library.

Table S8. The OTU taxonomy of actinomycetes community in *Shiraia* fruiting bodies.

Table S9. Taxonomic classification of OTUs detected from the fruiting bodies with read numbers of the fruiting body-associated actinomycetes.

Table S10. The physiological and biochemical characteristics of *S. pseudogriseolus* A6.

Table S11. Secondary metabolite biosynthesis gene clusters of *S. pseudogriseolus* A6.

Fig. S1. Prediction of secondary metabolite gene clusters of *S. pseudogriseolus* A6.

Fig. S2. Effects of introducing time of live *S. pseudogriseolus* A6 on fungal biomass (**A**), hypocrellin A (HA) content in mycelium (**B**), the released HA in cultural broth (**C**) and total HA production (**D**) in submerged culture of *Shiraia* sp. S9. Total HA production refers to the sum of the intracellular and extracellular HA. Live A6 (5.0×10^6 CFU/mL) was added on different time points and incubated at 150 rpm and 28℃. The mycelia were harvested on day 8. The S9 strain treated with the equal volume of sterile TSB broth instead of live actinobacterial suspension was used as control group. Values are mean ± SD from three independent experiments ($^{*}P < 0.05$, $^{**}P < 0.01$ versus control group).

Fig. S3. Effects of different concentrations of live *S. pseudogriseolus* A6 on fungal biomass (**A**), HA content in mycelium (**B**), the released HA in cultural broth (**C**) and total HA production (**D**) in submerged culture of *Shiraia* sp. S9. Total HA production refers to the sum of the intracellular and extracellular HA. Live A6 was added on day 5 of the culture at different volumes at 150 rpm and 28℃. The mycelia were harvested on day 8. The S9 strain treated with the equal volume of sterile TSB broth instead of live actinobacterial suspension was used as control group. Values are mean ± SD from three independent experiments ($^{*}P < 0.05$, $^{**}P < 0.01$ versus control group).

Table S1 Composition of the media for isolation of endophytic actinomycetes and co-culture

Medium	Medium formula
Humic acid-vitamin (HV) agar	Humic acid 1 g， Na_2HPO_4 0.5 g， KCl 1.7 g， $MgSO_4·7H_2O$ 0.05 g， $FeSO_4·7H_2O$ 0.01 g， $CaCO_3$ 0.02 g， agar 20 g， H_2O 1 L
Sodium succinate-asparagine agar medium (SSA）	Sodium succinate 1 g， L-asparagine 0.2 g， KH_2PO_4 0.9 g， K_2HPO_4 0.6 g， $MgSO_4·7H_2O$ 0.1 g， $CaCl_2·2H_2O$ 0.2 g， KCl 0.3 g， $FeSO_4·7H_2O$ 0.001 g， agar 15.0 g， H_2O 1 L
Dulcitol-proline medium (DP)	Dulcitol 2.0 g， proline 0.5 g， K_2HPO_4 0.3 g， NaCl 0.3 g， $MgSO_4·7H_2O$ 1.0 g， $CaCl_2·2H_2O$ 1.0 g， agar 20.0 g， H_2O 1 L
Sodium propionate-asparagine (SPA) agar	Sodium propionate 2 g， L-asparagine 1.0 g， NH_4NO_3 0.1 g， KCl 0.1 g， $MgSO_4$ $·7H_2O$ 0.05 g， $FeSO_4.7H_2O$ 0.05 g， NaCl 30 g， agar 20 g， H_2O 1 L，
Gause's synthetic medium No.1 (GM1)	Starch 20 g， KNO_3 0.1 g， K_2HPO_4 0.05 g， $MgSO_4$ $7H_2O$ 0.05 g， NaCl 0.05 g， $FeSO_4.7H_2O$ 0.001 g， agar 20 g， H_2O 1 L
Tap water yeast extract (TWYE)	Yeast extract 0.25 g， KH_2PO_4 0.5 g， H_2O 1 L， agar 20 g
Potato dextrose agar (PDA) medium	Potato extract 4.0 g, dextrose 20.0 g, agar 15.0, H_2O 1 L

Table S2 The primers and nucleotide sequences of some HA biosynthetic genes for qRT-PCR

Target gene	Primer sequence	Gene description	Gene sequence
18S	F: 5'-ACGCAGCGAAATGCGATAAG-3'<br>R: 5'-CAAATTGTGCTGCGCTCCAA-3'	18S rRNA, *Shiraia* sp. SUPER-H168 [EU267792.1]	GTAGTCATATGCTTGTCTCAAAGATTAAGCCATGCATGTCTAAGTATAAGCAATTATACCGTGAAACTGCGAATGG<br>CTCATTAAATCAGTTATCGTTTATTTGATAGTACCTTACTACTTGGATAACCGTGGTAATTCTAGAGCTAATACATGC<br>TTACAACCCCGACTTCGGAAGGGGTGTATTTATTAGATAAAAAACCAACGCCCTTCGGGGCTTCTTGGTGATTCAT<br>GATAACTTTACGGATCGCATGGCCTTGCGCCGGCGACGGTTCATTCAAATTTCTGCCCTATCAACTTTCGATGGTA<br>AGGTATTGGCTTACCATGGTTTCAACGGGTAACGGGGAATTAGGGTTCGATTCCGGAGAGGGAGCCTGAGAAAC<br>GGCTACCACATCCAAGGAAGGCAGCAGGCGCGCAAATTACCCAATCCCAATACGGGGAGGTAGTGACAATAAAT<br>ACTGATACAGGGCTCTTTTGGGTCTTGTAATTGGAATGAGTACAATTTAAACCTCTTAACGAGGAACAATTGGAG<br>GGCAAGTCTGGTGCCAGCAGCCGCGGTAATTCCAGCTCCAATAGCGTATATTAAAGTTGTTGCAGTTAAAAAGCT<br>CGTAGTTGAACCTTGGGTCTGGCTGGCAGGTCCGCCTCACCGCGTGTACTTGTCCGGCCGGGCCTTCCTTCTGGA<br>GAACCTCATGCCCTTCACTGGGCGTGTTGGGGACCAGGACTTTTACTTTGAAAAAATTAGAGTGTTCAAAGCAG<br>GCCTTTGCTCGAATACGTTAGCATGGAATAATAGAATAGGACGTGCGGTCCTATTTTGTTGGTTTCTAGGACCGCC<br>GTAATGATTAATAGGGACAGTCGGGGGCATCAGTATTCAATTGTCAGAGGTGAAATTCTTGGATTTATTGAAGACT<br>AACTACTGCGAAAGCATTTGCCAAGGATGTTTTCATTAATCAGTGAACGAAAGTTAGGGGATCGAAGACGATCAG<br>ATACCGTCGTAGTCTTAACCGTAAACTATGCCGACTAGGGATCGGGCGATGTTATTTTTCTGACTCGCTCGGCACC<br>TTACGAGAAATCAAAGTTTTTGGGTTCTGGGGGGAGTATGGTCGCAAGGCTGAAACTTAAAGAAATTGACGGAA<br>GGGCACCACCAGGCGTGGAGCCTGCGGCTTAATTTGACTCAACACGGGGAAACTCACCAGGTCCAGATGAAATA<br>AGGATTGACAGATTGAGAGCTCTTTCTTGATTTTTCAGGTGGTGGTGCATGGCCGTTCTTAGTTGGTGGAGTGATT<br>TGTCTGCTTAATTGCGATAACGAACGAGACCTTAACCTGCTAAATAGCCAGGCTAGCTTTGGCTGGTCGCCGGCT<br>TCTTAGAGGGACTATCGGCTCAAGCCGATGGAAGTTTGAGGCAATAACAGGTCTGTGATGCCCTTAGATGTTCTG<br>GGCCGCACGCGCGCTACACTGACAGAGCCAACGAGTTCTTCACCTTGGCCGGAAGGTCTGGGTAATCTTGTTAA<br>ACTCTGTCGTGCTGGGGATAGAGCATTGCAATTATTGCTCTTCAACGAGGAATGCCTAGTAAGCGCGTGTCATCA<br>GCATGCGTTGATTACGTCCCTGCCCTTTGTACACACCGCCCGTCGCTACTACCGATTGAATGGCTCAGTGAGGCGT<br>CCGGACTGGCTTAGAGAAGTTGGCAACGACTACTTTGAGTTGGAAAGTTCGTCAAACTCGGTCATTTAGAGGAA<br>GTAAAAGTCGTAACAAGGTTTCCGTAGGTGAACCTGCGGA

MFS	F: 5’-TCCATTGTTCCAGGCGTACC-3’<br>R: 5’-TATGCCTCGCCGTTCTTTGT-3’	Major facilitator superfamily transporter, *Shiraia* sp. slf14 [AIW00660.1]	ATGCTGCATTTTGGCCTGTTTGCGAACCATTTTGGCGTGGTGTTTACCGCGCTGCGCCTGAGCATTGGCACCCGC<br>GATTGCTTTGCGCGCCGCGGCCCGCGCCCGAGCGATACCAACAACTTTCCGGGCTGCCAGGGCAGCCGCAAAGC<br>GCTGTGCGAAAGCCTGCCGCTGCCGAGCGTGGGCAACAGCCAGATTGCGGATACCACCAGCACCAACCAGAGC<br>TTTACCATGGCGGCGAGCATTAGCGGCGATACCACCGGCAGCGTGCAGGTGCCGGCGACCAGCCAGTGGAAATT<br>TGCGGCGCTGTTTTTTGGCCTGTGCCTGGCGGTGCTGTGCCAGGCGCTGGATAACACCATTCTGGCGACCGCGAT<br>TCCGAAAATTACCGATGAATTTAACAGCCTGAGCGATATTGGCTGGTATGGCAGCGGCTATCTGCTGACCACCTGC<br>GCGTTTCAGCTGAGCTATGGCAAACTGTATAACCTGTTTCCGATTAAATGGGTGTTTCTGATTGCGCTGGGCCTGT<br>TTGAACTGGGCAGCCTGGTGTGCGGCGCGGCGCCGACCAGCGTGGGCCTGATTATGGGCCGCGTGGTGGCGGGC<br>ATTGGCAGCGGCGGCATTTTTAGCGGCGCGACCCTGCTGGTGGCGGAACTGAAACCGCTGAAAGAACGCCCGAT<br>TTATAACGGCATTCTGGGCGCGATGTATGCGATTGCGAGCGTGGCGGGCCCGCTGATGGGCGGCGCGTTTACCGA<br>TCATGTGACCTGGCGCCTGTGCTTTTATATTAACCTGCCGCTGGGCCTGGTGACCGCGGTGATTGTGTTTTTTGTG<br>GTGCCGAGCGCGTATGATCCGGAACGCCATAGCAAACAGAAACTGACCCTGAAAAACAAACTGCATGAAATGGA<br>TCTGTATGGCCTGGCGGCGCTGGTGCCGGCGATTGTGTGCATTCTGCTGGCGGTGCAGTGGGGCGGCGCGCGCTA<br>TGCGTGGAACAACGGCCGCATTATTGCGCTGTTTGTGCTGGGCGGCATTCTGGCGATTGTGTTTGTGCTGATTGA<br>ACGCAAACAGGGCGATCGCGCGATTGTGCCGCCGAGCGTGATTAAACGCCGCACCGTGTGGGCGTGCAGCTTTT<br>ATAGCGCGATGCTGTTTGGCAGCTTTCTGGCGCTGTGCTATTATCTGCCGATTTGGTTTCAGGCGATTAAACGCGA<br>TAGCGCGACCCAGAGCGGCATTCATAACCTGCCGAGCATTCTGGGCACCACCATTTTTAGCATTGTGGCGGGCGG<br>CCTGGTGTTTGCGGTGGGCTATTATACCTGGGCGTGCATTCTGGCGAGCGCGATGGCGGCGGTGGGCGGCGGCCT<br>GCTGAGCACCCTGCAGGTGGATAGCAGCAGCGCGAAATGGATTGGCTATCAGATTATTTATGGCGCGGGCGTGGG<br>CTTTGGCCTGAACCAGCCGCTGATTGCGGTGCAGACCGCGCTGCCGAACCATCAGATTGCGGAAGGCACCGCGG<br>TGATTATTTTTATGCAGACCTTTGGCGGCACCATTTTTATTGCGGTGGCGCAGAACGTGTTTAACAACAAACTGAT<br>TGCGAACGTGCGCGCGCAGGCGATTCCGGTGGATCCGGTGGCGCTGCTGAGCGTGGGCGCGACCCAGCTGCAG<br>AAACTGGTGGATCCGCAGTATTTTGGCCGCCTGCAGCTGGCGTATAACGAAAGCATTACCCAGACCTTTTATGTG<br>GCGGTGGCGAGCGCGGGCCTGAGCATTATTGGCAGCGCGCTGATTCCGTGGTTTAGCGTGAAAAAAAAACCGGT<br>GGATAGC

PKS	F: 5’-GCTGTCCTGAAACGACTGGA-3’<br>R: 5’-CGAATATCGGGCACGTCTGA-3’	Polyketide synthase, *Shiraia* sp. slf14 [AIW00658.1]	ATGGCGGAACCGATTAAAGTGTTTGTGTTTGGCGATCAGACCTATGATGCGAGCGATCTGATGTTTACCCTGCTGC<br>ATGCGAACGATGATGTGATTCTGACCAGCTTTCTGGATAGCAGCCTGCAGACCCTGAAACGCGAAATTACCAAAC<br>TGAGCAACCAGCAGCGCGCGGCGTGCCCGCGCTTTAGCAAACTGGCGGAACTGCTGCCGTATTGGCGCAAAGG<br>CAGCCTGAACCCGGCGCTGGATCAGGCGCTGACCTGCATTTGCCATATTGGCACCTTTATTCATGAACATACCAGC<br>GCGGGCAAAAGCTTTCCGACCAGCCAGGATGCGTGCCTGACCGCGCGCTGCACCGGCTTTCTGAGCGCGGTGGC<br>GGTGAGCTGCAGCAGCAACGCGGTGCAGCTGCTGCAGCTGGGCCTGGAAACCGTGGCGGTGGCGTTTCGCGTG<br>GGCGCGTGCGCGTGGGAACTGGGCAGCCTGATTAGCAGCAGCGCGGATGATACCCTGCCGTATCCGAGCTGGAC<br>CATTGCGCTGGCGGGCTTTCGCGAAGAACAGGTGAGCGAATGCATTGATGATTATGTGAGCAAAAACGCGCTGA<br>GCGTGGTGGCGCGCCCGTATATTAGCGCGCGCATTAGCAGCAGCCGCTGCAGCATTAGCGCGCCGCCGGATGTGC<br>TGCATGAACTGCTGACCACCACCGAACTGAGCGGCGCGACCAAAATTGAACTGCCGATTACCGCGCCGTATCAT<br>GCGCCGCATCTGTATAGCACCAGCGATGTGGATGGCATTGTGAACGATGTGAACCAGATTAGCAGCGTGACCCGC<br>ATTCCGATTATTAGCAGCAGCAGCGGCGCGAGCATTAAAGATATTGAATTTCGCGATGCGCTGCGCATTAGCGTGG<br>AAGCGTGCCTGCAGCAGACCATTCGCAGCGATCTGGTGGCGGCGGGCGTGGCGGCGCATCTGCAGGCGACCAG<br>CCTGTTTGCGATTCATCATATTGCGACCGCGCCGGATGGCCTGGGCAGCGCGGTGCAGGCGGCGCTGGGCCCGA<br>GCAGCAAACAGATTGATCTGAGCGAACAGCATGGCAGCAACATTCCGAAAAGCCTGGGCCTGCCGCTGGCGAA<br>AAGCAAAATTGCGATTCTGAGCATGAGCGGCCGCTTTCCGCAGGCGCCGAGCATGGATGCGTTTTGGCATGTGCT<br>GAAAAACGGCATTGATACCCATGAACTGGCGCTGGCGAGCCGCTGGAACACCCGCACCCATGTGGGCGATGTGA<br>GCCTGAAAAGCCTGCCGAAAAACACCAGCGGCACCGGCTTTGGCTGCTGGCTGCATGATGCGGCGCAGTTTGAT<br>GCGCGCTATTTTAACATGAGCCCGCGCGAAGCGCCGCAGGTGGATCCGGCGCAGCGCATTGCGCTGCTGACCGC<br>GACCGAAGCGCTGGAACAGGCGGGCATTGTGCCGGGCCGCACCAGCAGCACCCAGAAAGATCGCGTGGGCGTG<br>TATTTTGGCAGCACCAGCAACGATTGGATGGAAACCAACAGCGCGCAGGATATTGATACCTATTTTATTCCGGGCG<br>GCAACCGCGCGTTTATTCCGGGCCGCATTAACTATCATTTTAAATTTAGCGGCCCGAGCTATACCATTGATACCGCG<br>TGCAGCAGCAGCTTTACCGCGATTCATCTGGCGTGCAACGCGCTGTGGAAAGGCGAAGTGGATACCGCGATTGT<br>GGGCGGCACCAACGTGCTGACCAACCCGGATATGACCGCGGGCCTGGATCGCGGCCATTTTCTGAGCCGCACCG<br>GCAACTGCAAAACCTTTGATGATACCGCGGATGGCTATTGCCGCGGCGAAGCGGTGGCGACCGCGGTGCTGAAA<br>CGCCTGGAAGATGCGATTGCGGATAAAGATCCGGTGCAGGCGTGCATTCTGAGCATTGGCACCAACCATAGCGCG

	GAAGCGGATAGCATTACCCGCCCGCATGTGGGCGCGCAGCAGGCGCTGTTTGGCAACGTGCTGGCGGATGCGGG<br>CGTGAAACTGACCGATATTAGCTATTGCGAAATGCATGGCACCGGCACCCAGGCGGGCGATGCGGGCGAAACCA<br>TGAGCGTGCTGCAGAGCCTGGCGCCGAGCGAACCGGCGAAAGCGGTGCGCAAACCGCATCAGCGCCTGTATATT<br>GGCGCGGCGAAAAGCAACGTGGGCCATGGCGAAGCGGCGGCGGGCGTGACCAGCCTGGCGAAAGTGCTGCTG<br>ATGCTGAAAAACAGCACCATTCCGCCGCATTGCGGCATTAAAACCAAAATTAACCATAAACTGCCGGATCTGGAT<br>GCGCGCAACACCAGCATTGCGATGACCCCGGTGCCGTGGAACCGCCCGACCAACGGCAAACGCCAGGTGCTGC<br>TGAACAACTTTAGCGCGGCGGGCGGCAACACCGCGCTGGTGCTGGAAGATGCGCCGGAAATTCTGACCAGCGAT<br>GTGCCGGATGTGCGCAGCAAACATCTGGTGGCGGTGAGCGCGAAAACCCCGACCAGCCTGGAAAACAACCTGC<br>GCAACCTGCTGGAATGGACCAAAGCGCAGAAAAGCAGCGATAACCTGACCCTGGCGCGCCTGAGCTATACCACC<br>ACCGCGCGCCGCATGCATCATCCGCATCGCGTGATTGTGAGCGCGAGCGATCTGCAGAGCGTGGAAAGCCTGCT<br>GCAGAAAGCGCTGGATCAGAAAGATGGCAGCAGCCGCTTTAAAGGCGCGCCGCGCTATGTGTTTGCGTTTACCG<br>GCCAGGGCGCGCAGTTTGCGGGCATGGGCGTGGATCTGTTTGCGCGCCTGAAAGCGTTTCGCGCGGATATTTGCC<br>GCTATGATCAGATTTGCAAACAGCTGAACCTGCCGAGCATTCAGACCCTGTTTGAAGATGAAAGCAAATTTAGCA<br>ACGCGAGCCCGACCATGCTGCAGCTGGCGAGCGTGTGCTTTCAGATGGCGCTGTATCGCATGTGGCTGAGCCTG<br>GGCATTAGCCCGAGCGCGGTGGTGGGCCATAGCCTGGGCGAATATCCGGCGCTGTATGCGGCGAGCGTGCTGAG<br>CCAGGCGGATGTGATTAACCTGGTGGGCCAGCGCGCGCTGCTGCTGGAAAAACATTGCGAACTGGGCAGCCATG<br>TGATGCTGGCGGTGCGCAGCAGCGTGAGCGATCTGGAAGCGGCGCTGGGCCGCCCGGGCACCGCGTATGAAATT<br>AGCTGCCATAACGGCCGCGAAAGCGTGGTGCTGGGCGGCACCAAAGCGCAGATTGAACAGATTCGCCCGAGCC<br>TGCAGCGCAGCCGCACCAGCCATAAACTGCTGGAAGTGCCGTATGCGTATCATACCAGCCAGGTGGAAGCGATTC<br>TGGAACCGCTGCAGGCGGTGGCGAAAGGCATTCGCTTTGCGAAACCGGCGATTCCGGTGATTAGCCCGACCTAT<br>GGCAAAGTGCTGACCGAACTGGGCGATATTAGCAGCGATTTTATTGTGCGCCATTGCCGCAACAAAGTGGATATG<br>CTGAGCGCGCTGAACGCGGCGAAAACCATTGGCCTGCTGGATGAAAAAATGATGGGCATTGAAATTGGCCCGGA<br>TCCGGTGGTGGTGAAAATGGTGAAAGAAGTGGCGGGCCCGACCTTTCGCACCTTTGCGAGCGTGCGCAAAGGC<br>GAAGATACCAGCGCGGTGCTGGCGAGCGCGTTTAGCGCGTTTTATACCGCGGGCGCGGATATTAACTGGATGACC<br>TATCATAGCGATTTTGCGAGCTGCCAGGTGGTGCTGGATCTGCCGGCGTATGCGTGGGATCTGAAAGATTATTGGA<br>TTCAGTATGTGCATGATTGGAGCCTGCGCAAAGGCGATCCGCCGCTGATTCTGGAACCGACCAGCCTGGAAGGC

	ACCTGCATTCATAAAGTGCTGGAAAACAGCCTGAACGTGAGCGGCGGCGAACTGCTGGTGGAAGCGGATCTGA<br>GCCGCGAAGATCTGCATCCGATGGTGCAGGGCCATAAAGTGTATGGCGTGCCGCTGTGCACCCCGAGCGTGTATG<br>CGGATATTGCGCTGACCATTGGCGAATATGCGAAACGCTGCCTGGAACCGGGCCAGGCGACCATTGGCATTGAAG<br>TGGCGAACATGAACATTCAGAGCGCGCTGGTGGCGAACACCGATGGCCAGACCCAGCTGCTGCAGACCAAAGC<br>GCGCTTTGATAGCGAAGATAAAAGCCTGTTTTGCACCTTTAGCAGCGTGGAAAAAGATGGCAAAGTGAAAGAAC<br>AGCATAGCAACTGCCTGATTCGCCTGTTTAACGTGGAAGAAGCGCGCAACACCCTGGTGAAAAACACCCCGCAG<br>GTGAAAAGCCGCATTGCGACCCTGCAGGGCCAGCTGCAGGATTTTGGCAACACCTTTCGCTTTAGCAAAAGCAT<br>GATTTATAAAATGGTGGGCCAGCTGGCGGATTTTGATCCGAAATATCGCGCGCTGGAAGAAATTACCCTGGATAGC<br>GATAACCTGGAAGCGACCGGCCGCGTGAACTTTGCGAAAGTGAAAACCGATGGCAAATTTCATACCAACCCGGC<br>GTATATTGATGCGCTGAGCCAGCTGGGCGGCTTTGTGATGAACGGCAACGAAGGCGTGGATCTGGATAAAGAAC<br>TGTTTGTGAACCATGGCTGGGAAAGCCTGAAACTGCTGGAACCGGTGGATCCGACCAAAATTTATAACACCCATG<br>TGAAAATGATTGAAGGCAAAGATAAACTGTGGACCGGCGATATTACCATTCTGGATGGCGATCAGATTGTGGGCA<br>TGTTTGGCGGCGTGGCGCTGCAGGGCGTGCCGAAACGCCTGATGAGCTATATTGTGAACGCGGCGAGCAAACGC<br>GCGAACGGCCCGAGCGTGAGCAAACCGGCGGAAATTGCGGCGAAACCGCCGGCGAGCGTGCCGGCGGTGAAC<br>ACCGTGGTGAAAACCGTGACCAAAAAAAGCACCGTGGCGCAGCCGCAGAGCAACGATCGCAGCATTGTGACCG<br>CGGTGAGCATTATTAGCCAGGAAAGCGGCATTGCGCTGAGCGAACTGACCGATGATACCCGCTTTGAAGATATTG<br>GCGTGGATAGCCTGCTGGGCCTGATGGTGAGCAGCCGCATGCGCGATGAACTGGGCATTGATTTTGATAGCAGCG<br>CGTTTCTGGAAATTCGCACCGTGGGCAGCTTTAAAACCTTTCTGAAAGGCCTGACCGGCAGCAACGAAATTACC<br>ACCACCACCGAAACCGTGCAGGAAGAACTGGCGAGCACCCTGGTGGAACCGGCGTTTTTTAACAACGATGCGA<br>AAGCGACCAACACCATTTGGAGCAAAATTCTGGATATGATTGCGGAAGAAAGCGGCATTAAAGTGGAAGAACTG<br>ACCGGCGATACCAACTTTAGCGATATTGGCGTGGATAGCCTGCTGAGCCTGGTGGTGGTGAGCCGCATGCGCGAT<br>GAACTGGAACTGGAAGTGAGCGAACAGAGCCTGTTTCTGGATTTTCCGACCGTGGAAAGCCTGAAAGCGCGCA<br>TTACCGGCGCGCGCCAGAGCCCGCCGACCGAAAGCGAAGCGAGCGATAGCGATCTGAGCACCGAAGTGAGCAG<br>CGTGTTTACCTATGAAGGCGTGATTGAAACCCCGATGGAAGAACTGTTTACCCCGGATAAAAAAGAAAGCGTGAT<br>TAGCATGACCGAAATTATTCCGCCGGTGAAACCGGCGTGGAGCATTATTCTGCAGGGCAGCCCGCGCAACGCGC<br>GCGAAAAACTGTTTCTGTTTCCGGATGGCTGCGGCGCGGCGACCAGCTATCTGAAACTGCCGGCGCTGAGCCCG

	AGCACCGCGGTGATTGCGTTTAACAGCCCGTTTATGAAAAACCCGCATGAAATGAACAGCCGCCAGCTGAGCGA<br>TATTGTGGCGAGCTATATTGAAAGCGTGCGCCGCCAGCAGCCGCATGGCCCGTATCATCTGGCGGGCTGGAGCGC<br>GGGCGGCATTCTGGCGTATGCGGTGGCGCAGGAACTGATGGCGACCGGCGATGAAATTGCGACCCTGACCCTGA<br>TTGATAGCCCGGCGCCGACCCATGGCCTGGATCGCCTGCCGAAACGCTTTTTTGATCATTGCAGCGCGGTGGGCA<br>TTTTTGGCGAAGAACTGAGCGCGATTAACCCGAGCAAAGAACGCGCGAAAATTCCGGATTGGCTGATGCCGCAT<br>TTTGAAGCGACCATTGAACTGCTGCATGATTATCATGCGCCGCCGATGCCGGCGGGCAGCCGCAGCCCGGGCAA<br>AGTGAGCATTATTTGGGCGGGCGCGTGCGCGTTTGGCGGCAAATATGCGCCGCTGGCGCCGGCGAGCATGGAAG<br>GCGAAGATACCGAAGGCATGAAATTTCTGACCGAACAGCGCACCGATTTTGGCCCGGGCGATTGGGCGAGCCTG<br>TTTCCGGGCAAAGAAATTACCGTGGATGTGATTAACGGCGAACATCATTTTAGCATGATGCGCGGCAAAGGCGGC<br>GATCAGCTGGGCCAGCTGATGCGCAAAGCGCTGGGCCTGGTGGTG

Mono	F: 5’-GGACGATTCCACGCGATTTG-3’<br>R: 5’-CATGGCTGACCGCATCATTG-3’	Monooxygenase (hydroxylase), *Shiraia* sp. slf14 [AIW00664.1]	ATGGCGATTAGCAGCCAGAACGATAAACTGATTGTGGCGATTTATGGCGGCGGCGTGACCTGCGCGACCCTGAGC<br>CAGGCGCTGAAAGATCAGATTCATCTGGATGTGCAGTATTTTGATCCGAACCCGGATCCGACCAGCGTGACCGTG<br>CCGTTTCTGAGCCTGTATCCGCAGGCGCATGATGCGCTGGCGTTTATTGGCAAAGAAGCGGGCGCGACCGTGGA<br>AAAAGTGGGCTGGTTTGCGGAAGCGCCGAGCCTGGTGGTGATGGGCCAGGGCAGCGATGCGGGCACCACCGTG<br>TGCGATCTGGCGGGCTTTCCGCGCAGCAGCAGCTGGCCGGAAGTGGTGGTGGTGCAGCCGGCGGCGTTTCTGAA<br>AGAAATGCTGGGCGGCCTGGTGCCGGAAGCGCTGCATACCAACAAAAAACTGGTGAGCGTGACCCATAGCGATC<br>GCAGCGCGCATCCGCTGGATATTCATTTTGAAGATGGCAGCACCCATCATGTGGATGTGCTGATTGGCGATGATGG<br>CCCGATGGGCGTGATGCGCAGCCTGGTGATTGGCAGCGATCATAGCGCGACCAAACCGGTGTTTGAAGGCTATCT<br>GACCGCGGTGTGCATGGTGCCGCCGGAAGAAAACATTGCGCAGTATCTGGATCCGAGCTATGCGGCGAAAGATG<br>CGGGCCGCCGCTTTGAACGCATTGGCAACAAAAGCTGGGTGAACAACGAAAACAGCCTGCGCGCGATTCTGGG<br>CACCTTTTATACCGATGAAGATTATAACGTGAGCCAGCCGCTGCGCAGCACCACCAGCGAAGAACTGCAGGAAC<br>ATTTTAAAGATTTTGAAGGCGGCGCGGGCATTACCGATGCGCTGACCAAATTTAGCGGCCTGCGCCTGATTCCGG<br>AAATTGCGCATCCGCATGCGCCGACCTATATTAACGGCCTGGTGGCGATGGCGGGCAACGCGGCGCATAAAATGA<br>CCAACTTTCAGCAGCTGCATTTTGGCCAGCAGATTGAAGATGCGATGATTCTGGGCGCGCTGCTGCGCGAAGCG<br>AAAAACCTGCATGGCGTGGCGGCGGCGCTGTATGCGTATGATGTGGTGCGCCGCCCGCAGAGCCAGTGGGTGAG<br>CGATCAGGCGAAATATCTGGGCGAACTGTGGCATGGCATGGCGGATGGCGTGGGCCTGGATGTGGCGCGCCTGA<br>AAGCGGCGTTTGAAAAATGGTGGAAAGAATGGCGCGAATATGATGTGAAAGAACATCGCCAGGATGCGCTGCGC<br>CTGATGCGCGAACGCCTGTGGGCGGCGGGCTATGTGGAAAGCCGCGAA

ABC	F: 5’-CATGTCTCCCGACCTCATCG-3’<br>R: 5’-AAGCGCAGTGTTTCGTTGAC-3’	ATP-binding cassette transporter, *S. bambusicola* S8*	GCTGCCTGCCCCTTGCCTGACCTTGTTTGTCTTTCAAGTCAGCCACTATACTACCATTTAATATCTTATTCTTCTT<br>TATTCTATCCTTTCATCAATAGACTAGCACCAGGATCACAGAGGCGACTCTTGCTCGCCAGCATGAGTTTAGTT<br>GGCAATATCCCATCAAATCACGACCGAGGGGTTATATCCGAGGGTGTTCCTGGCCCAGAAATGGCAGCCAGAA<br>GGCCATCACAGTATGAGGCTGAAGAGCGACAACATACTGCTGCTTCAGAATCAGATGCCTCGACAATTGCAGC<br>TAATGCTGAAAGCACATCGCAAATTCAACGCCCTCATTTGAAAGAATCTCAGCGCAGACAGAGTGTCGCATCC<br>ATTGCAGATGTTGAAGAGACTGCAAGAAGAGAACGACAAGTTCTCCACCTGGCTCGACGCCTGACAGCACAAA<br>GCCAGGGCGAACTAGGAAACCAAAATCCATTCAATGCACCCCATAACTCAGAACTAGACCCCAATGGCGACAA<br>CTTCAATGCCCGAGCATGGACCAAGGCTATGCTTCACTTGCAGCAAGAGGATGCACATGCTGCGCCAACCCGC<br>ACAGCTGGTGTGTCGTTCAAGAATCTCAACGTCCATGGCTTTGGTACCGACGCAGATTATCAGAAGAGTGTTGG<br>AAACGTGTGGCTTGAAGGCCCTGCCTTGGCGAAGCAGTTGATGGGTAACAAGGGCCGCAAAATTGATATCTTG<br>CAAAATCTGGATGGTTTGGTCGAGGCCGGTGAGATGCTGGTGGTTCTTGGTAAGTGCACTAATGTAGGACTTGT<br>GAAACGTAGATTAACTTTAGTATAGGACCCCCCGGATCTGGATGCTCAACTTTCTTGAAAACCATCACTGGGGA<br>AACTCATGGGCTTCTTCGTCCATGAAGACTCCCAGATCAATTATCAGGGTGTTAGCGCCAAGCTTATGAACCGA<br>AACTACCGCGGTGAAGCCATTTATACTGCTGAGGTCGATGTGCATTTCCCTATGATGACTGTCGGCGAAACCTT<br>GTACTTTGCTGCACAAGCTCGACGCCCACGACATATTCCTGGAGGTGTGAGCATTCAGCAGTATGCTGAGCACC<br>AGCGTGATGTCATTATGGCCTTGTAAGTACACCTTTGTTCTTCCTGTTACACGCACTAATCATGTTTCCCAGGTA<br>CGGCATCTCACACACAATCAATACCCGCGTTGGAAACGACTTCCTTCGTGGTGTCTCCGGTGGAGAGCGAAAAC<br>GTGTCACCATCGCAGAAGCTTCTCTCAGCAGAGCTCCTTTACAGGCATGGGATAACTCTACCCGTGGGTTGGAT<br>TCTGCCAACGCCATCGAGTTCTGTAAGACACTTCGTATGGAGACTGAAGTCTCAGGAACAACTGCCTGCGTTGC<br>CATCTACCAAGCCCCTCAGGCTGCCTACGACCTCTTCGACAAGGCTCTGGTGCTTTACGAGGGCCGCCAAATCT<br>ATTTCGGATCAACGACCGAGGCAAAGCAATACTTTGTCAATATGGGCTTCGATTGCCCTGACCGCCAGACAGAT<br>GCCGATTTCCTTACCTCCATGACAAGTCCTTTGGAGCGAGTGGTCCGCCCTGGCTGGGAAGATCGTGTGCCTCG<br>TACACCTGATGAGTTTGCTCAACGGTGGAAGGATTCTCAGGAACGAGCCGCACTGCTGGCTCAAATCGAGGAC<br>TACGAACAAAGGTACCCTATTGGTGGTGAAGAGGCGCAGAAATTTGCCGAATCCCGCAAGGCTGAACAGGCCA<br>AGGGTCAGCGCGCAAAGTCTCCCTATACTCTATCCTATGCTCAACAGGTCAAACTCTGTTTGTGGCGTGGATTT<br>GTAAGATTAAAGGCTGACCCTAGTATCACGCTCACACAGCTTTTCTTCAACATGATCATGGCTTTGATCATTTCA

TCCGTCTTTTACAACCTGTCTCCCACCACTCAAAGCTTCTACTCCCGATCGGCCTTGCTCTTCTTCGCTATCTTGA
TGAACGCCTTCAGTTCTGCTCTCGAAATCTTGACGCTCTACGCACAACGACCGATCGTCGAAAAGCACGCGCGA
TATGCTCTTTACCACCCATCCGCCGAGGCCTTCGCTAGCATGTTGACAGATATGCCATACAAGATCCTCAACAC
GATCTTCTTCAATTTGGTACTCTACTTCATGACCAATTTGCGGCGTGAACCAGGCTATTTCTTCTTCTTCCTTCTC
ATCTCGTTCACCCTGACCCTGGTCATGTCCATGTTCTTCCGCTCTATTGCCGCTTTGTCACGCTCCCTCGTGCAAG
CGTTGGCTCCAGCCGCTATCCTCATCCTGGCTCTTGTCATGTATACTGGGTTTACCATCCCACCTACCTACATGC
GAGGCTGGGCCAAATGGATTCGATACATCAATCCTGTCAGCTATGGCTTCGAAGCCCTCATGGTCAACGAATTC
CACAACCGAAACTTCACATGCAACCAGTTCATCCCAAGCGGACCTGGTTATACCGGCATTGGTGGAGTCAACCA
TGCTTGTTCTGCTGTTGGTTCGGTCCCAGGACAACCTTACGTCAACGGAGACGCGTACATTGGCCTGTCGTTCAA
CTACAAGGCTTCACACAAATGGCGCAACTTTGGCTTGCTGTGGGCATTTTTGGTCGTTCTCATGGGAGTGTACTT
GGTCGCAACTGGACTTATTACTGCTGCCAAATCCAAGGGTGAGGTGCTCGTCTTCCGACGTGGTCACAAAGCCA
TCAAGGGCAAGGCATCTGATGATGAAGAAGCTGTTCCCGCTGGACGCACTGTTTCTACACAGTATGACAACTCT
GGTGACAACATCGCCATCATTGAACGCCAACGTATTCTAGACCACGTCGATGGTTGGGTTAAGCCTGGTACTCT
TACTGCACTGATGGGTGTATCTGGTGCTGGAAAAACAACCCTCCTGGATTGCTTGGCCACACGTACTACTATGG
GTGTCATTACTGGTGAAATGTTGGTCGATGGTAAGCCGCGTGACGATTCCTTCCAGCGCAAGACTGGTTACGCA
CAGCAACAAGACCTTCATTTGTCTACCTCGACCGTACGAGAAGCATTGTCCTTCTCCGCCCTCCTCCGTCAGCCG
GCTCATGTCTCCCGCAAAGAAAAGCTCGAATATGTCGATGAAGTTATCAAACTCTTGGAAATGACCGAGTATGC
CGATGCCGTCGTTGGTGTGCCTGGTGAAGGTCTCAATGTTGAACAGCGTAAGCGCCTCACTATCGGTGTTGAAC
TTGCTGCGAAACCGGCTCTTCTTCTCTTTTTGGACGAACCTACTTCTGGTCTGGATTCGCAGACTAGTTGGGCTA
TTCTCGATCTTTTGGACAAGCTGAAGAAGAATGGCCAAGCTATCTTGTGTACTATCCATCAGCCATCCGCCATG
CTTTTCCAGCGTTTCGATCGCCTCTTGTTCCTCGCCAGGGGTGGTAGGACTGTTTACTACGGCGATGTTGGCGCC
AACTCGAAGATCCTCGTCGACTACTTCGTCCGCAACGGTGGACCTGAGTGCCCTCCCGAAGCTAACCCCGCTGA
ATGGATGTTGGAAGTCATCGGCGCTGCCCCCGGCTCTCATACTGAAATCGACTGGCCTCAGGTGTGGCGTAGCT
CGCCTGAGTACGCTGAAGTCCAACGCCATCTCGCTGAACTCAAATGCGAACGCTCCGCCATGACAGATCAAATT
CAACGGACGGTCTCAGCTCAAAAGCGAGAGGATAAGGCTGCTTATCGCGAATTTGCTGCTCCTTTCAGTGAGCA
ACTTCGCGAGTGCCTTGTTCGTGTTTTCCAGCAATACTGGCGCAGCCCCTCGTACATCTACTCCAAGACAGTTCT

CTGTGTTTCCTCGGCTCTCTTCATTGGGTTCTCGCTCTATCAGATGCCCAACACGCAGCAGGGTCTTCAAAACCA
GATGTTTGCCATTTTCATGTTGTTGACTATTTTCGGTCAGCTCGTCCAACAGATCATGCCACACTTTGTTACACA
GCGCGCTTTGTATGAGGTGCGTGAGCGACCCAGCAAGGCTTACTCCTGGAAAGCATTCATGATGTGCAACATTA
TTGTTGAACTTCCTTGGAACTCGCTCATGGCTGTGCTCATCTATTTCTGCTGGTACTACCCCATCGGCCTTTATCG
CAACGCTTCGCCTACCGACGCCGTTGCCCTCCGTGGCTTCCAGTCTTTCTTGTTCGTCTGGATGTTCCTCCTCTTT
ACATCGACATTCACACACATGATCATTGCTGGTATGGATTCTGCTGAGAATGGTGGTAACCTGGCCAATCTGAT
GTTCTCACTCTGCCTCATCTTCTGCGGTGTTCTCGCTCAGCCATCGCAATTCCCAGGCTTCTGGATCTTCATGTAC
CGCGTCTCGCCATTCACTTACCTTGTCAGTGGCATGTTAGCTGTCGGTCTAGCCAACAACGTTGTCGAATGTTCT
GACACGGAATACGTCAAATTCCAGCCCACCGACAACCAAACCTGCGGACGCTACATGGAGAACTGGATGTCCG
CTATGGGTGGCTACGTCCAGAACCCCGAAGCCAGATCCGACTGTCGCTTCTGTTCCATCCGCGAAACCAACAAA
TTCTTGGCGAACATCTCGTCGGATTACAGCGACGCCTGGCGCAATTTCGGCATTCTCTGGGCTTACGTTATCTTC
AACATCGTTGCTGCTCTCGCTCTATACTGGCTCGTCCGTATGCCCAAGCCCAAGAAGGAAAAGGAAACCAAGG
ACGACACTCCTGCACCCAAGTCCGTCGCTCAGGAACAAAGCTCAGATAGCTCCATCCACGAGGGCTCGCAGCA
AGAAAAGCAAGAAACACATGGCGAATCAGCACAGCGATACGCCTCCATCACCGGCGCAGAAACCCCACCTCGC
TCCAAGGGTGAGGACGGCGCCGAAAAAGTCTAGACTTTGATTTTGATTTGAATCGTTATTACCATTGTTCATCA
TTACCAATATCACTCCCACTACTGCCACTAGATTGTTCATACTCCAAGCACCTTCCACCGCATTGCCACGGCCAT
ATTCTTGTGTTGTTTTGATCATTTTTGGCCATTTTTGGCTCCACCACCTACCTACCAGTATATATAAACCCACTCT
TACCTTTTTTTTGTTTGCTTCTACAAGGAGGAATTCATCGAATAGATTTAGATTTCGTCTTTGGCTTTTTTTTTTTGT
TACGTTCTTGACTCCTACTCAAGCGCTGGAGGTCTTACTACATTTTTGGTCTTTGGTTGCAATGTGGATATGTAA
GCGGTACATAGTAGTGTAGGATTAATTATATTATTACTTGGGGAAAAAAAAAAAAAAAAAAAAAAAAAAAAAA
A

FAD	F: 5’-GATGGTTGCGTTGGCAAGTT-3’<br>R: 5’-GCTTCCCACCCATACGACAA-3’	FAD/FMA-dependent oxidoreductase, *Shiraia* sp. slf14 [AIW00665.1]	ATGTTTGTGGAAAACCTGCCGCAGTGGATTCTGGCGCTGGCGAAATTTGTGCCGTATCATGCGGGCGAAGGCCTG<br>CCGGTGCTGACCCGCCCGCAGCGCATTGAAGCGCAGAAACATGGCGATCAGGTGAACGCGGCGTGGGAAGCGG<br>TGAGCAGCAGCAACAGCTGCTGCGAAGCGGTGGCGAAACAGCTGAAAGATCGCGTGGCGTTTCCGGAAGGCAA<br>AGCGTATAGCCAGAGCGTGAACAGCTATTGGAGCCTGAAAGAAGTGGAAATTCATCCGAGCTGCGTGGTGCTGC<br>CGGAAAGCGCGGATGAAGTGAGCCAGATTGTGCAGACCCTGAGCGCGGGCATGCATGTGTTTAAAGATCAGTGC<br>CAGTTTGCGATTCGCGGCGCGGGCCATGCGCCGTTTGCGGGCGCGAGCAACATTAAAAACGGCGTGGTGATTCA<br>TCTGAAAAACATGCCGAGCGCGGGCCTGGCGCCGGATCATAAAACCATTACCGTGAGCCCGAGCACCACCTGGG<br>ATCAGGTGTTTGCGACCCTGGATGTGTTTGATCTGGCGACCCTGGGCGGCCGCGTGGGCGGCGTGGGCGTGGGC<br>GGCCTGACCACCGGCTGCGGCATTAGCTATTTAGCCCGCGCTTTGGCTTTACCTGCAACATGGTGGAAAACTTT<br>GAAGTGGTGCTGGCGAACGGCGATATTGTGAACGCGAACGCGACCAGCCATCGCCCGCTGTGGAAAGCGCTGC<br>GCGGCGGCAGCAACAACTTTGGCGTGGTGACCGCGATTACCCTGCGCACCTTTCCGCAGGGCAAATTTTGGGGC<br>GGCCAGACCTTTCATCCGATTGATACCCGCAAAGCGCATTTTCATGCGCTGGAAAACCTGATTGCGAGCCATCCG<br>TATGATAAATATGCGCATTTTATTAACAACCTGGTGACCACCAACGCGAGCTATGGCAACTGGTTTATTGGCAACA<br>GCCTGCAGTATACCAAAAGCGATCCGCCGAGCCCGTTTCCGGAAACCTTTAAACCGTTTACCGATATTCCGCGCG<br>CGCCGCTGTTTCCGGGCGCGCCGGATAACACCCTGCGCGTGGATAACCATACCGCGTTTACCCTGGAATATGCGG<br>CGCTGAGCGTGTATCCGAAACGCTGGCAGTTTGCGACCATTAGCTTTGGCAACAGCGCGGAAATGATGGAAGAT<br>TTTTTTCAGCTGGCGAACGCGACCATTCAGCCGTTTATTAGCCTGCCGGGCTTTCTGGTGAGCATTGCGTTTCAGC<br>CGCTGCCGACCCTGATTAGCGAACGCTATGGCGCGGTGGATAGCCTGGGCCCGATTCAGACCCAGGGCAACATG<br>TTTTATATTCATTGGGCGATGAGCGTGGATGGCAGCGAAGGCAGCACCGATGCGGCGTTTGAACAGGCGACCGA<br>AACCCTGTTTCGCGAAGCGGAACGCAAAGCGGCGGCGAAAGGCCTGCAGCGCGATTTTCTGCAGCTGACCTATG<br>CGGATCGCTGGCAGGATCCGCTGGGCCGCCGCAGCAAAGGCACCGTGAAAGAACTGTGGGAAACCAGCCTGCT<br>GTATGATCCGCATGGCCTGTTTCAGACCCAGGTGCCGGGCGGCTTTAAACTGCCGCAGACCAAAGCGCGCGAAA<br>ACGCGGAACTG

MCO	F: 5’-CCCATCAAACCATTCGTCGC-3’<br>R: 5’-ATTTTGTGGCCGAGGTCCAT-3’	Multicopper oxidase, *Shiraia* sp. slf14 [AIW00656.1]	ATGGCGTGCAACGCGTTTAAATTTTTTACCGGCCTGCTGGGCCTGAGCAGCACCGTGAGCCGCAGCTTTCCGTTT<br>AGCTGCAGCAAAGCGAGCGTGCGCAGCAGCGATACCGTGCGCTTTGAAATTCATATTACCGCGGGCAGCGTGAA<br>CCCGACCGGCACCGGCTTTCGCGATGCGATTCTGGTGAACGGCACCTTTCCGGGCCCGACCCTGCATATTAACCG<br>CGGCGATAACGTGGAATTTCTGGTGCGCAACTATCTGCGCGAAGATACCACCGTGCATTTTCATGGCATTACCCAG<br>GGCGTGAGCCCGTGGGCGGATGGCGTGCCGGGCGTGACCCAGCGCCCGATTCGCCCGGGCGCGAGCTATCTGTA<br>TCGCTGGAAAGCGGATGAAAGCGGCGTGTATTTTTATCATGCGCATAGCCGCGGCCAGATGATGGATGGCCTGTAT<br>GGCGCGATTATTATTAACCCGAGCGAAAACGAACCGAACCCGTTTCATATGATTAGCCATGATCCGCAGGATTGGG<br>AAAAAATGCGCAAAGCGGAAAAAAACATGCAGACCCTGATGATTAGCGATTGGAGCCAGTATAAATTTCGCGAT<br>TTTATGCGCGTGGAAGAAAACGCGAACATTGATTATACCTGCATGGATGCGATTATTGTGAACGGCGCGGGCAGC<br>CAGTATTGCCTGGATCGCGAACTGCTGAACGAATATACCGATCCGCTGGTGAAATATATTCTGGGCAGCACCGATG<br>AAAAAGAAATTACCGATAAAGGCTGCGTGCCGCCGCTGCAGCTGTTTCAGGGCAACTTTAGCCTGCATCTGGATA<br>CCCTGCCGCGCGAAGCGTATCATAAATGCATTCCGGGCGTGGGCGGCGGCGCGAACCATACCGTGACCGTGCATA<br>GCGGCAAAGGCTGGGCGGCGCTGACCTTTATTAACCCGGGCGGCCTGTATCCGCTGAAAGTGACCATTGATAACC<br>ATCTGCTGCATGTGTATGCGATTGATGGCCAGTATATTTATCCGCAGGCGGTGGATCAGATTCTGGTGAACAACGG<br>CGAACGCTATAGCGTGCTGCTGAAACTGGATCAGTATGTGGGCCGCTATACCATTCGCATTGCGAACGATCTGCTG<br>GGCCAGGTGCTGGGCGGCTTTGGCGCGATTAGCTATAACGGCAACCTGGAAGATCCGCCGAACGTGCGCCCGCT<br>GATGAACTATGCGGGCAAACCGATTGTGGATGAAATTCGCGTGTTTGATGATTATAACATTCGCCCGTATCCGCCG<br>CGCCCGCCGGCGGCGAGCGCGGATCGCACCCATAAATTTCTGGTGCGCAAACTGGGCCAGCCGTATGGCGCGTA<br>TGAATGGACCCTGAGCGGCGATGAAGGCTATAACGCGAGCGCGGAAGATATTGCGAGCCCGTTTCTGTTTCAGC<br>GCCCGAAAAGCATTGAAACCAGCGAACTGCTGCTGCGCAACCAGAAAAACGAATGGGTGGATCTGATTATTGAA<br>ATTGAAGGCCCGTTTGCGCAGAGCCATCCGATGCATAAACATGGCAACAAAGCGTATGTGATTGGCCAGGGCGTG<br>GGCTTTTTTCCGTGGGCGACCGTGGCGGAAGCGGAAAAAGCGCTGCCGAAAGGCACCTTTAACTTTATTGAACC<br>GCCGTATAAAGATACCTTTAAAACCCTGGAAGGCGTGAACAACAACGCGTGGCTGGTGCTGCGCTATAAAGTGG<br>ATAGCCCGGGCGCGTGGCTGTTTCATTGCCATATTCAGACCCATCTGAGCGGCGGCATGGGCGTGGTGATGCTGG<br>ATGGCGTGGATGCGTGGCCGGAACTGCCGGAACGCTATAAAGAATGGAACGGCTTTGATCAGCCGAAA

ZFTF	F: 5’-TACGATGAGCGCCAACAACT-3’<br>R: 5’-CTGCATGGCTATCGGGGATT-3’	Zinc finger transcription factor, *Shiraia* sp. slf14 [AIW00663.1]	ATGGCGAAAAAAAAACTGAACAGCACCATTAAACGCAGCCGCACCCGCAGCGGCTGCGTGACCTGCCGCGATC<br>GCCATATTAAATGCGATGAACAGCAGCCGGTGTGCAAAAACTGCCAGAAAAGCAACCGCAAATGCTATCGCGGC<br>ATTCGCCTGAACTTTACCCAGTATACCTTTTATAACCCGGATGATAACAAACCGAAAGAACTGCAGCAGAACGAA<br>CAGCCGAACAGCAGCCATTATGCGTTTCCGAACCTGGAACCGAACCCGGTGAGCCAGAAACATCGCATTCTGGA<br>TCAGAGCATTACCATTGCGAGCCTGTATGATGATCTGAAAAAATATAAACCGTATATTCATCTGCATACCCCGGAA<br>GATCTGCGCGAAAGCGATCTGCAGTTTCAGGAAGATACCTATAACAGCTATATTAGCACCAGCGCGATTAACCTGC<br>GCGGCAAAAAACTGACCAAACGCGATCCGGGCCTGAGCACCAGCCTGAGCGTGATTAACCCGACCCTGGAAAG<br>CGAAATTAAACCGAACCCGGTGATTCTGAACCAGCTGAGCTTTCATCCGCCGCCGAACCTGAACACCGGCGTGC<br>TGTATCCGCCGACCGCGACCGCGGCGACCACCACCACCAGCAGCCCGACCAACCATCATCTGCATCCGTATTTTG<br>TGAGCAGCATTCCGAACCCGCAGCATCATCCGATGCTGGATACCAGCCAGCATCAGGAAACCACCAGCACCGAT<br>CCGAACCAGTTTGATTATAGCCATCTGAGCATGCCGCAGAGCACCCCGCTGCTGATGAAATATGATATTACCACCT<br>ATGTGCGCCTGATTGAAACCGAAAAATATTATATGCTGCTGGATCTGGCGAACGAACTGGATATTTGGAAAAAAAT<br>TATTCCGAGCCTGTGCCTGCAGATTAGCGAAAACGATAGCTTTCTGCTGGATTGCCTGATGAGCTGCAGCCGCAA<br>CACCAGCGTGAACCTGCTGGATCTGACCAACGAACAGCTGAACAAATGGAGCCAGCTGAAAAACGCGCCGGTG<br>ATTAGCGAACGCATTCAGCAGTTTGAACATATTCTGATTAGCATTGTGCTGATTCTGCTGGGCCTGTATCTGAACA<br>CCACCAAAGTGCGCCTGACCGATTATCATAAAGTGATTTTTAACAACCAGGCGAAACTGTTTAGCCATGTGCTGC<br>GCAAAATTCATACCTTTATTACCAGCAACAAACCGAACAGCGCGGTGCTGACCAACGCGATTCAGAGCATTACCA<br>TGCTGAAATTTTTTATTGATAAAAACTATGATTTTAGCTATGAATTTAAAAACATTCAGAAAGGCCGCGTGACCGA<br>TACCAGCGAAGAAATTACCTATAGCAACAGCAACCTGTATAGCAACCCGGATATTAGCTATATTAGCACCTTTAAC<br>GAATATGAAATTATTTATCTGAACAACAGCTATCAGAACCTGGTGCATGTGGATCAGAGCAACAGCATGAGCATG<br>GGCGAAAGCCAGCTGTATAAAGATCTGCTGTGGTATCTGATGAAAGTGGATTTTGTGATTAACTATCCGGAAGCG<br>GCGAACAACCTGGTGCTGGATCATAACGTGGTGTATCAGCAGATTACCAACGCGAGCACCGATCTGAGCTTTAGC<br>AACAACCTGAACTATCTGAACCCGCGCAGCTATGCGAACTATTTTCTGAAAGAATTTATTATTAAAGTGCTGAGCA<br>TGGGCAGCAACGCGATTATTGAAGATGCGAACAACCGCATTAACACCCTGTTTAACTTTATTGATCAGAGCTATAT<br>GGATCCGGAACTGAAAAGCCAGTTTCATCATTGCTTTACCTGGACCGTGCGCTATATTCATCCGGTGAGCGAT

Omef	F: 5'-GCTGGTGGACCTCTCCTTTC-3'<br>R: 5'-AGCGTGCCTTCAGGTAGTTC-3'	*O*-methyltransferase, *Shiraia* sp. slf14 [AIW00661.1]	ATGAGCCTGTTTCAGCTGACCGCGGCGGTGACCAAAGCGGCGGCGGATCTGGATGCGGCGAGCGTGGCGGCGG<br>TGGATGCGGCGAACGTGGAAGCGAAAGCGAAAGCGAGCCTGCTGGATGCGGCGGAACGCCTGGTGAACGCGTA<br>TCGCAGCCCGCGCCAGTGGCTGGTGGATCTGAGCTTTCAGCATTGCGCGACCGCGACCCTGCAGCTGATTATGCG<br>CTATCGCCTGCATCATGCGGTGCCGCTGCAGGGCAGCATTAGCTTTGGCGAAATTGCGGATGCGATTGCGGCGAG<br>CGAAAAAAAAAGCAAAGTGCCGGCGAGCCTGGTGGAACGCCTGCTGCAGCATGCGCTGAGCTTTGGCCTGTTT<br>ACCCCGGCGGCGAACGGCCGCATTGCGCATAACGATGCGAGCCGCCTGCTGGTGACCGATCCGGATCTGGAAGC<br>GTGGATGTATCTGTGCAGCAACATTGCGTATCCGGCGGGCGCGCAGCTGCCGAAAGCGATTGAACAGTATGGCGC<br>GAGCAGCGAACCGAACGAAACCGCGTATAGCGTGAGCATTGGCCGCCGCATTAGCCAGTTTGAACGCTTTCGCG<br>AACCGGATGGCCATGCGGAACATGAAATGTTTGCGCGCGCGATGAAAGGCATTAGCGCGGGCGGCGCGTATGAT<br>GTGGGCCATGTGGTGGATGGCGGCTATCCGTGGCATGAACTGCCGCAGGGCACCCTGGTGGTGGATGTGGGCGG<br>CGGCCCGGGCCATGTGAGCATGGCGCTGGCGCGCAAATATCCGCAGCTGAGCTTTGAAGTGCAGGATCTGGTGG<br>AAACCGCGGAATTTGGCAGCCGCAGCTGCCCGGCGGAACTGAAAGAACGCGTGAGCTTTCGCGCGCATGATTTT<br>TTTACCCCGCAGCCGCAGCATCGCACCGCGGAAGATCAGCGCGCGGTGGTGTATTTTGCGCGCTTTATTTGCCAT<br>GATTGGAGCGATAAATATGCGGGCCAGATTGTGGGCGCGGTGGCGGCGCAGATGCGCGAACAGGATCGCCTGAT<br>TCTGAACGAAGTGATTGTGCCGGAACCGGGCGAAATGGGCCAGCGCATTGAACGCAAATGCCAG

*The nucleotide sequence of *ABC* was reported in our previous study (Lu et al. 2019. Journal of Rare Earths 37: 895-902. https://doi.org/10.1016/j.jre.2018.10.010)

Table S3 16S rDNA gene sequence analysis of actinomycetes isolated from *Shiraia* fruiting body based on BLAST analysis in the NCBI database

Strain No.	Nearest match in NCBI database and accession No.	Query coverage (%)	Identity (%)
A4	*Streptomyces* sp. (MN620416.1)	100	100
A5	*Streptomyces* sp. (LC133696.2)	100	100
A6	*Streptomyces pseudogriseolus* (LC034307.1)	100	99.86
A7	*Streptomyces venezuelae* (CP029190.1)	100	99.78
A8	*Streptomyces tendae (*KF454844.1)	100	100
A9	*Streptomyces* sp. (CP036534.1)	100	99.93
A10	*Streptomyces* sp. (KM191334.1)	100	99.93
A11	*Streptomyces tendae* (NR_025871.2)	100	99.73
A12	*Streptomyces* sp. (MH806379.1)	100	99.93
A13	*Streptomyces* sp. (KY613504.1)	100	100
A14	*Streptomyces pactum* (MG984583.1)	100	100
A15	*Streptomyces olivaceus* (CP016795.1)	100	100
A16	*Pseudonocardia* sp. (EF588219.1)	100	99.93
A17	*Streptomyces* sp. (CP045547.1)	100	100
A18	*Streptomyces* sp. (CP036534.1)	100	99.86
A19	*Pseudonocardia* sp. (MG195138.1)	100	99.93
A21	*Streptomyces physcomitrii* (MH715906.1)	100	100
A22	*Pseudonocardia* sp. (LC260150.1)	99	100
A23	*Pseudonocardia* sp. (CP011862.1)	100	99.79
A24	*Streptomyces* sp. (MG437309.1)	100	99.93
A25	*Pseudonocardia* sp. (MN631183.1)	100	99.52
A26	*Pseudonocardia* sp. (EF588221.1)	100	99.85
A27	*Streptomyces albus* (CP033071.1)	100	99.52
A28	*Pseudonocardia* sp. (AM403499.1)	100	99.93
A29	*Streptomyces* sp. (MG984583.1)	100	100
A30	*Pseudonocardia* sp. (EF588219.1)	100	99.93
A31	*Brevibacterium* sp. (KU588052.1)	98	97
A32	*Rhodococcus* sp. (OP001416.1)	99	96

Table S4 Taxonomic classification of 28 actinomycetes isolates from *Shiraia* fruiting bodies

Strain No.	Nearest match in NCBI database and accession No.	Identity (%)	Phylum	Class	Order	Family	Genus	species
A4	*Streptomyces* sp.*(MN620416.1)*	100.00	Actinobacteria	Actinomycetia	Actinomycetales	Streptomycetaceae	*Streptomyces*	unclassified *Streptomyces*
A5	*Streptomyces* sp.*(LC133696.2)*	100.00	Actinobacteria	Actinomycetia	Actinomycetales	Streptomycetaceae	*Streptomyces*	unclassified *Streptomyces*
A6	*Streptomyces rubiginosus(LC034307.1)*	99.86	Actinobacteria	Actinomycetia	Actinomycetales	Streptomycetaceae	*Streptomyces*	*Streptomyces rubiginosus*
A7	*Streptomyces venezuelae(CP029190.1)*	99.78	Actinobacteria	Actinomycetia	Actinomycetales	Streptomycetaceae	*Streptomyces*	*Streptomyces venezuelae*
A8	*Streptomyces tendae(KF454844.1)*	100.00	Actinobacteria	Actinomycetia	Actinomycetales	Streptomycetaceae	*Streptomyces*	*Streptomyces tendae*
A9	*Streptomyces* sp.*(CP036534.1)*	99.93	Actinobacteria	Actinomycetia	Actinomycetales	Streptomycetaceae	*Streptomyces*	*Streptomyces pactum*
A10	*Streptomyces sp. BCA-671(KM191334.1)*	99.93	Actinobacteria	Actinomycetia	Actinomycetales	Streptomycetaceae	*Streptomyces*	unclassified *Streptomyces*
A11	*Streptomyces tendae(NR_025871.2)*	99.73	Actinobacteria	Actinomycetia	Actinomycetales	Streptomycetaceae	*Streptomyces*	*Streptomyces tendae*
A12	*Streptomyces* sp.*(MH806379.1)*	99.93	Actinobacteria	Actinomycetia	Actinomycetales	Streptomycetaceae	*Streptomyces*	unclassified *Streptomyces*
A13	*Streptomyces* sp.*(KY613504.1)*	100.00	Actinobacteria	Actinomycetia	Actinomycetales	Streptomycetaceae	*Streptomyces*	unclassified *Streptomyces*
A14	*Streptomyces pactum(MG984583.1)*	100.00	Actinobacteria	Actinomycetia	Actinomycetales	Streptomycetaceae	*Streptomyces*	*Streptomyces pactum*
A15	*Streptomyces olivaceus(CP016795.1)*	100.00	Actinobacteria	Actinomycetia	Actinomycetales	Streptomycetaceae	*Streptomyces*	*Streptomyces olivaceus*
A16	*Pseudonocardia* sp. *(EF588219.1)*	99.93	Actinobacteria	Actinomycetia	Pseudonocardiales	Pseudonocardiaceae	*Pseudonocardia*	unclassified *Pseudonocardia*
A17	*Streptomyces* sp. *(CP045547.1)*	100.00	Actinobacteria	Actinomycetia	Actinomycetales	Streptomycetaceae	*Streptomyces*	unclassified *Streptomyces*
A18	*Streptomyces* sp. *(CP036534.1)*	99.86	Actinobacteria	Actinomycetia	Actinomycetales	Streptomycetaceae	*Streptomyces*	unclassified *Streptomyces*
A19	*Pseudonocardia sp. (MG195138.1)*	99.93	Actinobacteria	Actinomycetia	Pseudonocardiales	Pseudonocardiaceae	*Pseudonocardia*	unclassified *Pseudonocardia*
A21	*Streptomyces physcomitrii(MH715906.1)*	100.00	Actinobacteria	Actinomycetia	Actinomycetales	Streptomycetaceae	*Streptomyces*	*Streptomyces physcomitrii*
A22	*Pseudonocardia* sp. *(LC260150.1)*	100.00	Actinobacteria	Actinomycetia	Pseudonocardiales	Pseudonocardiaceae	*Pseudonocardia*	unclassified *Pseudonocardia*
A23	*Pseudonocardia* sp. *(CP011862.1)*	99.79	Actinobacteria	Actinomycetia	Pseudonocardiales	Pseudonocardiaceae	*Pseudonocardia*	unclassified *Pseudonocardia*
A24	*Streptomyces* sp. *(MG437309.1)*	99.93	Actinobacteria	Actinomycetia	Actinomycetales	Streptomycetaceae	*Streptomyces*	unclassified *Streptomyces*
A25	*Pseudonocardia* sp. *(MN631183.1)*	99.52	Actinobacteria	Actinomycetia	Pseudonocardiales	Pseudonocardiaceae	*Pseudonocardia*	unclassified *Pseudonocardia*
A26	*Pseudonocardia* sp. *(EF588221.1)*	99.85	Actinobacteria	Actinomycetia	Pseudonocardiales	Pseudonocardiaceae	*Pseudonocardia*	unclassified *Pseudonocardia*
A27	*Streptomyces albus(CP033071.1)*	99.52	Actinobacteria	Actinomycetia	Actinomycetales	Streptomycetaceae	*Streptomyces*	*Streptomyces albus*
A28	*Pseudonocardia* sp. *(AM403499.1)*	99.93	Actinobacteria	Actinomycetia	Pseudonocardiales	Pseudonocardiaceae	*Pseudonocardia*	unclassified *Pseudonocardia*
A29	*Streptomyces* sp. *(MG984583.1)*	100.00	Actinobacteria	Actinomycetia	Actinomycetales	Streptomycetaceae	*Streptomyces*	unclassified *Streptomyces*
A30	*Pseudonocardia* sp. *(EF588219.1)*	99.93	Actinobacteria	Actinomycetia	Pseudonocardiales	Pseudonocardiaceae	*Pseudonocardia*	unclassified *Pseudonocardia*
A31	*Brevibacterium* sp. *(KU588052.1)*	97.00	Actinobacteria	Actinobacteria	Actinobacterales	Corynebacteriaceae	*Brevibacterium*	unclassified *Brevibacterium*
A32	*Rhodococcus* sp. *(OP001416.1)*	96.00	Actinobacteria	Actinobacteria	Actinobacterales	Nocardiaceae	*Rhodococcus*	*unclassified Rhodococcus*

Table S5 Number of bacterial sequences by high-throughput sequencing in *Shiraia* fruiting body

Sample	16S rRNA sequences	*Actinomycetes* sequences	Percent %	Length average
Shiraia fruiting body	37,610	483	1.3	420

Table S6 Taxonomic classification of OTUs detected from the fruiting bodies with read numbers of the fruiting body-associated actinomycetes

OTU ID	Reads	Taxonomy
209	1	k__Bacteria; p__Actinobacteria; c__Thermoleophilia; o__Solirubrobacterales; f__unclassified__Solirubrobacterales(o); g__unclassified__Solirubrobacterales(o); s__unclassified__Solirubrobacterales(o)
235	2	k__Bacteria; p__Actinobacteria; c__Thermoleophilia; o__Solirubrobacterales; f__unclassified__Solirubrobacterales(o); g__unclassified__Solirubrobacterales(o); s__unclassified__Solirubrobacterales(o)
251	20	k__Bacteria; p__Actinobacteria; c__Thermoleophilia; o__Gaiellales; f__Gaiellaceae; g__unclassified__Gaiellaceae(f); s__unclassified__Gaiellaceae(f)
258	1	k__Bacteria; p__Actinobacteria; c__Thermoleophilia; o__Gaiellales; f__Gaiellaceae; g__unclassified__Gaiellaceae(f); s__unclassified__Gaiellaceae(f)
279	1	k__Bacteria; p__Actinobacteria; c__Actinobacteria; o__Actinomycetales; f__Actinomycetaceae; g__Actinomyces; s__unclassified__Actinomyces(g)
399	3	k__Bacteria; p__Actinobacteria; c__Actinobacteria; o__Actinomycetales; f__Nocardioidaceae
415	1	k__Bacteria; p__Actinobacteria; c__Actinobacteria; o__Actinomycetales; f__Actinomycetaceae; g__Actinomyces; s__unclassified__Actinomyces(g)
428	1	k__Bacteria; p__Actinobacteria; c__Actinobacteria; o__Actinomycetales; f__Corynebacteriaceae; g__Corynebacterium; s__durum
431	12	k__Bacteria; p__Actinobacteria; c__Actinobacteria; o__Actinomycetales; f__Microbacteriaceae
440	11	k__Bacteria; p__Actinobacteria; c__Actinobacteria; o__Bifidobacteriales; f__Bifidobacteriaceae; g__Bifidobacterium; s__adolescentis
443	4	k__Bacteria; p__Actinobacteria; c__Actinobacteria; o__Actinomycetales; f__Geodermatophilaceae
444	5	k__Bacteria; p__Actinobacteria; c__Actinobacteria; o__Actinomycetales; f__Corynebacteriaceae; g__Corynebacterium
445	2	k__Bacteria; p__Actinobacteria; c__Actinobacteria; o__Actinomycetales; f__Geodermatophilaceae; g__Geodermatophilus; s__unclassified__Geodermatophilus(g)
447	1	k__Bacteria; p__Actinobacteria; c__MB-A2-108; o__0319-7L14; f__unclassified__0319-7L14(o); g__unclassified__0319-7L14(o); s__unclassified__0319-7L14(o)
449	5	k__Bacteria; p__Actinobacteria; c__Actinobacteria; o__Actinomycetales; f__Pseudonocardiaceae; g__Pseudonocardia
450	5	k__Bacteria; p__Actinobacteria; c__Actinobacteria; o__Actinomycetales; f__Intrasporangiaceae
451	2	k__Bacteria; p__Actinobacteria; c__MB-A2-108; o__0319-7L14; f__unclassified__0319-7L14(o); g__unclassified__0319-7L14(o); s__unclassified__0319-7L14(o)
452	1	k__Bacteria; p__Actinobacteria; c__Actinobacteria; o__Actinomycetales; f__Corynebacteriaceae; g__Corynebacterium; s__unclassified__Corynebacterium(g)
453	48	k__Bacteria; p__Actinobacteria; c__Actinobacteria; o__Actinomycetales; f__Propionibacteriaceae; g__Propionibacterium; s__acnes
454	1	k__Bacteria; p__Actinobacteria; c__Actinobacteria; o__Actinomycetales; f__Nocardioidaceae; g__Nocardioides; s__unclassified__Nocardioides(g)
455	5	k__Bacteria; p__Actinobacteria; c__Actinobacteria; o__Actinomycetales; f__Mycobacteriaceae; g__Mycobacterium; s__unclassified__Mycobacterium(g)
456	84	k__Bacteria; p__Actinobacteria; c__Actinobacteria; o__Actinomycetales; f__Intrasporangiaceae
457	1	k__Bacteria; p__Actinobacteria; c__Actinobacteria; o__Actinomycetales; f__Mycobacteriaceae; g__Mycobacterium; s__unclassified__Mycobacterium(g)
458	7	k__Bacteria; p__Actinobacteria; c__Actinobacteria; o__Actinomycetales; f__Microbacteriaceae
459	2	k__Bacteria; p__Actinobacteria; c__Actinobacteria; o__Actinomycetales; f__Dietziaceae; g__Dietzia; s__unclassified__Dietzia(g)
460	7	k__Bacteria; p__Actinobacteria; c__Actinobacteria; o__Actinomycetales; f__Nocardioidaceae; g__Aeromicrobium; s__unclassified__Aeromicrobium(g)
461	4	k__Bacteria; p__Actinobacteria; c__Actinobacteria; o__Actinomycetales; f__unclassified__Actinomycetales(o); g__unclassified__Actinomycetales(o); s__unclassified__Actinomycetales(o)
462	8	k__Bacteria; p__Actinobacteria; c__MB-A2-108; o__0319-7L14; f__unclassified__0319-7L14(o); g__unclassified__0319-7L14(o); s__unclassified__0319-7L14(o)
463	3	k__Bacteria; p__Actinobacteria; c__Actinobacteria; o__Actinomycetales; f__Nocardioidaceae; g__unclassified__Nocardioidaceae(f); s__unclassified__Nocardioidaceae(f)
464	1	k__Bacteria; p__Actinobacteria; c__Actinobacteria; o__Actinomycetales; f__Nocardioidaceae; g__Friedmanniella; s__unclassified__Friedmanniella(g)
465	4	k__Bacteria; p__Actinobacteria; c__Actinobacteria; o__Actinomycetales
466	5	k__Bacteria; p__Actinobacteria; c__Actinobacteria; o__Actinomycetales; f__Microbacteriaceae
467	3	k__Bacteria; p__Actinobacteria; c__Actinobacteria; o__Actinomycetales; f__Microbacteriaceae
468	7	k__Bacteria; p__Actinobacteria; c__Actinobacteria
469	2	k__Bacteria; p__Actinobacteria; c__Actinobacteria; o__Actinomycetales; f__Microbacteriaceae; g__Agrococcus; s__jenensis
470	5	k__Bacteria; p__Actinobacteria; c__Actinobacteria; o__Actinomycetales; f__Corynebacteriaceae; g__Corynebacterium; s__unclassified__Corynebacterium(g)
471	8	k__Bacteria; p__Actinobacteria; c__Actinobacteria; o__Actinomycetales; f__Micrococcaceae
472	2	k__Bacteria; p__Actinobacteria; c__Actinobacteria; o__Actinomycetales; f__Nocardioidaceae; g__unclassified__Nocardioidaceae(f); s__unclassified__Nocardioidaceae(f)
473	1	k__Bacteria; p__Actinobacteria; c__Actinobacteria; o__Actinomycetales; f__Nocardioidaceae; g__unclassified__Nocardioidaceae(f); s__unclassified__Nocardioidaceae(f)
474	3	k__Bacteria; p__Actinobacteria; c__Actinobacteria; o__Actinomycetales; f__Cellulomonadaceae; g__Cellulomonas; s__unclassified__Cellulomonas(g)
475	17	k__Bacteria; p__Actinobacteria; c__Actinobacteria; o__Actinomycetales; f__Nocardiaceae; g__Rhodococcus; s__unclassified__Rhodococcus(g)
476	2	k__Bacteria; p__Actinobacteria; c__Actinobacteria; o__Actinomycetales; f__Streptomycetaceaeg;g__unclassified__Streptomycetaceaeg(f)
477	2	k__Bacteria; p__Actinobacteria; c__Actinobacteria; o__Actinomycetales; f__Micrococcaceae; g__Micrococcus; s__unclassified__Micrococcus(g)
478	2	k__Bacteria; p__Actinobacteria; c__Actinobacteria; o__Actinomycetales; f__Micrococcaceae; g__Rothia; s__dentocariosa
479	2	k__Bacteria; p__Actinobacteria; c__Actinobacteria; o__Actinomycetales; f__Micrococcaceae; g__Kocuria; s__rhizophila
526	1	k__Bacteria; p__Actinobacteria; c__Actinobacteria; o__Actinomycetales
541	118	k__Bacteria; p__Actinobacteria; c__Acidimicrobiia; o__Acidimicrobiales; f__Microthrixaceae; g__Candidatus Microthrix; s__parvicella
542	5	k__Bacteria; p__Actinobacteria; c__Acidimicrobiia; o__Acidimicrobiales; f__C111; g__unclassified__C111(f); s__unclassified__C111(f)
545	4	k__Bacteria; p__Actinobacteria; c__Acidimicrobiia; o__Acidimicrobiales; f__EB1017; g__unclassified__EB1017(f); s__unclassified__EB1017(f)
550	32	k__Bacteria; p__Actinobacteria; c__Acidimicrobiia; o__Acidimicrobiales; f__C111; g__unclassified__C111(f); s__unclassified__C111(f)
565	2	k__Bacteria; p__Actinobacteria; c__Coriobacteriia; o__Coriobacteriales; f__Coriobacteriaceae; g__Collinsella; s__aerofaciens
569	3	k__Bacteria; p__Actinobacteria; c__Acidimicrobiia; o__Acidimicrobiales; f__Microthrixaceae; g__unclassified__Microthrixaceae(f); s__unclassified__Microthrixaceae(f)
683	4	k__Bacteria; p__Actinobacteria; c__Actinobacteria; o__Bifidobacteriales; f__Bifidobacteriaceae; g__Bifidobacterium

Table S7 Diversity index of coral symbiotic actinomyces 16S rDNA gene library

Sample	OTUs	Chao index	PD	Coverage	Shannon	Simpson
Shiraia fruiting body	54	58.014	3.076	0.975	4.172	0.889

Table S8 The OTU taxonomy of actinomycetes community in *Shiraia* fruiting bodies

Sample	Phylum (k)	Class (c)	Order (o)	Family (f)	Genus (g)
Shiraia fruiting body	1	5	8	24	28

Table S9 Taxonomic classification of OTUs detected from the fruiting bodies with read numbers of the fruiting body-associated actinomycetes

Phylum	Class	Order	Family	Genus	Ratio (%)
Actinobacteria	Acidimicrobiia	Acidimicrobiales	Microthrixaceae	*Candidatus Microthrix*	24.43
Actinobacteria	Actinobacteria	Actinomycetales	Intrasporangiaceae	Other	18.43
Actinobacteria	Actinobacteria	Actinomycetales	Propionibacteriaceae	*Propionibacterium*	9.94
Actinobacteria	Acidimicrobiia	Acidimicrobiales	C111	unclassified__C111(f)	7.66
Actinobacteria	Actinobacteria	Actinomycetales	Microbacteriaceae	Other	5.59
Actinobacteria	Thermoleophilia	Gaiellales	Gaiellaceae	unclassified__Gaiellaceae(f)	4.35
Actinobacteria	Actinobacteria	Actinomycetales	Nocardiaceae	*Rhodococcus*	3.52
Actinobacteria	Actinobacteria	Bifidobacteriales	Bifidobacteriaceae	*Bifidobacterium*	3.11
Actinobacteria	Actinobacteria	Actinomycetales	Corynebacteriaceae	*Corynebacterium*	2.48
Actinobacteria	MB-A2-108	0319-7L14	unclassified__0319-7L14(o)	unclassified__0319-7L14(o)	2.28
Actinobacteria	Actinobacteria	Actinomycetales	Micrococcaceae	Other	1.66
Actinobacteria	Actinobacteria	Other	Other	Other	1.45
Actinobacteria	Actinobacteria	Actinomycetales	Nocardioidaceae	*Aeromicrobium*	1.45
Actinobacteria	Actinobacteria	Actinomycetales	Mycobacteriaceae	*Mycobacterium*	1.24
Actinobacteria	Actinobacteria	Actinomycetales	Nocardioidaceae	unclassified__Nocardioidaceae (f)	1.24
Actinobacteria	Actinobacteria	Actinomycetales	Other	Other	1.04
Actinobacteria	Actinobacteria	Actinomycetales	Pseudonocardiaceae	*Pseudonocardia*	1.04
Actinobacteria	Acidimicrobiia	Acidimicrobiales	EB1017	unclassified__EB1017(f)	0.83
Actinobacteria	Actinobacteria	Actinomycetales	Geodermatophilaceae	Other	0.83
Actinobacteria	Actinobacteria	Actinomycetales	unclassified__Actinomycetales (o)	unclassified__Actinomycetales (o)	0.83
Actinobacteria	Acidimicrobiia	Acidimicrobiales	Microthrixaceae	unclassified__Microthrixaceae (f)	0.62
Actinobacteria	Actinobacteria	Actinomycetales	Cellulomonadaceae	Cellulomonas	0.62
Actinobacteria	Actinobacteria	Actinomycetales	Nocardioidaceae	Other	0.62
Actinobacteria	Thermoleophilia	Solirubrobacterales	unclassified__Solirubrobacterales(o)	unclassified__Solirubrobacterales(o)	0.62
Actinobacteria	Actinobacteria	Actinomycetales	Actinomycetaceae	Actinomyces	0.41
Actinobacteria	Actinobacteria	Actinomycetales	Dietziaceae	*Dietzia*	0.41
Actinobacteria	Actinobacteria	Actinomycetales	Geodermatophilaceae	*Geodermatophilus*	0.41
Actinobacteria	Actinobacteria	Actinomycetales	Microbacteriaceae	*Agrococcus*	0.41
Actinobacteria	Actinobacteria	Actinomycetales	Micrococcaceae	*Kocuria*	0.41
Actinobacteria	Actinobacteria	Actinomycetales	Micrococcaceae	*Micrococcus*	0.41
Actinobacteria	Actinobacteria	Actinomycetales	Micrococcaceae	*Rothia*	0.41
Actinobacteria	Actinobacteria	Actinomycetales	Streptomycetaceae	Other	0.41
Actinobacteria	Coriobacteriia	Coriobacteriales	Coriobacteriaceae	*Collinsella*	0.41
Actinobacteria	Actinobacteria	Actinomycetales	Nocardioidaceae	*Friedmanniella*	0.21
Actinobacteria	Actinobacteria	Actinomycetales	Nocardioidaceae	*Nocardioides*	0.21

Table S10 The physiological and biochemical characteristics of *S. pseudogriseolus* A6

Strain	Gram character	Glucose utilization	Starch hydrolysis	Citrate utilization	Nitrate reduction	Oxidase test	Catalase test	Gelatin liquefaction test
A6	+	+	+	-	+	+	+	+

Table S11 Secondary metabolite biosynthesis gene clusters of *S. pseudogriseolus* A6

Cluster ID	Cluster type	Gene number	Most similar known cluster	Ratio of genes similarity (%)
Cluster 1	lanthipeptide-class-i	22	-	-
Cluster 2	T2PKS	69	Spore pigment biosynthetic gene cluster from *Streptomyces avermitilis*	83.00
Cluster 3	terpene	24	Isorenieratene biosynthetic gene cluster from *Streptomyces argillaceus*	100.00
Cluster 4	RiPP-like	6	Informatipeptin biosynthetic gene cluster from *Streptomyces viridochromogenes* DSM 40736	28.00
Cluster 5	RRE-containing	20	Avilamycin A biosynthetic gene cluster from *Streptomyces viridochromogenes* Tue57	5.00
Cluster 6	terpene	23	Carotenoid biosynthetic gene cluster from *Streptomyces avermitilis*	54.00
Cluster 7	T1PKS,NRPS	26	Antimycin biosynthetic gene cluster from *Streptomyces argillaceus*	100.00
Cluster 8	lanthipeptide-class-i	25	Cyphomycin biosynthetic gene cluster from *Streptomyces* sp. ISID311	2.00
Cluster 9	T3PKS	35	Alkylresorcinol biosynthetic gene cluster from *Streptomyces griseus subsp.* griseus NBRC 13350	100.00
Cluster 10	terpene	19	Geosmin biosynthetic gene cluster from *Streptomyces coelicolor* A3(2)	100.00
Cluster 11	RiPP-like	8	-	-
Cluster 12	PKS-like,NRPS	48	Naphthyridinomycin biosynthetic gene cluster from *Streptomyces lusitanus*	92.00
Cluster 13	lanthipeptide-class-iii	19	SapB biosynthetic gene cluster from *Streptomyces coelicolor* A3(2)	100.00
Cluster 14	T2PKS,RiPP-like	58	Xantholipin biosynthetic gene cluster from *Streptomyces flavogriseus*	38.00
Cluster 15	ectoine	10	Ectoine biosynthetic gene cluster from *Streptomyces anulatus*	100.00
Cluster 16	terpene	14	Hopene biosynthetic gene cluster from *Streptomyces coelicolor* A3(2)	61.00
Cluster 17	terpene	16	Albaflavenone biosynthetic gene cluster from *Streptomyces coelicolor* A3(2)	100.00
Cluster 18	NI-siderophore	12	Grincamycin biosynthetic gene cluster from *Streptomyces lusitanus*	11.00
Cluster 19	linaridin	14	Pentostatine biosynthetic gene cluster from *Streptomyces antibioticus*	9.00
Cluster 20	NI-siderophore	9	Desferrioxamin B biosynthetic gene cluster from *Streptomyces coelicolor* A3(2)	83.00
Cluster 21	T1PKS,NRPS-like	13	Caniferolide A biosynthetic gene cluster from *Streptomyces caniferus*	24.00
Cluster 22	T1PKS	1	-	-
Cluster 23	T1PKS,PKS-like	3	Cyphomycin biosynthetic gene cluster from *Streptomyces* sp. ISID311	9.00
Cluster 24	T1PKS	2	Mediomycin A biosynthetic gene cluster from *Kitasatospora mediocidica*	36.00
Cluster 25	T1PKS	3	ECO-02301 biosynthetic gene cluster from *Streptomyces aizunensis*	35.00
Cluster 26	T1PKS	1	-	-
Cluster 27	T1PKS	1	-	-

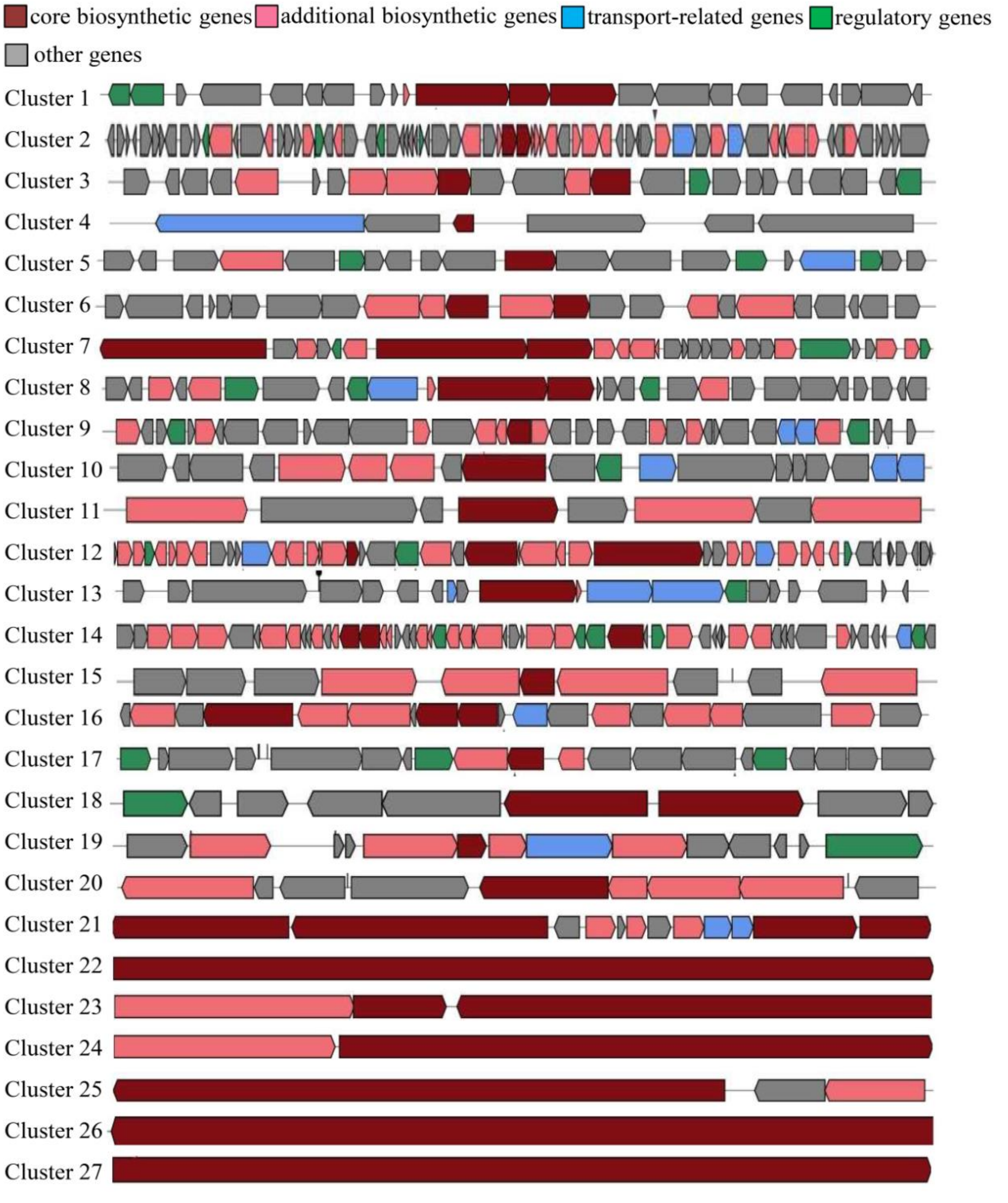

Fig. S1 Prediction of secondary metabolite gene clusters of *S. pseudogriseolus* A6

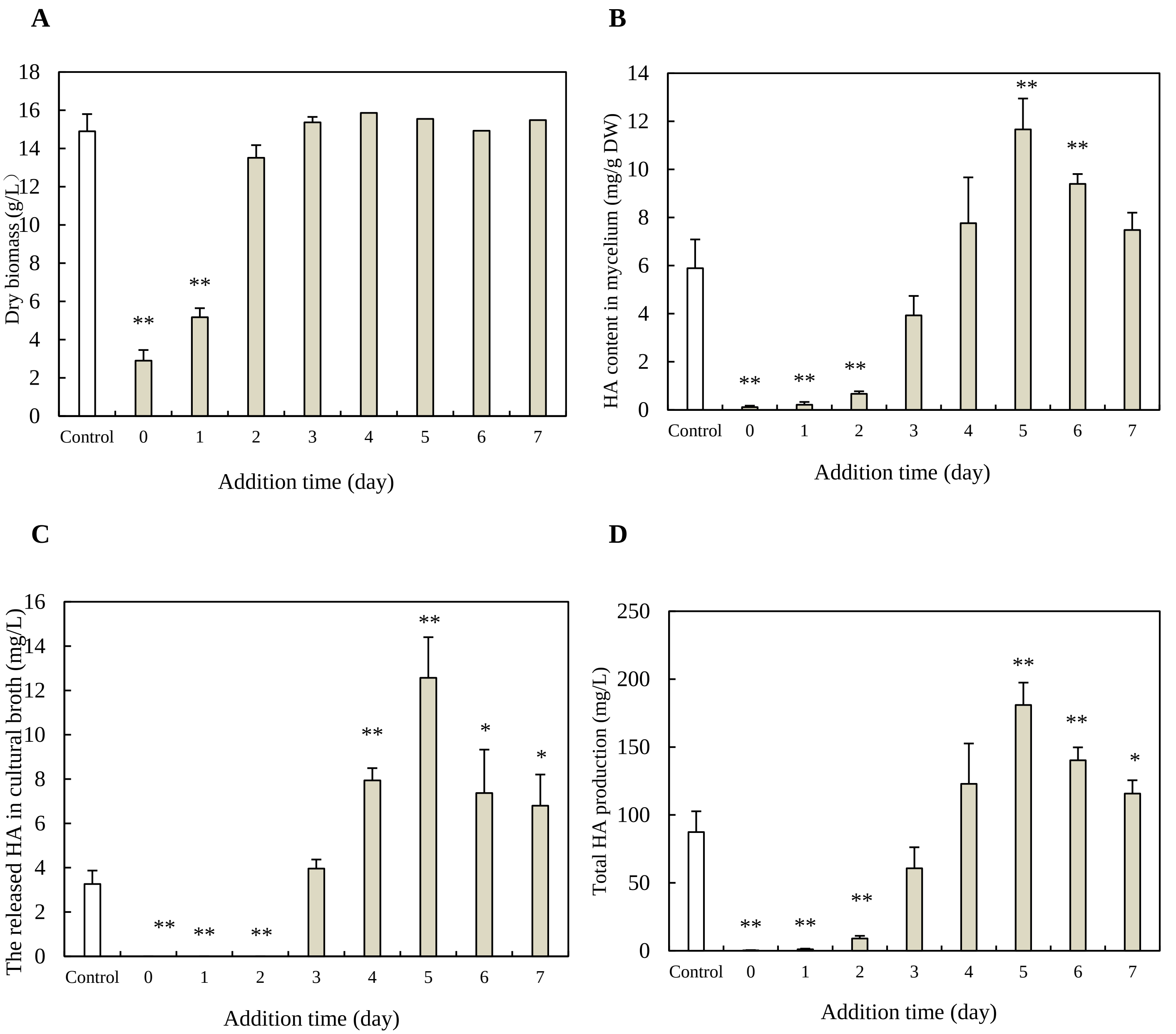

Fig. S2 Effects of introducing time of live *S. pseudogriseolus* A6 on fungal biomass (**A**), hypocrellin A (HA) content in mycelium (**B**), the released HA in cultural broth (**C**) and total HA production (**D**) in submerged culture of *Shiraia* sp. S9. Total HA production refers to the sum of the intracellular and extracellular HA. Live A6 (5.0×10^{6} CFU/mL) was added on different time points and incubated at 150 rpm and 28℃. The mycelia were harvested on day 8. The S9 strain treated with the equal volume of sterile TSB broth instead of live actinobacterial suspension was used as control group. Values are mean ± SD from three independent experiments ($^{*}P < 0.05$, $^{**}P < 0.01$ versus control group)

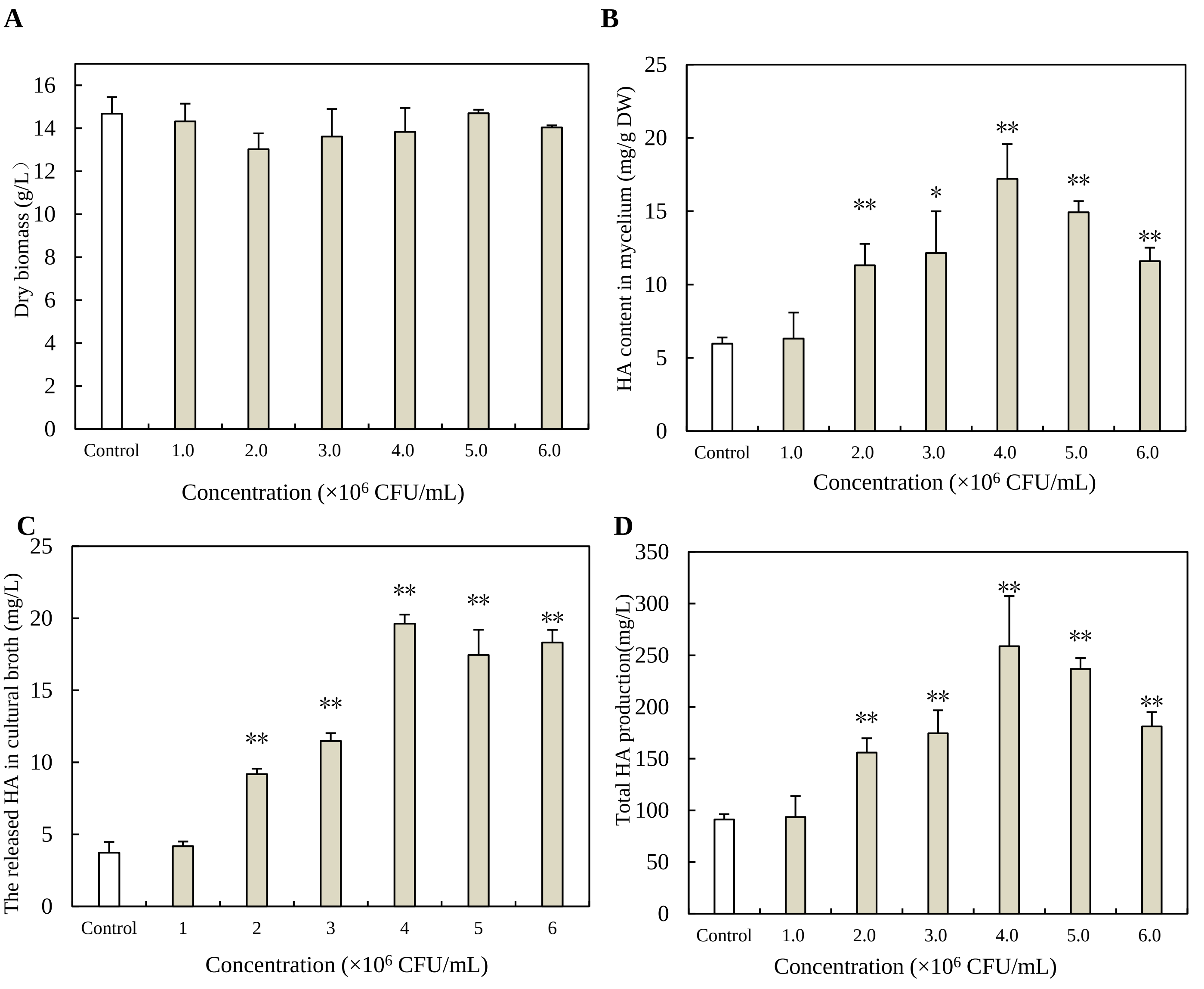

Fig. S3 Effects of different concentrations of live *S. pseudogriseolus* A6 on fungal biomass (**A**), HA content in mycelium (**B**), the released HA in cultural broth (**C**) and total HA production (**D**) in submerged culture of *Shiraia* sp. S9. Total HA production refers to the sum of the intracellular and extracellular HA. Live A6 was added on day 5 of the culture at different volumes at 150 rpm and 28℃. The mycelia were harvested on day 8. The S9 strain treated with the equal volume of sterile TSB broth instead of live actinobacterial suspension was used as control group. Values are mean ± SD from three independent experiments ($^{*}P< 0.05$, $^{**}P< 0.01$ versus control group)
